# Multiple introductions and spread of novel reassortant highly pathogenic avian influenza A (H5N1) clade 2.3.4.4b viruses via wild birds, South Korea, 2024–2025

**DOI:** 10.1186/s13567-026-01847-2

**Published:** 2026-10-07

**Authors:** Ye-Ram Seo, Ha-Eun Lee, Dong-Ju Kim, Young-Jae Si, Sun-Hak Lee, Hyesung Jeong, Suwoong Lee, Chang-Seon Song, Dong-Hun Lee

**Affiliations:** 1 College of Veterinary Medicine, Konkuk University, 120 Neungdong-ro, Seoul, 05029 Republic of Korea; 2 National Institute of Wildlife Disease Control and Prevention, 1 Songam-gil, Gwangsan-gu, Gwangju, 62407 Republic of Korea

**Keywords:** Highly pathogenic avian influenza virus, H5N1, wild bird, South Korea, phylodynamic, phylogeographic analysis

## Abstract

Between 14 October 2024 and 24 March 2025, 43 cases of clade 2.3.4.4b highly pathogenic avian influenza (HPAI) type A (H5N1) infections in wild birds and 1 case in a leopard cat were detected through the national HPAI surveillance program. We performed whole-genome sequencing of 51 isolates and investigated their phylogenetic relationships. Phylogenetic analyses identified nine distinct genotypes, designated Kor24–25A to Kor24–25I. The most commonly detected genotype, Kor24–25A (*n* = 24), was frequently detected in Pelecaniformes and was most prevalent between December 2024 and January 2025. Bayesian continuous and discrete trait phylodynamic analyses using *HA* gene sequences identified the three most probable routes of viral introduction via Gyeonggi, Jeju, and the southern coastal areas of the Korean Peninsula and suggested that wild ducks served as the primary reservoirs facilitating viral transmission in the wild bird population. During the 2024–2025 season, great egrets (*Ardea alba*) appeared to be more frequently affected than other wild bird species, suggesting possible seasonal differences in species-specific disease outcomes among wild birds. This study provides valuable insights into the phylodynamics, host associations, and transmission patterns of HPAI H5N1 viruses. It emphasizes the importance of enhanced surveillance programs targeting key avian hosts and high-risk geographic areas.

**Supplementary information:**

The online version contains Supplementary Material available at https://doi.org/10.1186/s13567-026-01847-2.

## Introduction

The A/Goose/Guangdong/1/1996 (Gs/Gd) lineage of highly pathogenic avian influenza (HPAI) type A (H5) viruses was first identified in domestic geese in China in 1996 and has since caused recurrent outbreaks in both domestic poultry and wild bird populations. Over time, these viruses have diversified into ten genetically distinct hemagglutinin (HA) clades (0–9) and multiple subclades [1]. Among them, clade 2.3.4 of the Gs/Gd lineage H5 HPAI viruses emerged in China in 2008 and subsequently evolved into several subclades, including clade 2.3.4.4a–h [1–4]. In particular, clade 2.3.4.4b viruses underwent extensive reassortment with local avian influenza viruses, generating multiple genotypes, including H5N2, H5N5, H5N6, and H5N8 since 2014 [5]. By late 2021, clade 2.3.4.4b H5N1 viruses had become the predominant HPAI subtype circulating globally, causing widespread outbreaks in wild birds and domestic poultry across Asia, Europe, Africa, and North America [6]. In addition to their substantial impacts on animal health and poultry production, clade 2.3.4.4b H5N1 viruses have raised increasing public health concerns because of their expanded host range and repeated spillover into mammalian species, including humans [7]. As the risk of clade 2.3.4.4b H5N1 influenza pandemic continues to grow with sustained transmission in mammals, it is essential to study viral evolution, geographic spread, and mammalian infection potential of emerging HPAI viruses.

Since the first incursion of HPAI H5N1 viruses in 2003, South Korea has experienced recurrent incursions of HPAI viruses caused by multiple H5Nx subtypes, including H5N1, H5N8, and H5N6 [8]. The epidemiological characteristics of these outbreaks have varied between epidemic seasons, ranging from the shortest outbreak lasting 42 days in 2008 to the longest outbreak of H5N8 lasting 811 days during 2014–2016, which was characterized by multiple viral introductions and the cocirculation of several subgroups [8]. Wild birds have consistently played a key role in the introduction and spread of HPAI viruses in South Korea, particularly for clade 2.3.4.4b H5N1 viruses since 2020. During the 2020–2021 winter season, 234 wild bird cases of clade 2.3.4.4b HPAI viruses were reported, followed by 47 outbreaks in 2021–2022 [9], 174 cases in 2022–2023 [10–12], and 19 cases in 2023–2024 [13, 14], reflecting substantial interseasonal variation in outbreak magnitude and viral activity. These epidemic seasons were also associated with the emergence of distinct viral genotypes, suggesting repeated viral introductions and ongoing reassortment within wild bird populations [10]. Most recently, during the 2024–2025 season, clade 2.3.4.4b H5N1 was introduced into South Korea via wild birds [15]. Furthermore, the detection of the HPAI H5N1 virus in a wild leopard cat (*Prionailurus bengalensis*) in March 2025 represented the first documented spillover event in this wild mammalian species in South Korea, underscoring the expanding host range and continuing threat to animal and public health [16].

During the 2024–2025 winter season, a total of 43 cases of clade 2.3.4.4b HPAI H5N1 infections in wild birds and 1 case in a leopard cat were reported in South Korea. To elucidate the spatiotemporal dispersal pathways, evolutionary dynamics, and host transitions of these viruses, we conducted whole-genome sequencing of H5N1 isolates obtained through the national HPAI surveillance program in wild animals and performed comparative phylogenetic analyses.

## Materials and methods

### Sample collection and virus detection

From October 2024 to March 2025, nationwide wild animal surveillance for HPAI viruses was conducted by the National Institute of Wildlife Disease Control and Prevention (NIWDC). As part of active surveillance, a total of 57,067 fecal samples and 1,007 swab samples from individual birds were collected, across 92 surveillance sites throughout South Korea. In addition, 419 carcasses submitted to the NIWDC were examined through a passive surveillance program. All samples were placed in phosphate-buffered saline supplemented with 400 µg/mL gentamicin and transported under refrigerated conditions. Samples were homogenized by vortexing for 1 min, centrifuged at 3,000 × *g* for 10 min, and subsequently clarified through, a 0.45 µm syringe filter (Minisart^®^, Sartorius; Göttingen, Germany). The filtrates were inoculated into the allantoic cavity of 10-day-old specific-pathogen-free embryonated chicken eggs and incubated at 37 °C for 72 h. After incubation, 25 µL of allantoic fluid was harvested from each egg and tested for hemagglutination activity using 0.5% chicken red blood cell suspension. Hemagglutination-positive allantoic fluids were subjected to viral RNA extraction using the Maxwell^®^ RSC simplyRNA Tissue Kit (Promega; Madison, WI, USA), following the manufacturer’s protocol. Extracted RNA was screened for avian influenza virus by real-time reverse transcription polymerase chain reaction (rRT-PCR) targeting the matrix (*M*) and *H5* genes [17]. During the 2024–2025 winter season in South Korea, 51 viruses were isolated from 43 HPAI outbreaks, with several outbreaks yielding multiple virus isolates.

### Next-generation sequencing and data analysis

For samples testing positive for both the *M* and *H5* genes by rRT-PCR, complementary DNA was synthesized using the SuperScript^TM^ III First-Strand Synthesis System (Invitrogen; Carlsbad, CA, USA). Viral genomes were amplified, as previously described [18]. DNA libraries were prepared with the Illumina DNA Prep Kit (Illumina; San Diego, CA, USA), and 150-bp paired-end sequencing was performed on the Illumina MiSeq platform. Raw reads were trimmed, quality-filtered, and de novo assembled with Clonal Limiting Conditions (CLC) Genomics Workbench 24.0.1 (QIAGEN; Aarhus, Denmark) [19]. Full-length genome sequences of 51 H5N1 HPAI viruses were successfully assembled and deposited in the Global Initiative on Sharing All Influenza Data (GISAID) database (accession numbers; Additional file 1) [20]. DNA barcoding was performed for avian host species identification of HPAI-positive fecal samples through mitochondrial cytochrome oxidase I gene sequencing, as previously described [21]. In addition, an H5N1 isolate obtained from the first documented case of H5N1 HPAI in a wild mammal in South Korea, a leopard cat (*Prionailurus bengalensis*) detected on 16 March 2025 [16], was included in the analysis.

### Phylogenetic and network analyses for genotyping

To assess genotypes and evaluate the temporal signal of the datasets for molecular clock analysis, maximum-likelihood (ML) phylogenetic inference was performed. All viral genomes sequenced in this study were queried against the GISAID database using Basic Local Alignment Search Tool (BLAST) with the top 100 hits initially retrieved for each query. When the nucleotide identity of the 100^th^ hit exceeded 99.0%, the number of retrieved sequences was expanded to 200 or 500 hits to increase sequence diversity. The retrieved sequences were incorporated as references for subsequent analyses. Duplicate entries were removed with ElimDupes [22]. Each gene segment was aligned in multiple alignment using fast Fourier transform (MAFFT) v7.490 [23], and ML phylogenies for polymerase basic 2 (*PB2*), polymerase basic 1 (*PB1*), polymerase acidic (*PA*), *HA*, nucleoprotein (*NP*), neuraminidase (*NA*), *M*, and nonstructural (*NS*) were reconstructed in randomized axelerated maximum likelihood (RAxML) v8.0 [24] under the general time-reversible model with 1,000 bootstrap replicates. Phylogenetic trees were visualized with interactive tree of life (iTOL) [25], and clusters were considered monophyletic when supported by bootstrap values > 70 and nucleotide sequence identities > 98%. In addition to the ML tree-based inference, minimum spanning networks were constructed using concatenated sequences of eight gene segments in population analysis with reticulate trees (PopArt) [26], to elucidate genetic relationships both within and between genotypes. This network analysis connects all sampled genetic variants using the smallest possible number of mutations to visualize exactly how many mutations separate different viruses. Genotypes were also assigned using ggFLU platform [27], with genotype classifications interpreted according to the Genin2 and GenoFlu frameworks. Molecular markers with potential relevance to influenza A virus biology were screened using FluMut [28].

### Bayesian phylodynamic analysis

Bayesian phylodynamic analyses of the *HA* gene were performed using the Bayesian Evolutionary Analysis by Sampling Trees (BEAST) software package version 1.10.4, with Bayesian Evolutionary Analysis Utility (BEAUti) used for input configuration [29]. Based on the ML phylogeny, two major clusters containing the sequences from this study were extracted for phylodynamic analysis to infer transmission dynamics between geographic locations and between host species (Additional file 2). To improve temporal signal suitability for molecular clock analysis, outlier sequences were excluded until the root-to-tip regression showed an *R*^2^ value > 0.5, as assessed using TempEst version 1.5.3 [30]. For the continuous phylogeographic analysis, sampling coordinates were incorporated into a Bayesian framework. A Cauchy relaxed random walk model was then applied to infer viral dispersal patterns in continuous space, following the approach of Dellicour et al. [31]. The tree prior was set to the Skygrid coalescent model, and the nucleotide substitution was modeled using the Hasegawa–Kishino–Yano model with estimated base frequencies. Site heterogeneity was modeled using a gamma distribution. Random jitter was added to the tips with a window size of 0.01 [31]. Each Markov chain length was 50,000,000 and repeated five times.

We investigated spatiotemporal transmission dynamics between host species in South Korea by using a Bayesian discrete trait phylodynamic model, as previously described [10]. The discrete traits used to estimate viral transmission among host species included wild duck (cluster 1, *n* = 6; cluster 2, *n* = 10), egret (cluster 1, *n * = 19; cluster 2, *n* = 3), and others (cluster 1, *n* = 7; cluster 2, *n* = 6). Viruses detected outside of Korea were assigned to an additional category (cluster 1, *n* = 7; cluster 2, *n* = 9), regardless of host species or sampling location, as they were inferred to represent the most probable ancestral origins based on the ML phylogenetic analysis.

All Bayesian phylogenetic analyses were run on the Cyberinfrastructure for Phylogenetic Research (CIPRES) Science Gateway [32], and log files were combined using LogCombiner after confirming effective sample sizes (ESS) > 200 with Tracer. Maximum clade credibility (MCC) trees were generated with TreeAnnotator version 1.10.4 and visualized using FigTree version 1.4.4 [33]. For phylogeography data, viral transmission routes were reconstructed using EvoLaps version 2.42 [34]. For discrete trait analyses, Bayes factors (BF) were calculated with SpreaD3 v1.0.7 [35], and transitions with BF > 3 and posterior probability (PP) > 0.5 were considered significant. Posterior trees were analyzed using Posterior Analysis of Coalescent Trees (PACT) [36] to estimate the number of transition events and the proportion of discrete states over time. As input, we used posterior tree files generated from the discrete trait analysis between host species. The analysis was performed with a temporal bin size of 0.25 years, enabling the extraction of migration numbers and state proportions for each quarter (Q), Q1–4.

## Results

### Multiple genotypes of 2024–2025 HPAI viruses

We successfully sequenced a total of 51 H5N1 HPAI viruses identified in wild animals in South Korea between 14 October 2024 and 24 March 2025. ML phylogenetic analyses revealed several distinct genetic clusters containing the HPAI sequences with bootstrap support values > 90 across all gene segments: *PB2* (three clades), *PB1* (four), *PA* (three), *HA* (two), *NP* (five), *NA* (two), *M* (one), and *NS* (one) (Additional file 3). Within each cluster, most sequences shared > 98% pairwise nucleotide identity, except for three cases, including the *PB1* gene of A/Mandarin duck/Korea/24WF743–8P/2025 (H5N1), and the *NS* genes of A/Wild goose/Korea/24WF894–1P/2025 (H5N1) and A/Mandarin duck/Korea/24WF266–5P/2024 (H5N1). Genotypes were classified according to the genetic constellations of gene segments and their combinations.

**Figure 1:**
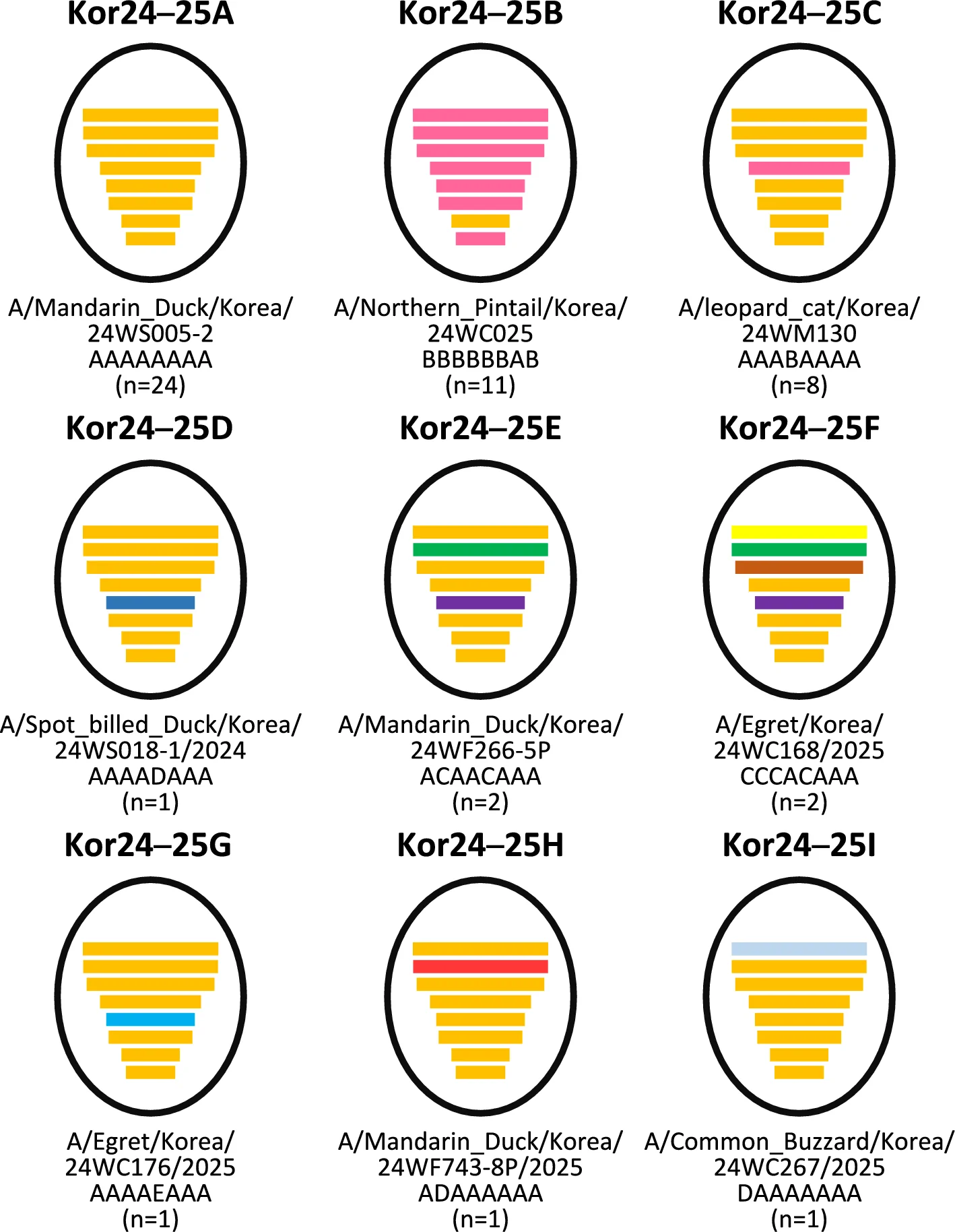
**Genotypes of HPAI H5N1 viruses detected in South Korea during the 2024–2025 winter season.** The eight horizontal bars represent individual HPAI gene segments (*PB2*, *PB1*, *PA*, *HA*, *NP*, *NA*, *M*, and *NS*), and distinct viral origins are represented by different colors. Genotypes are designated by the abbreviation of the country of isolation, surveillance season, and alphabetical identifiers in order from Kor24–25A to Kor24–25I.

Nine distinct genotypes were identified: Kor24–25A (AAAAAAAA; *n* = 24), Kor24–25B (BBBBBBAB; *n* = 11), Kor24–25C (AAABAAAA; *n* = 8), Kor24–25D (AAAADAAA; *n* = 1), Kor24–25E (ACAACAAA; *n* = 2), Kor24–25F (CCCACAAA; *n* = 2), Kor24–25G (AAAAEAAA; *n* = 1), Kor24–25H (ADAAAAAA; *n* = 1), and Kor24–25I (DAAAAAAA; *n* = 1) (Figure 1). To visualize the genetic relationships of viruses within and between genotypes, a minimum spanning network was reconstructed based on concatenated full-genome sequences (Additional file 4). As expected, the network showed that viruses generally clustered according to their assigned genotypes; however, the number of nucleotide substitutions did not always strictly correspond to genotype boundaries. Among the nine identified genotypes, Kor24–25A, Kor24–25B, and Kor24–25C were predominant. The Kor24–25C genotype possessed an *HA* gene closely related to that of Kor24–25B, while its remaining seven gene segments were genetically similar to those of Kor24–25A. The other six genotypes were transient and minor variants, each represented by one or two isolates, and shared most of their gene segments with Kor24–25A but differed in several internal genes.

Notably, Kor24–25D, Kor24–25G, and Kor24–25H possessed *PB1* or *NP* genes derived from previously circulating HPAI lineages in East Asia. In contrast, Kor24–25E, Kor24–25F, and Kor24–25I harbored *PB2* or *NP* genes closely related to low pathogenic avian influenza (LPAI) viruses circulating in central and eastern Asia. In particular, the *PB1* genes of the Kor24–25E and Kor24–25F genotypes formed a distinct cluster with bootstrap support of 100 and showed the highest sequence identity, ranging from 97.5 to 98.0%, to those of European–Russian HPAI viruses (Figure 1; Additional file 3).

Genotype assignment using the ggFlu platform showed different levels of resolution among the analyzed viruses. Viruses belonging to Kor24–25B (*n* = 11) were assigned to the minor genotype B23.2–1 by ggFlu, with corresponding GenoFLU-based minor genotype assignments of A3 and A5 and a GenIn2-based genotype assignment of EA–2020–C. In contrast, the remaining 40 samples were assigned to the minor genotype B.18 by ggFlu but were reported as “not assigned” by both GenIn2 and GenoFLU. FluMut analysis revealed ten distinct mutation-combination profiles (A–J; Additional file 5). Among the phylogenetically defined genotypes, Kor24–25A comprised three mutation-combination types, F, E, and C, while Kor24–25B comprised types G and J, and Kor24–25C comprised types H and I. In contrast, Kor24–25D, Kor24–25E, Kor24–25F, Kor24–25G, Kor24–25H, and Kor24–25I were each associated with a single mutation-combination type, corresponding to F, B, A, F, F, and D, respectively. Among these, type F was shared by four genotypes, Kor24–25A, Kor24–25D, Kor24–25G, and Kor24–25H.

### Temporal and host distributions of HPAI viruses

**Figure 2:**
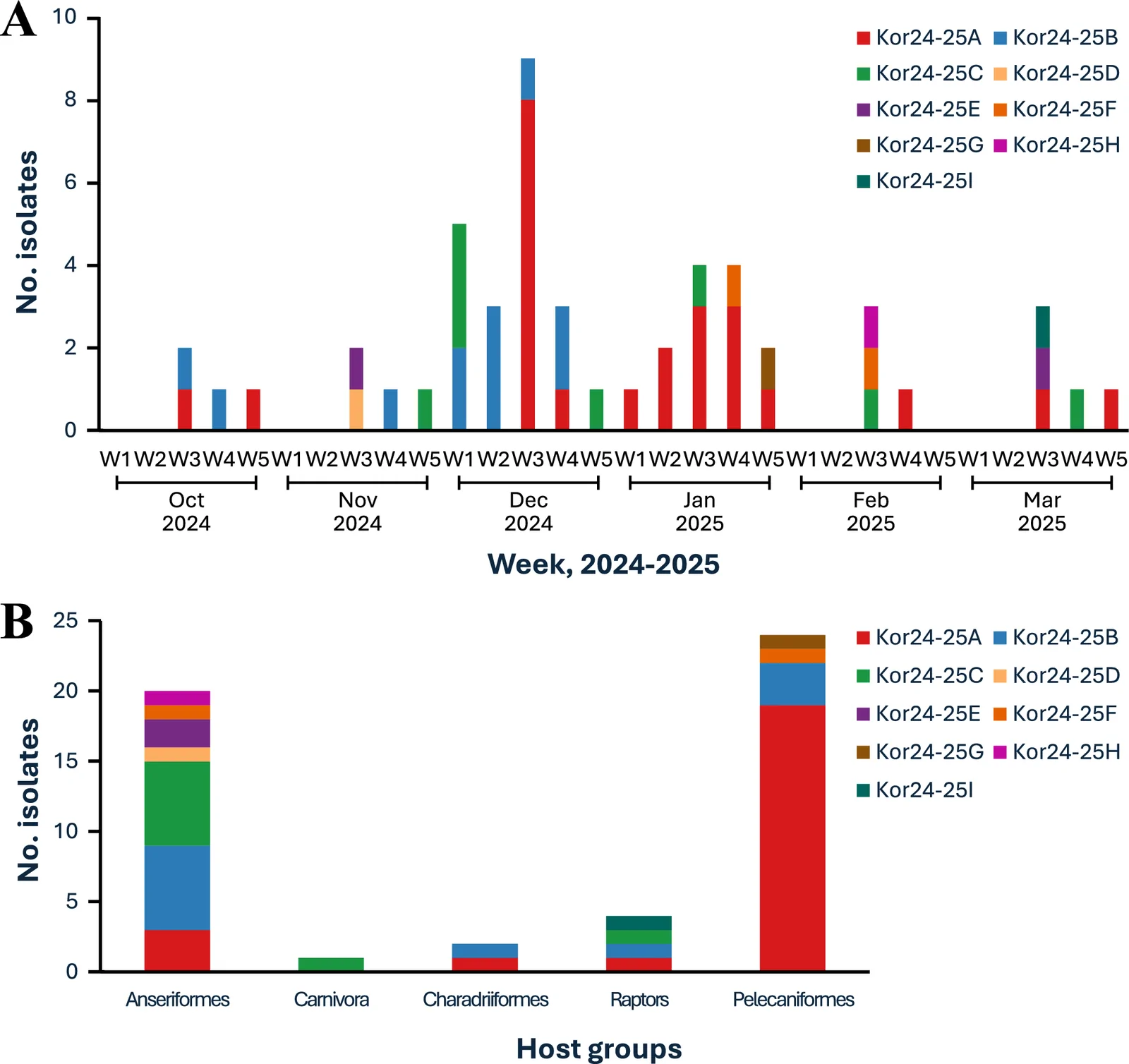
**Distributions of highly pathogenic avian influenza (HPAI) H5N1 genotypes in South Korea, October 2024–March 2025**. **A** Weekly distributions and **B** host group distributions of genotypes.

Between October 2024 and March 2025, the weekly distribution of genotypes was analyzed for 51 HPAI H5N1 viruses (Figure 2A). In the first week of detection, corresponding to the third week of October 2024, Kor24–25A and Kor24–25B were detected concurrently, followed by the emergence of Kor24–25D, Kor24–25E, and Kor24–25C in November 2024 (Figure 2A). The number of HPAI cases in wild birds peaked in December 2024. From the third week of December 2024, the predominant genotype shifted from Kor24–25B to Kor24–25A. While Kor24–25A was consistently detected throughout the season, Kor24–25B was not recorded after December 2024. Minor genotypes were sporadically detected from December 2024 onward.

Analyses of isolate distribution and genotype composition across host taxa revealed that Pelecaniformes accounted for the highest proportion of isolates, followed by Anseriformes, raptors (Falconiformes and Accipitriformes), Charadriiformes, and Carnivora (Figure 2B). Among Pelecaniformes, the great egret (*Ardea alba*) accounted for the highest number of isolates (*n* = 22), predominantly associated with the Kor24–25A genotype. Anseriformes exhibited the greatest genotype diversity among all host groups, with seven distinct genotypes detected. In raptors, four genotypes—Kor24–25A, Kor24–25B, Kor24–25C, and Kor24–25I—were detected. The three predominant genotypes (Kor24–25A, Kor24–25B, and Kor24–25C) were identified across multiple host groups: Kor24–25A and Kor24–25B were detected in all host groups except Carnivora, whereas Kor24–25C was found in Anseriformes, Carnivora, and raptors (Figure 2B).

### Spatiotemporal transmission patterns of HPAI viruses

**Figure 3:**
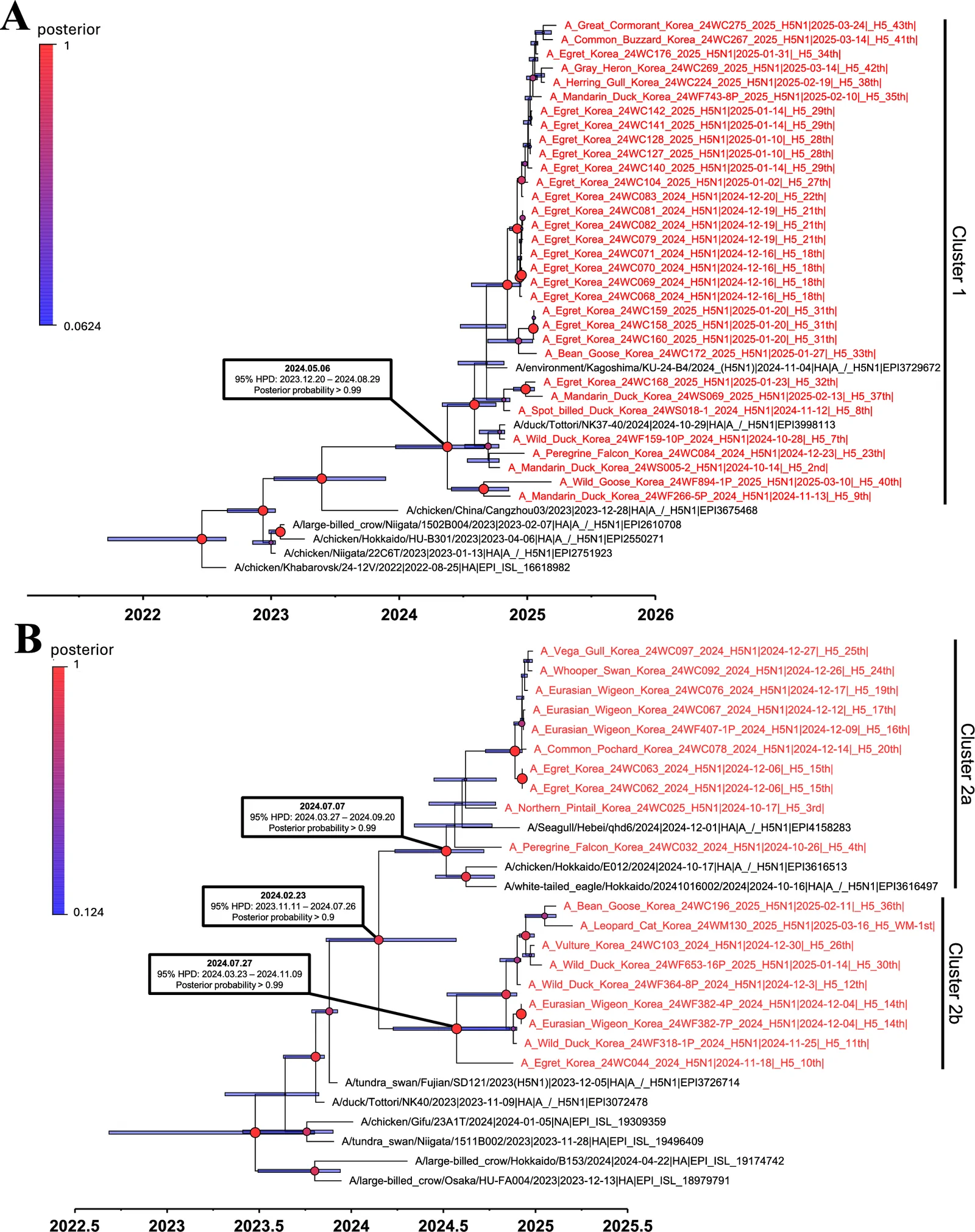
**Maximum clade credibility (MCC) trees of H5N1 viruses detected in South Korea, October 2024–March 2025.** MCC trees include *HA* gene sequences from **A** cluster 1 and **B** cluster 2, with sequences from this study highlighted in red. Node sizes and colors represent posterior probability values, and the timescale is shown in decimal years. The median time to the most recent common ancestor (tMRCA) and the corresponding 95% highest posterior density (HPD) intervals are indicated for each cluster.

**Figure 4:**
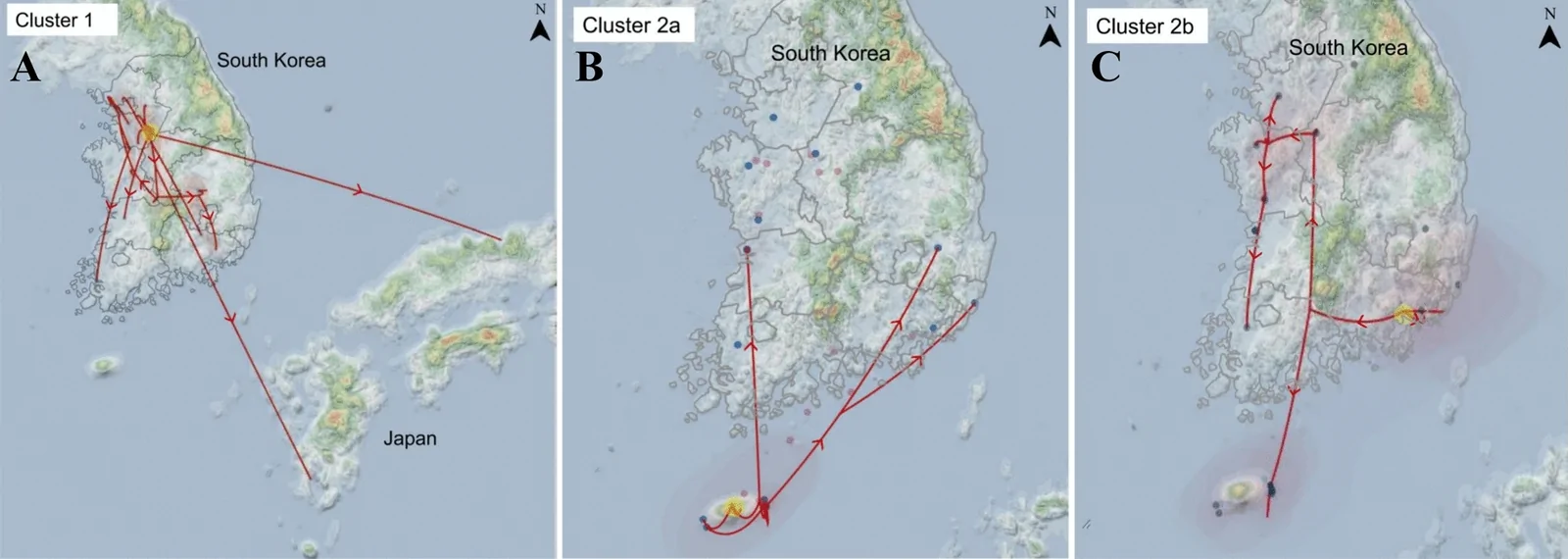
**Visualized transmission routes inferred from continuous trait analyses of cluster 1 and cluster 2**. Routes were subdivided into three: **A** cluster 1, **B** cluster 2a, and **C** cluster 2b. Red arrows indicate the estimated dispersal pathways in EvoLaps, while blue and red dots represent sample collection sites and inferred ancestral locations, respectively. Yellow circles indicate the estimated introduction sites of each cluster.

Bayesian phylodynamic analyses based on the *HA* gene revealed spatiotemporal transmission patterns of H5N1 HPAI viruses detected during the 2024–2025 winter season. The first detected case within cluster 1 was a virus identified on 14 October 2024 from a captured mandarin duck in the Gyeonggi region. Cluster 1, composed of *HA* genes derived from the Kor24–25A genotype (Figure 3), had an estimated time to the most recent common ancestor (tMRCA) of 6 May 2024 ([95% highest posterior density (HPD)]: 20 December 2023–29 August 2024). Continuous phylogeographic analysis suggested that the Gyeonggi region, a central-western plain area of South Korea, was the most probable ancestral location of cluster 1 in South Korea (Figures 3A and 4A). The virus subsequently spread to the southern regions of South Korea, with some viruses moving northward again and others potentially spreading to southern Japan (Figure 4A).

Cluster 2, comprising *HA* genes derived from the Kor24–25B and Kor24–25C genotypes, was divided into cluster 2a, predominantly containing Kor24–25B, and cluster 2b, predominantly containing Kor24–25C, suggesting independent introductions (Figure 3B). The tMRCA for cluster 2a was 7 July 2024 (95% HPD: 27 March 2024–20 September 2024), while that for cluster 2b was 27 July 2024 (95% HPD: 23 March 2024–9 November 2024). The overall tMRCA for cluster 2 was 23 February 2024 (95% HPD: 11 November 2023–26 July 2024) (Figure 3B). The most probable ancestral location of cluster 2a in South Korea was Jeju, a southern island of South Korea. Following introduction, the viruses appeared to spread northward and disperse to the eastern and western regions of South Korea (Figure 4B). The most probable ancestral location of cluster 2b in South Korea was the southern coastal areas of the Korean Peninsula and subsequently appeared to diverge into two possible routes: one spreading northward to the Chungcheong region of South Korea and the other moving southward toward the Jeju region. Viruses that had spread northward later appeared to move southward along the western coastal areas of South Korea (Figure 4C).

### Transmission of HPAI viruses between host species

**Figure 5:**
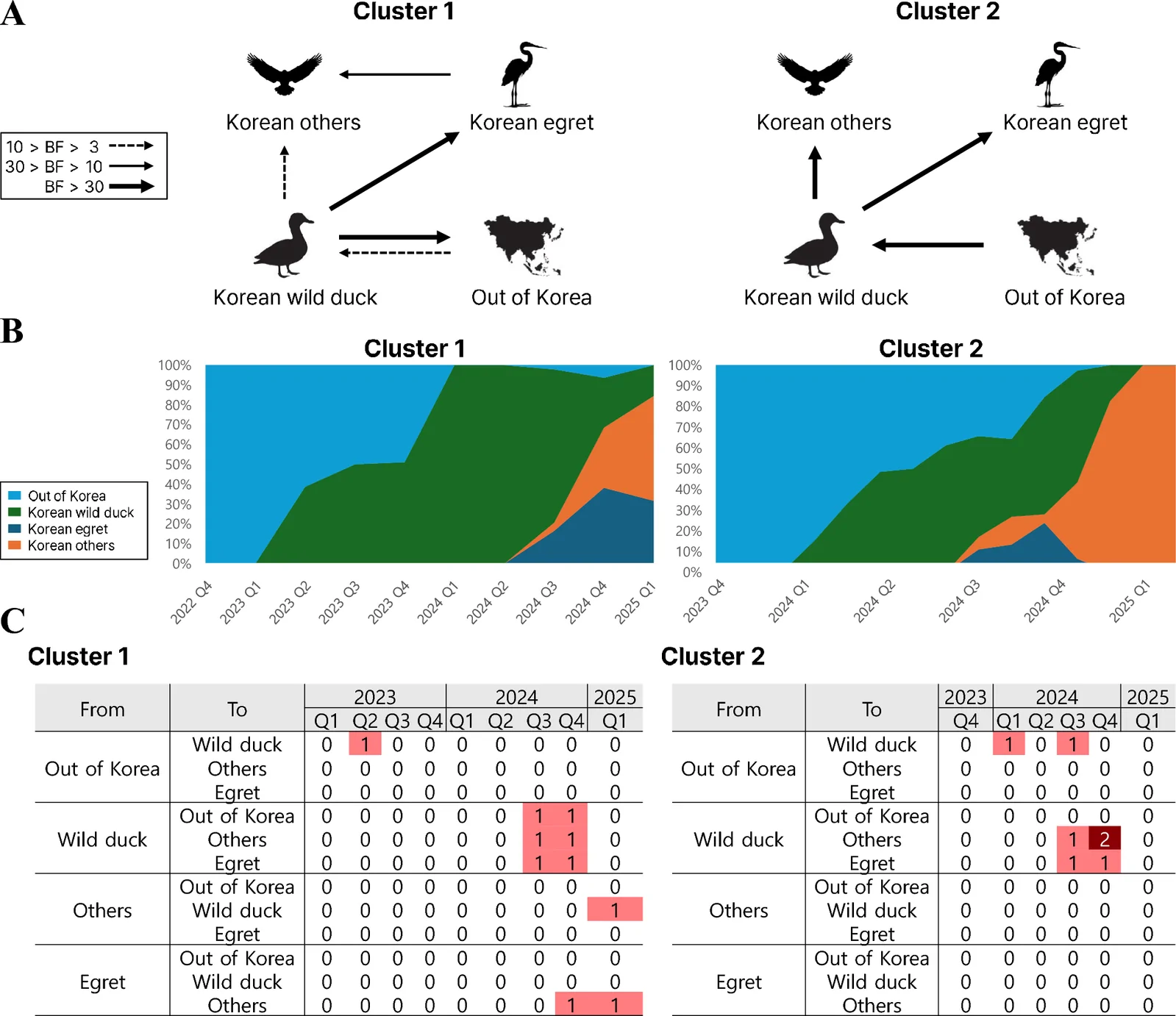
**Transmission dynamics of H5N1 viruses detected in South Korean wild animals, October 2024–March 2025**. **A** Supported transmission routes, with arrow thickness representing the range of Bayes factor values. **B** Proportion of ancestral discrete states estimated along the phylogenetic trunk. **C** Heat map showing the number of transition events between each discrete state.

Based on the host discrete trait analysis, transmission routes with BF > 3 and PP > 0.5 were considered statistically significant, while routes with BF > 20 and PP > 0.9 were considered strongly supported (Additional file 6) [37, 38]. For cluster 1, the virus was most likely introduced via wild ducks (BF = 5.448, PP = 0.708). Subsequent transmission was inferred from wild ducks to egrets (BF = 30.831, PP = 0.932) and to other wild bird species (BF = 5.467, PP = 0.708). In addition, transmission events from egrets to other wild bird species (BF = 23.587, PP = 0.913) and from wild ducks to lineages sampled outside Korea (BF = 43.286, PP = 0.951) were strongly supported (Table 1 and Figure 5A). For cluster 2, similar to cluster 1, the virus was likely introduced from outside Korea via wild ducks (BF = 75.497, PP = 0.971). Subsequent transmission from wild ducks to other wild bird species (BF = 89.554, PP = 0.997) and to egrets (BF = 39.928, PP = 0.947) was strongly supported (Table 2 and Figure 5A). Unlike cluster 1, no transmission originating from egrets was inferred.

**Table 1:** **Supported transmission routes of H5N1 cluster 1 between host types**.

From	To	Bayes factor	Posterior probability	Actual migration rate
Out of Korea	Korean wild duck	5.448	0.708	0.528
Korean wild duck	Out of Korea	43.286	0.951	1.064
Korean wild duck	Korean others	5.467	0.708	0.824
Korean wild duck	Korean egret	30.831	0.932	1.131
Korean egret	Korean others	23.587	0.913	1.316

Routes with Bayes factor > 3 and posterior probability > 0.5 are shown.

**Table 2:** **Supported transmission routes of H5N1 cluster 2 between host types**.

From	To	Bayes factor	Posterior probability	Actual migration rate
Out of Korea	Korean wild duck	75.497	0.971	1.068
Korean wild duck	Korean others	89.554	0.997	1.739
Korean wild duck	Korean egret	39.928	0.947	1.136

Routes with Bayes factor > 3 and posterior probability > 0.5 are shown.

Analysis of the proportions of ancestral discrete states estimated on the phylogenetic trunk showed that, for both cluster 1 and cluster 2, the highest trunk proportions shifted over time in the following sequence: out of Korea, Korean wild duck, Korean egret, and Korean others (Figure 5B). Our source–sink dynamics showed that transition events for both cluster 1 and cluster 2 occurred during Q3 2024, from wild ducks to egrets and other species. In cluster 1, additional transition events from egrets to other species were observed during Q4 2024 and Q1 2025 (Figure 5C). Based on the density distribution of total time (Markov rewards) among Korean host groups, cluster 1 showed the highest mean Markov reward time in wild ducks [2.6969 (95% HPD: 0.6977–4.671)], followed by egrets [2.4019 (95% HPD: 0.9754–4.734)] and other wild bird species [1.1038 (95% HPD: 0.3508–2.2372)]. In cluster 2, wild ducks also exhibited the highest mean value [2.2149 (95% HPD: 0.9727–3.5273)], followed by other wild bird species [0.6459 (95% HPD: 0.2886–1.0832)] and egrets [0.3292 (95% HPD: 0.0128–0.8016)] (Additional file 7).

## Discussion

Here, we report nine novel HPAI genotypes found in wild birds during the 2024–2025 winter season, suggesting frequent reassortment between clade 2.3.4.4b HPAI viruses and cocirculating HPAI or LPAI strains. Among the viral internal genes, the *M* and *NS* genes showed the lowest genetic diversity, whereas the *NP* gene formed the largest number of distinct monophyletic clades. Kor24–25A, Kor24–25B, and Kor24–25C were the predominant genotypes, although their temporal distributions varied. Kor24–25A persisted throughout the entire surveillance period, while Kor24–25B was detected only during the early phase until the fifth week of December 2024, accounting for approximately half of the sampling period. This transient dominance of early-season genotypes has been observed in previous outbreaks in South Korea [10]. However, unlike the 2021–2022 and 2022–2023 winter seasons, where genotypic diversity decreased as outbreaks progressed, the current dataset showed sustained genetic diversity throughout the 2024–2025 outbreak period [10, 39].

In addition, Kor24–25A, Kor24–25B, and Kor24–25C genotypes were all detected in multiple wild bird species, indicating a broad host range. Among host groups, Anseriformes showed the greatest genotype diversity, consistent with their role as major reservoir hosts for wild aquatic birds [40]. The greater genotype diversity observed in Anseriformes may reflect their broad migratory connectivity and congregation at shared wintering sites, where aquatic birds frequently share habitats. In addition, inferred transmission from egrets to other wild bird species within cluster 1 suggests that egrets may have contributed not only as susceptible hosts but also as potential intermediate hosts involved in local viral dissemination [41, 42]. This close interspecies contact during the wintering season possibly promotes virus transmission and reassortment events among genetically diverse HPAI strains between different aquatic bird species. Raptors showed the second highest genotype diversity, differing from previous reports describing low or undetectable avian influenza virus prevalence [43, 44]. This could be explained by sporadic infections in predators resulting from the consumption of infected waterfowl [45]. Notably, the sporadic infection of Kor24–25C in a leopard cat likely resulted from consumption of infected wild birds, as suggested in the previous study [46].

Bayesian phylodynamic analyses of the *HA* gene identified three distinct viral clusters, indicating at least three independent introduction events. The region where cluster 1 was introduced has been identified as a high-risk area for HPAI incursion in previous studies, with greater introduction risk from countries such as Russia, Mongolia, and China rather than Japan [10, 47]. In contrast, the areas where cluster 2 was introduced—Jeju island in southern South Korea and the southern regions of the Korean Peninsula—are known to have higher introduction risk from Japan [48]. This possibility is supported by a phylogenetic analysis using *HA* gene sequences of H5 HPAI viruses isolated in Japan during the winter of 2024–2025, which showed that 24WC025, a strain from cluster 2a, clustered within the same clade as the Japanese isolates from the same period [49]. Given that cluster 2a and cluster 2b were probably introduced independently via wild birds to different regions, and all identified gene sequences were closely related to viruses previously detected in wild birds, reassortment involving Kor24–25A- and Kor24–25B-derived *HA* genes was more likely to have occurred within migratory wild bird populations in Asia rather than in domestic poultry.

Discrete trait analysis of host dynamics based on the *HA* gene suggested season-specific differences in the distribution and transmission patterns of HPAI viruses among wild bird species in South Korea. Previous studies suggested that wild ducks played a major role during the early phase of viral introduction and domestic spread, while geese contributed more substantially to subsequent dissemination within the country [10]. However, during the 2024–2025 season, goose-derived viruses accounted for only 5.9% (3/51) of the analyzed isolates, which may reflect reduced mortality or carcass detection in geese. In contrast, egret-derived viruses represented 43.13% (22/51) of the analyzed isolates, a markedly higher proportion compared with previous HPAI outbreaks in South Korea [10, 48]. Based on these observations, we assume that egrets may have been more frequently affected during the 2024–2025 season compared with previous HPAI outbreaks. Interestingly, whereas large-scale outbreaks among cranes, including hooded cranes and white-naped cranes, were reported during the 2022–2023 season [50], no HPAI-positive cranes were identified during the 2024–2025 season, indicating substantial seasonal differences in affected host species during HPAI outbreaks. These findings highlight seasonal differences in host-associated transmission dynamics and support the need for continued multispecies surveillance and pathobiological studies in wild birds.

Overall, Bayesian phylodynamic analyses of the *HA* gene characterized multiple introduction routes of HPAI H5N1 viruses into South Korea, genotype-specific transmission patterns within the country, and the pivotal roles of wild ducks and egrets in virus dissemination. The observation of multiple introduction routes into South Korea and frequent re-assortment events underscores the complexity of HPAI epidemiology in the region. These findings provide valuable insights into the transmission dynamics of HPAI H5N1 viruses and emphasize the importance of enhanced surveillance programs targeting key avian hosts and high-risk geographic areas. However, analyses of wild-bird-derived viruses should be interpreted with caution, as surveillance may not fully represent the diversity of wild bird populations and may be biased toward larger and more abundant species or those exhibiting higher pathogenicity [41, 42].

Future integrative approaches incorporating pathobiology, wild bird ecology, and migratory bird movement data will further advance understanding of the ecology and epidemiology of clade 2.3.4.4b HPAI in wild birds and improve preparedness for future outbreaks.

## Supplementary information

**Additional file 1** **Detailed information on HPAI A(H5N1) virus isolates from wild animals in South Korea, October 2024–March 2025**. Collection date, sample ID, geographic region, sample type, host species, subtype, and GISAID isolate ID for the 51 H5N1 virus isolates analyzed in this study.

**Additional file 2 Composition of maximum clade credibility (MCC) tree datasets used for phylogenetic and discrete trait analyses of HPAI H5N1 viruses.** Virus sequences and corresponding GISAID isolate IDs included in the MCC tree datasets for cluster 1 (*n* = 39) and cluster 2 (*n* = 28), categorized according to the discrete host traits used in the analyses.

 **Additional file 3** **Eight maximum-likelihood (ML) trees (p-distance, 98%) for each segment of 51 H5N1 viruses.** Maximum-likelihood phylogenetic trees constructed using sequences obtained from the GISAID database for the (A) *PB2*, (B) *PB1*, (C) *PA*, (D) *HA*, (E) *NP*, (F) *NA*, (G) *M*, and (H) *NS* gene segments. Colored vertical bars indicate the segment-specific genetic groups used to define genotype constellations, using the same color scheme as the corresponding gene segments in Figure 1.

 **Additional file 4 Minimum spanning network constructed in PopArt software using concatenated sequences of eight gene segments.** Genetic distances between samples are indicated by numbers, and samples with identical sequences are represented by circle size. Sequences are grouped according to their assigned genotypes and compartmentalized with boxes. Circle colors represent host groups, and genome constellations and corresponding genotype designations are shown within the boxes. Host names and sample ID numbers are indicated around the circles, with egret-derived viruses highlighted in red.

 **Additional file 5** **FluMut-based mutation profiles of viruses analyzed in this study.** Viruses were classified into ten mutation-combination types, designated A–J, based on the combination of mutations detected by FluMut. The table lists the gene, amino acid mutation, reported biological effect, and presence or absence of each mutation across mutation-combination types A–J.

 **Additional file 6 Maximum clade credibility (MCC) trees with discrete traits of (A) cluster 1 and (B) cluster 2 of HPAI H5N1 viruses detected between October 2024 and March 2025 in South Korea.** Maximum clade credibility trees showing the inferred discrete host states for cluster 1 and cluster 2. Node colors represent posterior probabilities, branch colors represent the inferred discrete host states, and the time axis is shown in decimal years.

 **Additional file 7 Density distribution of the total time spent in each discrete host state (Markov reward time) for (A) cluster 1 and (B) cluster 2 of HPAI H5N1 viruses detected in South Korea.** Density distributions of Markov reward times representing the total time spent in the Korean egret, Korean others, and Korean wild duck states for cluster 1 and cluster 2.

## Data Availability

No datasets were generated or analyzed during the current study.

## References

[1] Lee DH, Bertran K, Kwon JH, Swayne DE (2017) Evolution, global spread, and pathogenicity of highly pathogenic avian influenza H5Nx clade 2.3.4.4. J Vet Sci 18:269–280 10.4142/jvs.2017.18.S1.269 PMC5583414 28859267

[2] Lycett SJ, Pohlmann A, Staubach C, Caliendo V, Woolhouse M, Beer M, Kuiken T, Global Consortium for H5N8 and Related Influenza Viruses (2020) Genesis and spread of multiple reassortants during the 2016/2017 H5 avian influenza epidemic in Eurasia. Proc Natl Acad Sci U S A 117:20814–20825 10.1073/pnas.2001813117 PMC7456104 32769208

[3] World Health Organization (2021) Antigenic and genetic characteristics of zoonotic influenza A viruses and development of candidate vaccine viruses for pandemic preparedness. https://cdn.who.int/media/docs/default-source/influenza/who-influenza-recommendations/vcm-southern-hemisphere-recommendation-2022/202110_zoonotic_vaccinevirusupdate.pdf?sfvrsn=8f87a5f1_1. Accessed 20 August 2026

[4] Kwon JH, Bertran K, Lee DH, Criado MF, Killmaster L, Pantin-Jackwood MJ, Swayne DE (2023) Diverse infectivity, transmissibility, and pathobiology of clade 2.3.4.4 H5Nx highly pathogenic avian influenza viruses in chickens. Emerg Microbes Infect 12:2218945 10.1080/22221751.2023.2218945 PMC10262800 37309051

[5] Xie Z, Yang J, Jiao W, Li X, Iqbal M, Liao M, Dai M (2025) Clade 2.3.4.4b highly pathogenic avian influenza H5N1 viruses: knowns, unknowns, and challenges. J Virol 99:e00424-25 10.1128/jvi.00424-25 PMC12172449 40340397

[6] Verhagen JH, Fouchier RA, Lewis N (2021) Highly pathogenic avian influenza viruses at the wild–domestic bird interface in Europe: future directions for research and surveillance. Viruses 13:212 10.3390/v13020212 PMC7912471 33573231

[7] Graziosi G, Lupini C, Catelli E, Carnaccini S (2024) Highly pathogenic avian influenza (HPAI) H5 clade 2.3.4.4b virus infection in birds and mammals. Animals (Basel) 14:1372 10.3390/ani14091372 PMC11083745 38731377

[8] Lee SH, Kwon JH, Youk S, Lee SW, Lee DH, Song CS (2025) Epidemiology and pathobiology of H5Nx highly pathogenic avian influenza in South Korea (2003–2024): a comprehensive review. Vet Q 45:23–38 10.1080/01652176.2025.2498918 PMC12064103 40332021

[9] Sagong M, Lee YN, Song S, Cha RM, Lee EK, Kang YM, Cho HK, Kang HM, Lee YJ, Lee KN (2022) Emergence of clade 2.3.4.4b novel reassortant H5N1 high pathogenicity avian influenza virus in South Korea during late 2021. Transbound Emerg Dis 69:e3255–e3260 10.1111/tbed.14551 35413157

[10] Seo YR, Cho AY, Si YJ, Lee SI, Kim DJ, Jeong H, Kwon JH, Song CS, Lee DH (2024) Evolution and spread of highly pathogenic avian influenza A (H5N1) clade 2.3.4.4b virus in wild birds, South Korea, 2022–2023. Emerg Infect Dis 30:299–309 10.3201/eid3002.231274 PMC10826760 38215495

[11] Animal and Plant Quarantine Agency (2023) Highly pathogenic avian influenza detection status. https://www.qia.go.kr/viewwebQiaCom.do?id=60620&type=2_12qlgzls. Accessed 20 August 2026

[12] Seo YR, Lee SH, Jeong S, Cho H, Kim D, Kim DJ, Si YJ, Jeong H, Lee S, Song CS, Swayne DE (2024) Genetic and pathological analysis of hooded cranes (*Grus monacha*) naturally infected with clade 2.3.4.4b highly pathogenic avian influenza H5N1 virus in South Korea in the winter of 2022. Front Vet Sci 11:1499440 10.3389/fvets.2024.1499440 PMC11576466 39568482

[13] Cho AY, Si YJ, Lee DY, Kim DJ, Kim D, Jeong H, Song CS, Lee DH (2024) Index case of H5N1 clade 2.3.4.4b highly pathogenic avian influenza virus in wild birds, South Korea, November 2023. Front Vet Sci 18:1366082 10.3389/fvets.2024.1366082 PMC11064161 38699674

[14] Heo GB, Kang YM, An SH, Kim Y, Cha RM, Jang Y, Lee EK, Lee YJ, Lee KN (2024) Concurrent infection with clade 2.3.4.4b highly pathogenic avian influenza H5N6 and H5N1 viruses, South Korea, 2023. Emerg Infect Dis 30:1223–1227 10.3201/eid3006.240194 PMC11138991 38703023

[15] Si YJ, Kim DJ, Lee SH, Seo YR, Jeong H, Lee S, Lee DH (2025) New incursions of H5N1 clade 2.3.4.4b highly pathogenic avian influenza viruses in wild birds, South Korea, October 2024. Front Vet Sci 10:1526118 10.3389/fvets.2024.1526118 PMC11758627 39867599

[16] Si YJ, Lee SH, Kim DJ, Lee K, Lee MA, Lee DY, Seo YR, Jeong H, Lee S, Lee DH (2025) First detection of clade 2.3.4.4b H5N1 highly pathogenic avian influenza virus in a wild leopard cat (*Prionailurus bengalensis*) in South Korea. Front Vet Sci. 12:1638067 10.3389/fvets.2025.1638067 PMC12341388 40800228

[17] Spackman E (2020) Avian influenza virus detection and quantitation by real-time RT-PCR. In: Spackman E (ed) Animal influenza virus: methods and protocols. Springer, New York, NY, pp 137–148 10.1007/978-1-0716-0346-8_11 32170686

[18] Lee DH (2020) Complete genome sequencing of influenza A viruses using next-generation sequencing. In: Spackman E (ed) Animal influenza virus: methods and protocols. Springer, New York, NY, pp 69–79 10.1007/978-1-0716-0346-8_6 32170681

[19] Qiagen C L C (2026) Genomics workbench. https://www.qiagen.com. Accessed 20 August 2026

[20] Shu Y, McCauley J (2017) GISAID: global initiative on sharing all influenza data—from vision to reality. Euro Surveill 22:30494 10.2807/1560-7917.ES.2017.22.13.30494 PMC5388101 28382917

[21] Lee DH, Lee HJ, Lee YJ, Kang HM, Jeong OM, Kim MC, Kwon JS, Kwon JH, Kim CB, Lee JB (2010) DNA barcoding techniques for avian influenza virus surveillance in migratory bird habitats. J Wildl Dis 46:649–654 10.7589/0090-3558-46.2.649 20688667

[22] Los Alamos (2026) National laboratory HIV sequence database elimdupes. https://www.hiv.lanl.gov/content/sequence/elimdupesv2/elimdupes.html. Accessed 20 August 2026

[23] Katoh K, Rozewicki J, Yamada KD (2019) MAFFT online service: multiple sequence alignment, interactive sequence choice and visualization. Brief Bioinform 20:1160–1166 10.1093/bib/bbx108 PMC6781576 28968734

[24] Stamatakis A (2014) RAxML version 8: a tool for phylogenetic analysis and post-analysis of large phylogenies. Bioinformatics 30:1312–1313 10.1093/bioinformatics/btu033 PMC3998144 24451623

[25] Letunic I, Bork P (2024) Interactive tree of life (iTOL) v6: recent updates to the phylogenetic tree display and annotation tool. Nucleic Acids Res 52:W78–W82 10.1093/nar/gkae268 PMC11223838 38613393

[26] Leigh JW, Bryant D (2015) POPART: full-feature software for haplotype network construction. Methods Ecol Evol 6:1110–1116

[27] Marandino A, Tomás G, Panzera Y, Uriarte V, Russi V, Pérez R, Bassetti L, Negro R, Rodríguez S, Pérez R (2026) Reassortant high pathogenicity avian influenza A (H5N1) viruses during the reemergence in uruguay suggest increasing genetic diversity in South America. Viruses 18:558 10.3390/v18050558 PMC13211659 42198761

[28] Giussani E, Sartori A, Salomoni A, Cavicchio L, de Battisti C, Pastori A, Varotto M, Zecchin B, Hughes J, Monne I, Fusaro A (2025) FluMut: a tool for mutation surveillance in highly pathogenic H5N1 genomes. Virus Evol 11:veaf011 10.1093/ve/veaf011 PMC11908534 40093844

[29] Suchard MA, Lemey P, Baele G, Ayres DL, Drummond AJ, Rambaut A (2018) Bayesian phylogenetic and phylodynamic data integration using BEAST 1.10. Virus Evol 4:vey016 10.1093/ve/vey016 PMC6007674 29942656

[30] Rambaut A, Lam TT, Carvalho LM, Pybus OG (2016) Exploring the temporal structure of heterochronous sequences using TempEst (formerly Path-O-Gen). Virus Evol 2:vew007 10.1093/ve/vew007 PMC4989882 27774300

[31] Dellicour S, Gill MS, Faria NR, Rambaut A, Pybus OG, Suchard MA, Lemey P (2021) Relax, keep walking—a practical guide to continuous phylogeographic inference with BEAST. Mol Biol Evol 38:3486–3493 10.1093/molbev/msab031 PMC8321535 33528560

[32] Miller MA, Pfeiffer W, Schwartz T (2010) Creating the CIPRES Science Gateway for inference of large phylogenetic trees. Gateway Comput Environ Workshop (GCE). 10.1109/GCE.2010.5676129

[33] Rambaut A (2018) FigTree. Institute of Evolutionary Biology, University of Edinburgh. http://tree.bio.ed.ac.uk/software/figtree/. Accessed 20 August 2026

[34] Chevenet F, Fargette D, Bastide P, Vitré T, Guindon S (2024) EvoLaps 2: advanced phylogeographic visualization. Virus Evol 10:078 10.1093/ve/vead078 PMC10771281 38188280

[35] Bielejec F, Rambaut A, Suchard MA, Lemey P (2011) SPREAD: spatial phylogenetic reconstruction of evolutionary dynamics. Bioinformatics 27:2910–2912 10.1093/bioinformatics/btr481 PMC3187652 21911333

[36] Bedford T (2011). PACT: Posterior Analysis of Coalescent Trees. GitHub. https://github.com/trvrb/PACT. Accessed 20 August 2026

[37] Lemey P, Rambaut A, Drummond AJ, Suchard MA (2009) Bayesian phylogeography finds its roots. PLoS Comput Biol 5:e1000520 10.1371/journal.pcbi.1000520 PMC2740835 19779555

[38] Kass RE, Raftery AE (1995) Bayes factor and model uncertainty. J Am Stat Assoc 90:773–795

[39] Cha RM, Lee YN, Park MJ, Baek YG, Shin JI, Jung CH, Sagong M, Heo GB, Kang YM, Lee KN (2023) Genetic characterization and pathogenesis of H5N1 high pathogenicity avian influenza virus isolated in South Korea during 2021–2022. Viruses 15:1403 10.3390/v15061403 PMC10304347 37376703

[40] Fusaro A, Zecchin B, Giussani E, Palumbo E, Agüero-García M, Bachofen C, Bálint Á, Banihashem F, Banyard AC, Beerens N, Bourg M, Briand FX, Bröjer C, Brown IH, Brugger B, Byrne AMP, Cana A, Christodoulou V, Dirbakova Z, Fagulha T, Fouchier RAM, Garza-Cuartero L, Georgiades G, Gjerset B, Grasland B, Groza O, Harder T, Henriques AM, Hjulsager CK, Ivanova E, Janeliunas Z, et al. (2024) High pathogenic avian influenza A (H5) viruses of clade 2.3.4.4b in Europe—why trends of virus evolution are more difficult to predict. Virus Evol 10:veae027 10.1093/ve/veae027 PMC11065109 38699215

[41] Olsen B, Munster VJ, Wallensten A, Waldenström J, Osterhaus ADME, Fouchier RAM (2006) Global patterns of influenza A virus in wild birds. Science 312:384–388 10.1126/science.1122438 16627734

[42] Spackman E (2009) The ecology of avian influenza virus in wild birds: what does this mean for poultry? Poult Sci 88:847–850 10.3382/ps.2008-00336 19276433

[43] Brown JD, Luttrell MP, Berghaus RD, Kistler W, Keeler SP, Howey A, Wilcox B, Hall J, Niles L, Dey A (2010) Prevalence of antibodies to type A influenza virus in wild avian species using two serologic assays. J Wildl Dis 46:896–911 10.7589/0090-3558-46.3.896 PMC11345711 20688695

[44] Eriksson P, Lindskog C, Lorente-Leal V, Waldenström J, González-Acuna D, Järhult JD, Lundkvist Å, Olsen B, Jourdain E, Ellström P (2019) Attachment patterns of human and avian influenza viruses to trachea and colon of 26 bird species—support for the community concept. Front Microbiol 10:815 10.3389/fmicb.2019.00815 PMC6482220 31057520

[45] Sims LD, Weaver J, Swayne DE (2016) Epidemiology of avian influenza in agricultural and other man-made systems. In: Swayne DE (ed) Animal influenza, 2nd edn. Wiley-Blackwell, Hoboken, NJ, pp 302–336

[46] Alexakis L, Fusaro A, Kuiken T, Mirinavičiūtė G, Ståhl K, Staubach C, Svartström O, Terregino C (2024) European Food Safety Authority, European Centre for Disease Prevention and Control, European Union Reference Laboratory for Avian Influenza—avian influenza overview March–June 2024. EFSA J 22:e8930 10.2903/j.efsa.2024.8930 PMC11258884 39036773

[47] Yoo DS, Lee K, Beatriz ML, Chun BC, Belkhiria J, Lee KN (2022) Spatiotemporal risk assessment for avian influenza outbreak based on the dynamics of habitat suitability for wild birds. Transbound Emerg Dis 69:e953–e967 10.1111/tbed.14376 34738338

[48] Seo YR, Cho AY, Kim DJ, Si YJ, Jeong HS, Lee SW, Song CS, Lee DH (2025) Transmission dynamics of highly pathogenic avian influenza A (H5N1) and A (H5N6) viruses in wild birds, South Korea, 2023–2024. Emerg Infect Dis 31:1561–1572 10.3201/eid3108.250373 PMC12309777 40633556

[49] Isoda N, Hew LY, Nishikawa K, Takaya F, Shimazu Y, Kobayashi D, Nabeshima K, Honjyo H, Esaki M, Okuya K (2025) Genetic diversity of highly pathogenic avian influenza viruses isolated in Hokkaido, Japan, during winter 2024–2025. Pathogens (Basel) 14:951 10.3390/pathogens14090951 PMC12472925 41011850

[50] Esaki M, Okuya K, Tokorozaki K, Haraguchi Y, Hasegawa T, Ozawa M (2025) Highly pathogenic avian influenza A (H5N1) outbreak in endangered cranes, Izumi Plain, Japan, 2022–23. Emerg Infect Dis 31:937–947 10.3201/eid3105.241410 PMC12044252 40305301

