# Supplementary material for: Multiple introductions and spread of novel reassortant highly pathogenic avian influenza A (H5N1) clade 2.3.4.4b viruses via wild birds, South Korea, 2024–2025
Source: Vet Res. 2026 Oct 7;57:178. doi: 10.1186/s13567-026-01847-2 (PMC13647676; doi:10.1186/s13567-026-01847-2)
Supplement: Supplementary file 1 [file 13567_2026_1847_MOESM3_ESM.pdf]

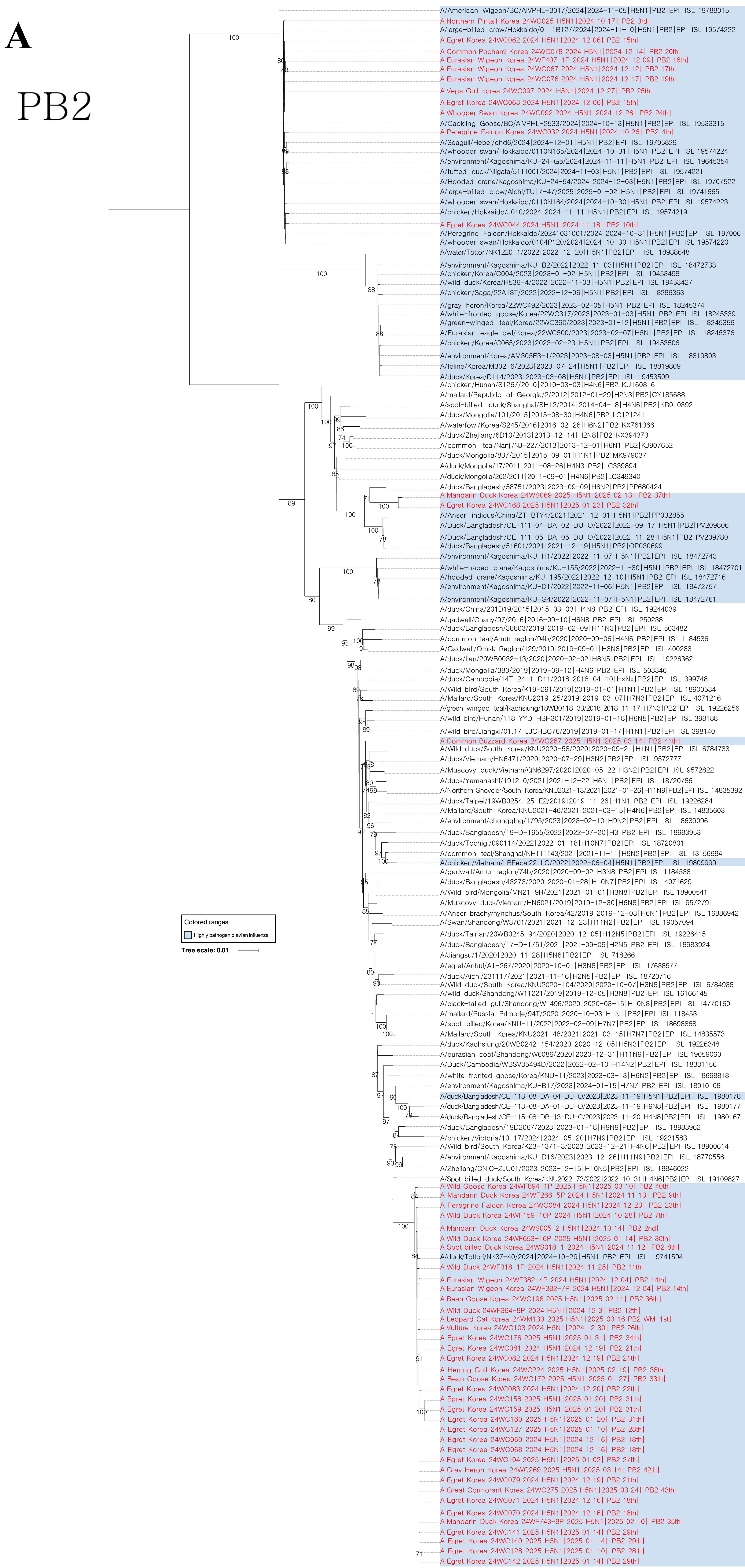
A
PB2
A/American Wigeon/BC/AIVPHL-3017/2024|2024-11-05|H5N1|PB2|EPI ISL 19788015
A Northern Pintail Korea 24WC025 H5N1|2024 10 17| PB2 3rd|
A/large-billed crow/Hokkaido/0111B127/2024|2024-11-10|H5N1|PB2|EPI ISL 19574222
A Egret Korea 24WC062 2024 H5N1|2024 12 06| PB2 15th|
A Common Pochard Korea 24WC078 2024 H5N1|2024 12 14| PB2 20th|
A Eurasian Wigeon Korea 24WF407-1P 2024 H5N1|2024 12 09| PB2 16th|
A Eurasian Wigeon Korea 24WC067 2024 H5N1|2024 12 12| PB2 17th|
A Eurasian Wigeon Korea 24WC076 2024 H5N1|2024 12 17| PB2 19th|
A Vega Gull Korea 24WC097 2024 H5N1|2024 12 27| PB2 25th|
A Egret Korea 24WC063 2024 H5N1|2024 12 06| PB2 15th|
A Whooper Swan Korea 24WC092 2024 H5N1|2024 12 26| PB2 24th|
A/Cackling Goose/BC/AIVPHL-2533/2024|2024-10-13|H5N1|PB2|EPI ISL 19533315
A Peregrine Falcon Korea 24WC032 2024 H5N1|2024 10 26| PB2 4th|
A/Seagull/Hebei/qhd6/2024|2024-12-01|H5N1|PB2|EPI ISL 19795829
A/whooper swan/Hokkaido/0110N165/2024|2024-10-31|H5N1|PB2|EPI ISL 19574224
A/environment/Kagoshima/KU-24-G5/2024|2024-11-11|H5N1|PB2|EPI ISL 19645354
A/tufted duck/Niigata/5111001/2024|2024-11-03|H5N1|PB2|EPI ISL 19574221
A/Hooded crane/Kagoshima/KU-24-54/2024|2024-12-03|H5N1|PB2|EPI ISL 19707522
A/large-billed crow/Aichi/TU17-47/2025|2025-01-02|H5N1|PB2|EPI ISL 19741665
A/whooper swan/Hokkaido/0110N164/2024|2024-10-30|H5N1|PB2|EPI ISL 19574223
A/chicken/Hokkaido/J010/2024|2024-11-11|H5N1|PB2|EPI ISL 19574219
A Egret Korea 24WC044 2024 H5N1|2024 11 18| PB2 10th|
A/Peregrine Falcon/Hokkaido/20241031001/2024|2024-10-31|H5N1|PB2|EPI ISL 197006
A/whooper swan/Hokkaido/0104P120/2024|2024-10-30|H5N1|PB2|EPI ISL 19574220
A/water/Tottori/NK1220-1/2022|2022-12-20|H5N1|PB2|EPI ISL 18938648
A/environment/Kagoshima/KU-B2/2022|2022-11-03|H5N1|PB2|EPI ISL 18472733
A/chicken/Korea/C004/2023|2023-01-02|H5N1|PB2|EPI ISL 19453498
A/wild duck/Korea/H536-4/2022|2022-11-03|H5N1|PB2|EPI ISL 19453427
A/chicken/Saga/22A18T/2022|2022-12-06|H5N1|PB2|EPI ISL 18286363
A/gray heron/Korea/22WC492/2023|2023-02-05|H5N1|PB2|EPI ISL 18245374
A/white-fronted goose/Korea/22WC317/2023|2023-01-03|H5N1|PB2|EPI ISL 18245339
A/green-winged teal/Korea/22WC390/2023|2023-01-12|H5N1|PB2|EPI ISL 18245356
A/Eurasian eagle owl/Korea/22WC500/2023|2023-02-07|H5N1|PB2|EPI ISL 18245376
A/chicken/Korea/C065/2023|2023-02-23|H5N1|PB2|EPI ISL 19453506
A/environment/Korea/AM305E3-1/2023|2023-08-03|H5N1|PB2|EPI ISL 18819803
A/feline/Korea/M302-6/2023|2023-07-24|H5N1|PB2|EPI ISL 18819809
A/duck/Korea/D114/2023|2023-03-08|H5N1|PB2|EPI ISL 19453509
A/chicken/Hunan/S1267/2010|2010-03-03|H4N6|PB2|KU160816
A/mallard/Republic of Georgia/2/2012|2012-01-29|H2N3|PB2|CY185688
A/spot-billed duck/Shanghai/SH12/2014|2014-04-18|H4N6|PB2|KR010392
A/duck/Mongolia/101/2015|2015-08-30|H4N6|PB2|LC121241
A/waterfowl/Korea/S245/2016|2016-02-26|H6N2|PB2|KX761366
A/duck/Zhejiang/6D10/2013|2013-12-14|H2N8|PB2|KX394373
A/common teal/Nanji/NJ-227/2013|2013-12-01|H6N1|PB2|KJ907652
A/duck/Mongolia/837/2015|2015-09-01|H1N1|PB2|MK979037
A/duck/Mongolia/17/2011|2011-08-26|H4N3|PB2|LC339894
A/duck/Mongolia/262/2011|2011-09-01|H4N6|PB2|LC349340
A/duck/Bangladesh/58751/2023|2023-09-09|H6N2|PB2|PP680424
A Mandarin Duck Korea 24WS069 2025 H5N1|2025 02 13| PB2 37th|
A Egret Korea 24WC168 2025 H5N1|2025 01 23| PB2 32th|
A/Anser indicus/China/ZT-BTY4/2021|2021-12-01|H5N1|PB2|PP032855
A/Duck/Bangladesh/CE-111-04-DA-02-DU-O/2022|2022-09-17|H5N1|PB2|PV209806
A/Duck/Bangladesh/CE-111-05-DA-05-DU-O/2022|2022-11-28|H5N1|PB2|PV209780
A/duck/Bangladesh/51601/2021|2021-12-19|H5N1|PB2|OP030699
A/environment/Kagoshima/KU-H1/2022|2022-11-07|H5N1|PB2|EPI ISL 18472743
A/white-naped crane/Kagoshima/KU-155/2022|2022-11-30|H5N1|PB2|EPI ISL 18472701
A/hooded crane/Kagoshima/KU-195/2022|2022-12-10|H5N1|PB2|EPI ISL 18472716
A/environment/Kagoshima/KU-D1/2022|2022-11-06|H5N1|PB2|EPI ISL 18472757
A/environment/Kagoshima/KU-G4/2022|2022-11-07|H5N1|PB2|EPI ISL 18472761
A/duck/China/201D19/2015|2015-03-03|H4N8|PB2|EPI ISL 19244039
A/gadwall/Chany/97/2016|2016-09-10|H6N8|PB2|EPI ISL 250238
A/duck/Bangladesh/38803/2019|2019-02-09|H11N3|PB2|EPI ISL 503482
A/common teal/Amur region/94b/2020|2020-09-06|H4N6|PB2|EPI ISL 1184536
A/Gadwall/Omsk Region/129/2019|2019-09-01|H3N8|PB2|EPI ISL 400283
A/duck/Ilan/20WB0032-13/2020|2020-02-02|H8N5|PB2|EPI ISL 19226362
A/duck/Mongolia/380/2019|2019-09-12|H4N6|PB2|EPI ISL 503346
A/duck/Cambodia/14T-24-1-D11/2018|2018-04-10|HxNx|PB2|EPI ISL 399748
A/Wild bird/South Korea/K19-291/2019|2019-01-01|H1N1|PB2|EPI ISL 18900534
A/Mallard/South Korea/KNU2019-25/2019|2019-03-07|H7N3|PB2|EPI ISL 4071216
A/green-winged teal/Kaohsiung/18WB0118-33/2018|2018-11-17|H7N3|PB2|EPI ISL 19226256
A/wild bird/Hunan/118 YYDTHBH301/2019|2019-01-18|H6N5|PB2|EPI ISL 398188
A/wild bird/Jiangxi/01.17 JJCHBC76/2019|2019-01-17|H1N1|PB2|EPI ISL 398140
A Common Buzzard Korea 24WC267 2025 H5N1|2025 03 14| PB2 41th|
A/Wild duck/South Korea/KNU2020-58/2020|2020-09-21|H1N1|PB2|EPI ISL 6784733
A/duck/Vietnam/HN6471/2020|2020-07-29|H3N2|PB2|EPI ISL 9572777
A/Muscovy duck/Vietnam/QN6297/2020|2020-05-22|H3N2|PB2|EPI ISL 9572822
A/duck/Yamanashi/191210/2021|2021-12-22|H6N1|PB2|EPI ISL 18720786
A/Northern Shoveler/South Korea/KNU2021-13/2021|2021-01-26|H11N9|PB2|EPI ISL 14835392
A/duck/Taipei/19WB0254-25-E2/2019|2019-11-26|H1N1|PB2|EPI ISL 19226284
A/Mallard/South Korea/KNU2021-46/2021|2021-03-15|H4N6|PB2|EPI ISL 14835603
A/environment/chongqing/1795/2023|2023-02-10|H9N2|PB2|EPI ISL 18639096
A/duck/Bangladesh/19-D-1955/2022|2022-07-20|H3|PB2|EPI ISL 18983953
A/duck/Tochigi/090114/2022|2022-01-18|H10N7|PB2|EPI ISL 18720801
A/common teal/Shanghai/NH111143/2021|2021-11-11|H9N2|PB2|EPI ISL 13156684
A/chicken/Vietnam/LBFecal221LC/2022|2022-06-04|H5N1|PB2|EPI ISL 19809999
A/gadwall/Amur region/74b/2020|2020-09-02|H3N8|PB2|EPI ISL 1184538
A/duck/Bangladesh/43273/2020|2020-01-28|H10N7|PB2|EPI ISL 4071629
A/Wild bird/Mongolia/MN21-9R/2021|2021-01-01|H3N8|PB2|EPI ISL 18900541
A/Muscovy duck/Vietnam/HN6021/2019|2019-12-30|H6N8|PB2|EPI ISL 9572791
A/Anser brachyrhynchus/South Korea/42/2019|2019-12-03|H6N1|PB2|EPI ISL 16886942
A/Swan/Shandong/W3701/2021|2021-12-23|H11N2|PB2|EPI ISL 19057094
A/duck/Tainan/20WB0245-94/2020|2020-12-05|H12N5|PB2|EPI ISL 19226415
A/duck/Bangladesh/17-D-1751/2021|2021-09-09|H2N5|PB2|EPI ISL 18983924
A/Jiangsu/1/2020|2020-11-28|H5N6|PB2|EPI ISL 718266
A/egret/Anhui/A1-267/2020|2020-10-01|H3N8|PB2|EPI ISL 17638577
A/duck/Aichi/231117/2021|2021-11-16|H2N5|PB2|EPI ISL 18720716
A/Wild duck/South Korea/KNU2020-104/2020|2020-10-07|H3N8|PB2|EPI ISL 6784938
A/wild duck/Shandong/W11221/2019|2019-12-05|H3N8|PB2|EPI ISL 16166145
A/black-tailed gull/Shandong/W1496/2020|2020-03-15|H10N8|PB2|EPI ISL 14770160
A/mallard/Russia Primorje/94T/2020|2020-10-03|H1N1|PB2|EPI ISL 1184531
A/spot billed/Korea/KNU-11/2022|2022-02-09|H7N7|PB2|EPI ISL 18698868
A/Mallard/South Korea/KNU2021-48/2021|2021-03-15|H7N7|PB2|EPI ISL 14835573
A/duck/Kaohsiung/20WB0242-154/2020|2020-12-05|H5N3|PB2|EPI ISL 19226348
A/eurasian coot/Shandong/W6086/2020|2020-12-31|H11N9|PB2|EPI ISL 19059060
A/Duck/Cambodia/WBSV35494D/2022|2022-02-10|H14N2|PB2|EPI ISL 18331156
A/white fronted goose/Korea/KNU-11/2023|2023-03-13|H6N2|PB2|EPI ISL 18698818
A/environment/Kagoshima/KU-B17/2023|2024-01-15|H7N7|PB2|EPI ISL 18910108
A/duck/Bangladesh/CE-113-08-DA-04-DU-O/2023|2023-11-19|H5N1|PB2|EPI ISL 1980178
A/duck/Bangladesh/CE-113-08-DA-01-DU-O/2023|2023-11-19|H9N8|PB2|EPI ISL 1980177
A/duck/Bangladesh/CE-115-08-DB-13-DU-C/2023|2023-11-20|H4N8|PB2|EPI ISL 1980167
A/duck/Bangladesh/19D2067/2023|2023-01-18|H9N9|PB2|EPI ISL 18983962
A/chicken/Victoria/10-17/2024|2024-05-20|H7N9|PB2|EPI ISL 19231583
A/Wild bird/South Korea/K23-1371-3/2023|2023-12-21|H4N6|PB2|EPI ISL 18900614
A/environment/Kagoshima/KU-D16/2023|2023-12-26|H11N9|PB2|EPI ISL 18770556
A/Zhejiang/CNIC-ZJU01/2023|2023-12-15|H10N5|PB2|EPI ISL 18846022
A/Spot-billed duck/South Korea/KNU2022-73/2022|2022-10-31|H4N6|PB2|EPI ISL 19109827
A Wild Goose Korea 24WF894-1P 2025 H5N1|2025 03 10| PB2 40th|
A Mandarin Duck Korea 24WF266-5P 2024 H5N1|2024 11 13| PB2 9th|
A Peregrine Falcon Korea 24WC084 2024 H5N1|2024 12 23| PB2 23th|
A Wild Duck Korea 24WF159-10P 2024 H5N1|2024 10 28| PB2 7th|
A Mandarin Duck Korea 24WS005-2 H5N1|2024 10 14| PB2 2nd|
A Wild Duck Korea 24WF653-16P 2025 H5N1|2025 01 14| PB2 30th|
A Spot billed Duck Korea 24WS018-1 2024 H5N1|2024 11 12| PB2 8th|
A/duck/Tottori/NK37-40/2024|2024-10-29|H5N1|PB2|EPI ISL 19741594
A Wild Duck 24WF318-1P 2024 H5N1|2024 11 25| PB2 11th|
A Eurasian Wigeon Korea 24WF382-4P 2024 H5N1|2024 12 04| PB2 14th|
A Eurasian Wigeon Korea 24WF382-7P 2024 H5N1|2024 12 04| PB2 14th|
A Bean Goose Korea 24WC196 2025 H5N1|2025 02 11| PB2 36th|
A Wild Duck 24WF364-8P 2024 H5N1|2024 12 3| PB2 12th|
A Leopard Cat Korea 24WM130 2025 H5N1|2025 03 16 PB2 WM-1st|
A Vulture Korea 24WC103 2024 H5N1|2024 12 30| PB2 26th|
A Egret Korea 24WC176 2025 H5N1|2025 01 31| PB2 34th|
A Egret Korea 24WC081 2024 H5N1|2024 12 19| PB2 21th|
A Egret Korea 24WC082 2024 H5N1|2024 12 19| PB2 21th|
A Herring Gull Korea 24WC224 2025 H5N1|2025 02 19| PB2 38th|
A Bean Goose Korea 24WC172 2025 H5N1|2025 01 27| PB2 33th|
A Egret Korea 24WC083 2024 H5N1|2024 12 20| PB2 22th|
A Egret Korea 24WC158 2025 H5N1|2025 01 20| PB2 31th|
A Egret Korea 24WC159 2025 H5N1|2025 01 20| PB2 31th|
A Egret Korea 24WC160 2025 H5N1|2025 01 20| PB2 31th|
A Egret Korea 24WC127 2025 H5N1|2025 01 10| PB2 28th|
A Egret Korea 24WC069 2024 H5N1|2024 12 16| PB2 18th|
A Egret Korea 24WC068 2024 H5N1|2024 12 16| PB2 18th|
A Egret Korea 24WC104 2025 H5N1|2025 01 02| PB2 27th|
A Gray Heron Korea 24WC269 2025 H5N1|2025 03 14| PB2 42th|
A Egret Korea 24WC079 2024 H5N1|2024 12 19| PB2 21th|
A Great Cormorant Korea 24WC275 2025 H5N1|2025 03 24| PB2 43th|
A Egret Korea 24WC071 2024 H5N1|2024 12 16| PB2 18th|
A Egret Korea 24WC070 2024 H5N1|2024 12 16| PB2 18th|
A Mandarin Duck Korea 24WF743-8P 2025 H5N1|2025 02 10| PB2 35th|
A Egret Korea 24WC141 2025 H5N1|2025 01 14| PB2 29th|
A Egret Korea 24WC140 2025 H5N1|2025 01 14| PB2 29th|
A Egret Korea 24WC128 2025 H5N1|2025 01 10| PB2 28th|
A Egret Korea 24WC142 2025 H5N1|2025 01 14| PB2 29th|
Colored ranges
Highly pathogenic avian influenza
Tree scale: 0.01

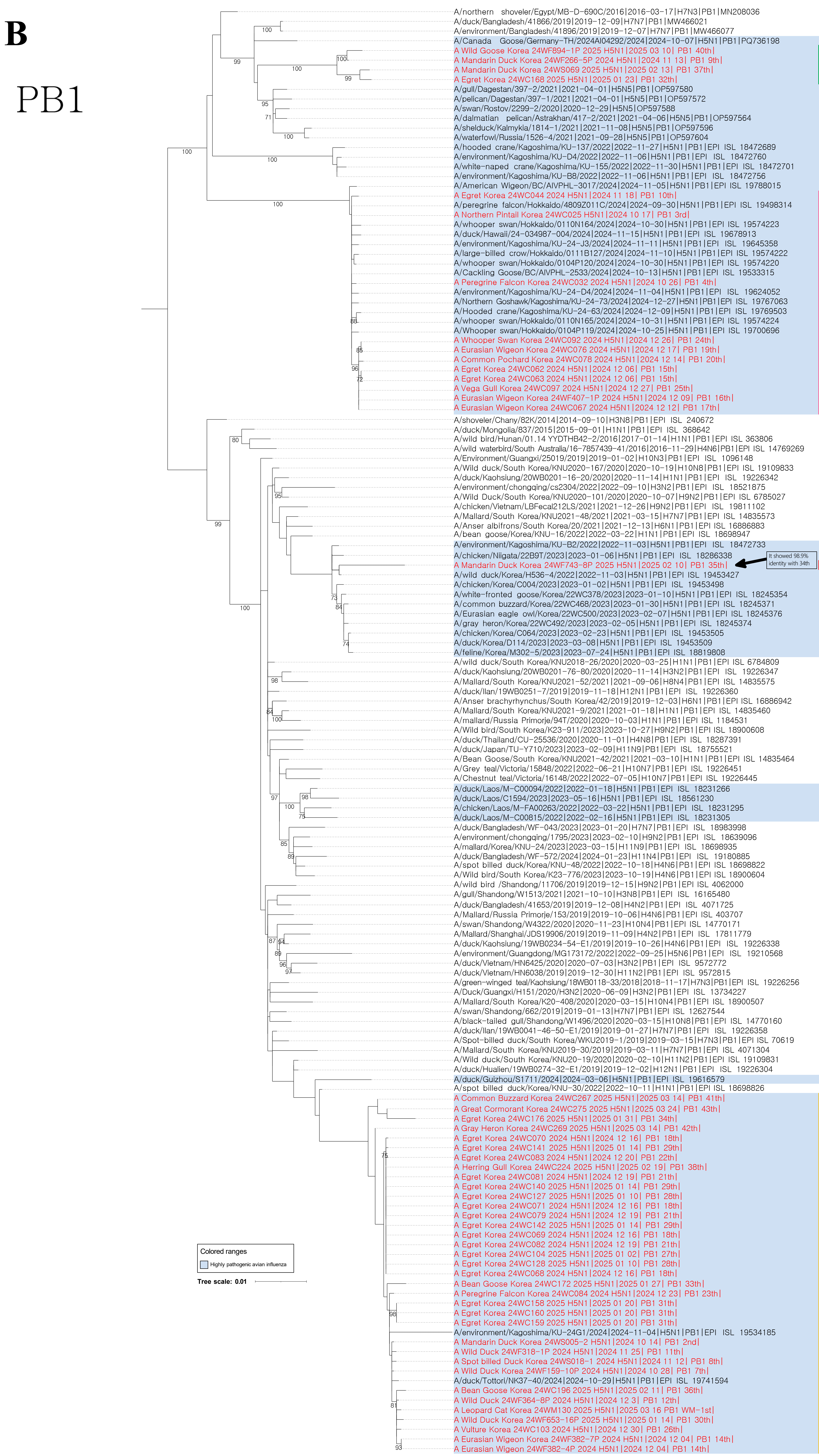
B
PB1
A/northern shoveler/Egypt/MB-D-690C/2016|2016-03-17|H7N3|PB1|MN208036
A/duck/Bangladesh/41866/2019|2019-12-09|H7N7|PB1|MW466021
A/environment/Bangladesh/41896/2019|2019-12-07|H7N7|PB1|MW466077
A/Canada Goose/Germany-TH/2024AI04292/2024|2024-10-07|H5N1|PB1|PQ736198
A Wild Goose Korea 24WF894-1P 2025 H5N1|2025 03 10| PB1 40th|
A Mandarin Duck Korea 24WF266-5P 2024 H5N1|2024 11 13| PB1 9th|
A Mandarin Duck Korea 24WS069 2025 H5N1|2025 02 13| PB1 37th|
A Egret Korea 24WC168 2025 H5N1|2025 01 23| PB1 32th|
A/gull/Dagestan/397-2/2021|2021-04-01|H5N5|PB1|OP597580
A/pelican/Dagestan/397-1/2021|2021-04-01|H5N5|PB1|OP597572
A/swan/Rostov/2299-2/2020|2020-12-29|H5N5|OP597588
A/dalmatian pelican/Astrakhan/417-2/2021|2021-04-06|H5N5|PB1|OP597564
A/shelduck/Kalmykia/1814-1/2021|2021-11-08|H5N5|PB1|OP597596
A/waterfowl/Russia/1526-4/2021|2021-09-28|H5N5|PB1|OP597604
A/hooded crane/Kagoshima/KU-137/2022|2022-11-27|H5N1|PB1|EPI ISL 18472689
A/environment/Kagoshima/KU-D4/2022|2022-11-06|H5N1|PB1|EPI ISL 18472760
A/white-naped crane/Kagoshima/KU-155/2022|2022-11-30|H5N1|PB1|EPI ISL 18472701
A/environment/Kagoshima/KU-B8/2022|2022-11-06|H5N1|PB1|EPI ISL 18472756
A/American Wigeon/BC/AIVPHL-3017/2024|2024-11-05|H5N1|PB1|EPI ISL 19788015
A Egret Korea 24WC044 2024 H5N1|2024 11 18| PB1 10th|
A/peregrine falcon/Hokkaido/4809Z011C/2024|2024-09-30|H5N1|PB1|EPI ISL 19498314
A Northern Pintail Korea 24WC025 H5N1|2024 10 17| PB1 3rd|
A/whooper swan/Hokkaido/0110N164/2024|2024-10-30|H5N1|PB1|EPI ISL 19574223
A/duck/Hawaii/24-034987-004/2024|2024-11-15|H5N1|PB1|EPI ISL 19678913
A/environment/Kagoshima/KU-24-J3/2024|2024-11-11|H5N1|PB1|EPI ISL 19645358
A/large-billed crow/Hokkaido/0111B127/2024|2024-11-10|H5N1|PB1|EPI ISL 19574222
A/whooper swan/Hokkaido/0104P120/2024|2024-10-30|H5N1|PB1|EPI ISL 19574220
A/Cackling Goose/BC/AIVPHL-2533/2024|2024-10-13|H5N1|PB1|EPI ISL 19533315
A Peregrine Falcon Korea 24WC032 2024 H5N1|2024 10 26| PB1 4th|
A/environment/Kagoshima/KU-24-D4/2024|2024-11-04|H5N1|PB1|EPI ISL 19624052
A/Northern Goshawk/Kagoshima/KU-24-73/2024|2024-12-27|H5N1|PB1|EPI ISL 19767063
A/Hooded crane/Kagoshima/KU-24-63/2024|2024-12-09|H5N1|PB1|EPI ISL 19769503
A/whooper swan/Hokkaido/0110N165/2024|2024-10-31|H5N1|PB1|EPI ISL 19574224
A/Whooper swan/Hokkaido/0104P119/2024|2024-10-25|H5N1|PB1|EPI ISL 19700696
A Whooper Swan Korea 24WC092 2024 H5N1|2024 12 26| PB1 24th|
A Eurasian Wigeon Korea 24WC076 2024 H5N1|2024 12 17| PB1 19th|
A Common Pochard Korea 24WC078 2024 H5N1|2024 12 14| PB1 20th|
A Egret Korea 24WC062 2024 H5N1|2024 12 06| PB1 15th|
A Egret Korea 24WC063 2024 H5N1|2024 12 06| PB1 15th|
A Vega Gull Korea 24WC097 2024 H5N1|2024 12 27| PB1 25th|
A Eurasian Wigeon Korea 24WF407-1P 2024 H5N1|2024 12 09| PB1 16th|
A Eurasian Wigeon Korea 24WC067 2024 H5N1|2024 12 12| PB1 17th|
A/shoveler/Chany/82K/2014|2014-09-10|H3N8|PB1|EPI ISL 240672
A/duck/Mongolia/837/2015|2015-09-01|H1N1|PB1|EPI ISL 368642
A/wild bird/Hunan/01.14 YYDTHB42-2/2016|2017-01-14|H1N1|PB1|EPI ISL 363806
A/wild waterbird/South Australia/16-7857439-41/2016|2016-11-29|H4N6|PB1|EPI ISL 14769269
A/Environment/Guangxi/25019/2019|2019-01-02|H10N3|PB1|EPI ISL 1096148
A/Wild duck/South Korea/KNU2020-167/2020|2020-10-19|H10N8|PB1|EPI ISL 19109833
A/duck/Kaohsiung/20WB0201-16-20/2020|2020-11-14|H1N1|PB1|EPI ISL 19226342
A/environment/chongqing/cs2304/2022|2022-09-10|H3N2|PB1|EPI ISL 18521875
A/Wild Duck/South Korea/KNU2020-101/2020|2020-10-07|H9N2|PB1|EPI ISL 6785027
A/chicken/Vietnam/LBFecal212LS/2021|2021-12-26|H9N2|PB1|EPI ISL 19811102
A/Mallard/South Korea/KNU2021-48/2021|2021-03-15|H7N7|PB1|EPI ISL 14835573
A/Anser albifrons/South Korea/20/2021|2021-12-13|H6N1|PB1|EPI ISL 16886883
A/bean goose/Korea/KNU-16/2022|2022-03-22|H1N1|PB1|EPI ISL 18698947
A/environment/Kagoshima/KU-B2/2022|2022-11-03|H5N1|PB1|EPI ISL 18472733
A/chicken/Niigata/22B9T/2023|2023-01-06|H5N1|PB1|EPI ISL 18286338
A Mandarin Duck Korea 24WF743-8P 2025 H5N1|2025 02 10| PB1 35th|
It showed 98.9% identity with 34th
A/wild duck/Korea/H536-4/2022|2022-11-03|H5N1|PB1|EPI ISL 19453427
A/chicken/Korea/C004/2023|2023-01-02|H5N1|PB1|EPI ISL 19453498
A/white-fronted goose/Korea/22WC378/2023|2023-01-10|H5N1|PB1|EPI ISL 18245354
A/common buzzard/Korea/22WC468/2023|2023-01-30|H5N1|PB1|EPI ISL 18245371
A/Eurasian eagle owl/Korea/22WC500/2023|2023-02-07|H5N1|PB1|EPI ISL 18245376
A/gray heron/Korea/22WC492/2023|2023-02-05|H5N1|PB1|EPI ISL 18245374
A/chicken/Korea/C064/2023|2023-02-23|H5N1|PB1|EPI ISL 19453505
A/duck/Korea/D114/2023|2023-03-08|H5N1|PB1|EPI ISL 19453509
A/feline/Korea/M302-5/2023|2023-07-24|H5N1|PB1|EPI ISL 18819808
A/wild duck/South Korea/KNU2018-26/2020|2020-03-25|H1N1|PB1|EPI ISL 6784809
A/duck/Kaohsiung/20WB0201-76-80/2020|2020-11-14|H3N2|PB1|EPI ISL 19226347
A/Mallard/South Korea/KNU2021-52/2021|2021-09-06|H8N4|PB1|EPI ISL 14835575
A/duck/Ilan/19WB0251-7/2019|2019-11-18|H12N1|PB1|EPI ISL 19226360
A/Anser brachyrhynchus/South Korea/42/2019|2019-12-03|H6N1|PB1|EPI ISL 16886942
A/Mallard/South Korea/KNU2021-9/2021|2021-01-18|H1N1|PB1|EPI ISL 14835460
A/mallard/Russia Primorje/94T/2020|2020-10-03|H1N1|PB1|EPI ISL 1184531
A/Wild bird/South Korea/K23-911/2023|2023-10-27|H9N2|PB1|EPI ISL 18900608
A/duck/Thailand/CU-25536/2020|2020-11-01|H4N8|PB1|EPI ISL 18287391
A/duck/Japan/TU-Y710/2023|2023-02-09|H11N9|PB1|EPI ISL 18755521
A/Bean Goose/South Korea/KNU2021-42/2021|2021-03-10|H1N1|PB1|EPI ISL 14835464
A/Grey teal/Victoria/15848/2022|2022-06-21|H10N7|PB1|EPI ISL 19226451
A/Chestnut teal/Victoria/16148/2022|2022-07-05|H10N7|PB1|EPI ISL 19226445
A/duck/Laos/M-C00094/2022|2022-01-18|H5N1|PB1|EPI ISL 18231266
A/duck/Laos/C1594/2023|2023-05-16|H5N1|PB1|EPI ISL 18561230
A/chicken/Laos/M-FA00263/2022|2022-03-22|H5N1|PB1|EPI ISL 18231295
A/duck/Laos/M-C00815/2022|2022-02-16|H5N1|PB1|EPI ISL 18231305
A/duck/Bangladesh/WF-043/2023|2023-01-20|H7N7|PB1|EPI ISL 18983998
A/environment/chongqing/1795/2023|2023-02-10|H9N2|PB1|EPI ISL 18639096
A/mallard/Korea/KNU-24/2023|2023-03-15|H11N9|PB1|EPI ISL 18698935
A/duck/Bangladesh/WF-572/2024|2024-01-23|H11N4|PB1|EPI ISL 19180885
A/spot billed duck/Korea/KNU-48/2022|2022-10-18|H4N6|PB1|EPI ISL 18698822
A/Wild bird/South Korea/K23-776/2023|2023-10-19|H4N6|PB1|EPI ISL 18900604
A/wild bird /Shandong/11706/2019|2019-12-15|H9N2|PB1|EPI ISL 4062000
A/gull/Shandong/W1513/2021|2021-10-10|H3N8|PB1|EPI ISL 16165480
A/duck/Bangladesh/41653/2019|2019-12-08|H4N2|PB1|EPI ISL 4071725
A/Mallard/Russia Primorje/153/2019|2019-10-06|H4N6|PB1|EPI ISL 403707
A/swan/Shandong/W4322/2020|2020-11-23|H10N4|PB1|EPI ISL 14770171
A/Mallard/Shanghai/JDS19906/2019|2019-11-09|H4N2|PB1|EPI ISL 17811779
A/duck/Kaohsiung/19WB0234-54-E1/2019|2019-10-26|H4N6|PB1|EPI ISL 19226338
A/environment/Guangdong/MG173172/2022|2022-09-25|H5N6|PB1|EPI ISL 19210568
A/duck/Vietnam/HN6425/2020|2020-07-03|H3N2|PB1|EPI ISL 9572772
A/duck/Vietnam/HN6038/2019|2019-12-30|H11N2|PB1|EPI ISL 9572815
A/green-winged teal/Kaohsiung/18WB0118-33/2018|2018-11-17|H7N3|PB1|EPI ISL 19226256
A/Duck/Guangxi/H151/2020/H3N2|2020-06-09|H3N2|PB1|EPI ISL 13734227
A/Mallard/South Korea/K20-408/2020|2020-03-15|H10N4|PB1|EPI ISL 18900507
A/swan/Shandong/662/2019|2019-01-13|H7N7|PB1|EPI ISL 12627544
A/black-tailed gull/Shandong/W1496/2020|2020-03-15|H10N8|PB1|EPI ISL 14770160
A/duck/Ilan/19WB0041-46-50-E1/2019|2019-01-27|H7N7|PB1|EPI ISL 19226358
A/Spot-billed duck/South Korea/WKU2019-1/2019|2019-03-15|H7N3|PB1|EPI ISL 70619
A/Mallard/South Korea/KNU2019-30/2019|2019-03-11|H7N7|PB1|EPI ISL 4071304
A/Wild duck/South Korea/KNU20-19/2020|2020-02-10|H11N2|PB1|EPI ISL 19109831
A/duck/Hualien/19WB0274-32-E1/2019|2019-12-02|H12N1|PB1|EPI ISL 19226304
A/duck/Guizhou/S1711/2024|2024-03-06|H5N1|PB1|EPI ISL 19616579
A/spot billed duck/Korea/KNU-30/2022|2022-10-11|H1N1|PB1|EPI ISL 18698826
A Common Buzzard Korea 24WC267 2025 H5N1|2025 03 14| PB1 41th|
A Great Cormorant Korea 24WC275 2025 H5N1|2025 03 24| PB1 43th|
A Egret Korea 24WC176 2025 H5N1|2025 01 31| PB1 34th|
A Gray Heron Korea 24WC269 2025 H5N1|2025 03 14| PB1 42th|
A Egret Korea 24WC070 2024 H5N1|2024 12 16| PB1 18th|
A Egret Korea 24WC141 2025 H5N1|2025 01 14| PB1 29th|
A Egret Korea 24WC083 2024 H5N1|2024 12 20| PB1 22th|
A Herring Gull Korea 24WC224 2025 H5N1|2025 02 19| PB1 38th|
A Egret Korea 24WC081 2024 H5N1|2024 12 19| PB1 21th|
A Egret Korea 24WC140 2025 H5N1|2025 01 14| PB1 29th|
A Egret Korea 24WC127 2025 H5N1|2025 01 10| PB1 28th|
A Egret Korea 24WC071 2024 H5N1|2024 12 16| PB1 18th|
A Egret Korea 24WC079 2024 H5N1|2024 12 19| PB1 21th|
A Egret Korea 24WC142 2025 H5N1|2025 01 14| PB1 29th|
A Egret Korea 24WC069 2024 H5N1|2024 12 16| PB1 18th|
A Egret Korea 24WC082 2024 H5N1|2024 12 19| PB1 21th|
A Egret Korea 24WC104 2025 H5N1|2025 01 02| PB1 27th|
A Egret Korea 24WC128 2025 H5N1|2025 01 10| PB1 28th|
A Egret Korea 24WC068 2024 H5N1|2024 12 16| PB1 18th|
A Bean Goose Korea 24WC172 2025 H5N1|2025 01 27| PB1 33th|
A Peregrine Falcon Korea 24WC084 2024 H5N1|2024 12 23| PB1 23th|
A Egret Korea 24WC158 2025 H5N1|2025 01 20| PB1 31th|
A Egret Korea 24WC160 2025 H5N1|2025 01 20| PB1 31th|
A Egret Korea 24WC159 2025 H5N1|2025 01 20| PB1 31th|
A/environment/Kagoshima/KU-24G1/2024|2024-11-04|H5N1|PB1|EPI ISL 19534185
A Mandarin Duck Korea 24WS005-2 H5N1|2024 10 14| PB1 2nd|
A Wild Duck 24WF318-1P 2024 H5N1|2024 11 25| PB1 11th|
A Spot billed Duck Korea 24WS018-1 2024 H5N1|2024 11 12| PB1 8th|
A Wild Duck Korea 24WF159-10P 2024 H5N1|2024 10 28| PB1 7th|
A/duck/Tottori/NK37-40/2024|2024-10-29|H5N1|PB1|EPI ISL 19741594
A Bean Goose Korea 24WC196 2025 H5N1|2025 02 11| PB1 36th|
A Wild Duck 24WF364-8P 2024 H5N1|2024 12 3| PB1 12th|
A Leopard Cat Korea 24WM130 2025 H5N1|2025 03 16 PB1 WM-1st|
A Wild Duck Korea 24WF653-16P 2025 H5N1|2025 01 14| PB1 30th|
A Vulture Korea 24WC103 2024 H5N1|2024 12 30| PB1 26th|
A Eurasian Wigeon Korea 24WF382-7P 2024 H5N1|2024 12 04| PB1 14th|
A Eurasian Wigeon 24WF382-4P 2024 H5N1|2024 12 04| PB1 14th|
Colored ranges
Highly pathogenic avian influenza
Tree scale: 0.01

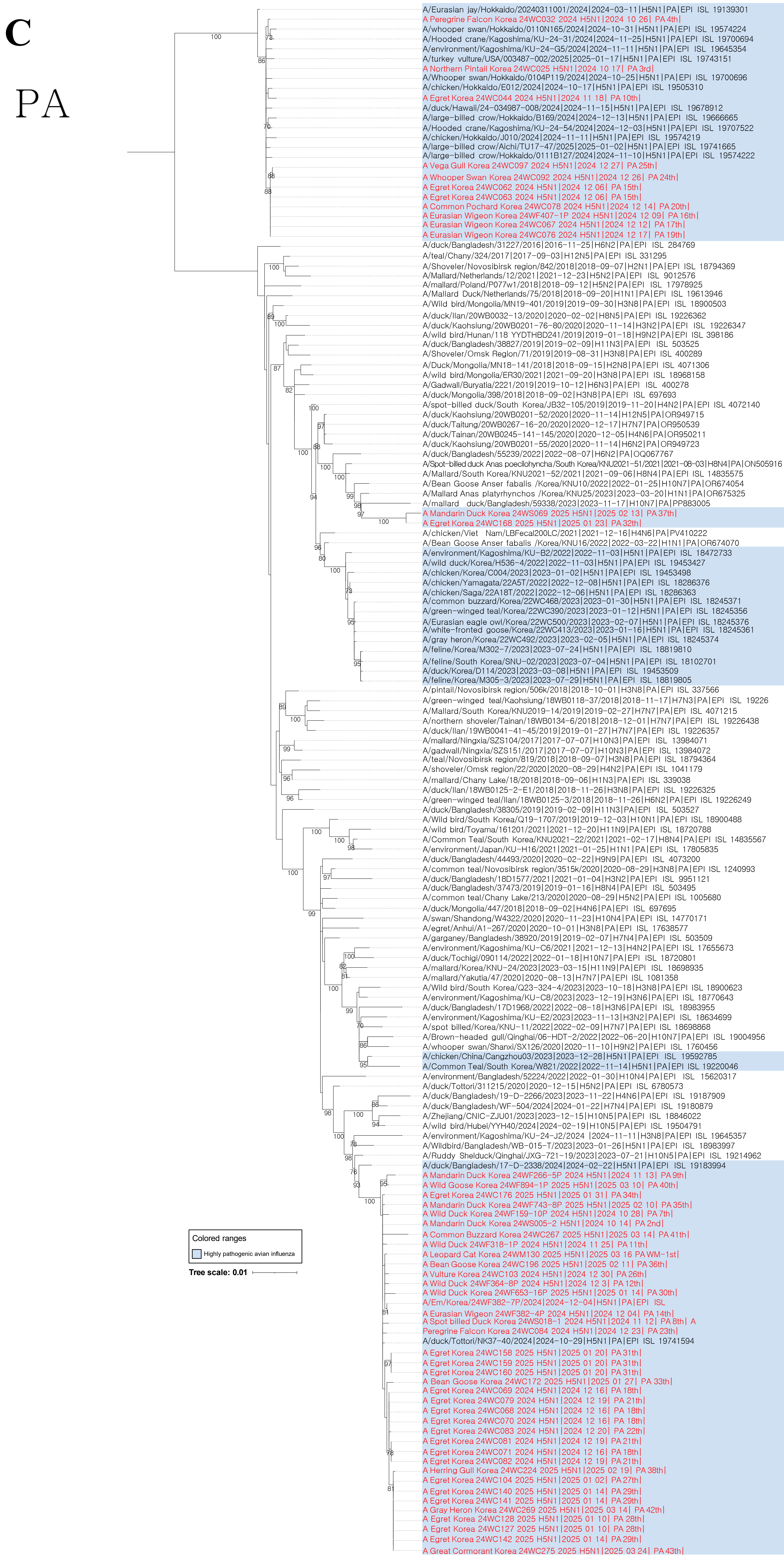
C
PA
A/Eurasian jay/Hokkaido/20240311001/2024|2024-03-11|H5N1|PA|EPI ISL 19139301
A Peregrine Falcon Korea 24WC032 2024 H5N1|2024 10 26| PA 4th|
A/whooper swan/Hokkaido/0110N165/2024|2024-10-31|H5N1|PA|EPI ISL 19574224
A/Hooded crane/Kagoshima/KU-24-31/2024|2024-11-25|H5N1|PA|EPI ISL 19700694
A/environment/Kagoshima/KU-24-G5/2024|2024-11-11|H5N1|PA|EPI ISL 19645354
A/turkey vulture/USA/003487-002/2025|2025-01-17|H5N1|PA|EPI ISL 19743151
A Northern Pintail Korea 24WC025 H5N1|2024 10 17| PA 3rd|
A/Whooper swan/Hokkaido/0104P119/2024|2024-10-25|H5N1|PA|EPI ISL 19700696
A/chicken/Hokkaido/E012/2024|2024-10-17|H5N1|PA|EPI ISL 19505310
A Egret Korea 24WC044 2024 H5N1|2024 11 18| PA 10th|
A/duck/Hawaii/24-034987-008/2024|2024-11-15|H5N1|PA|EPI ISL 19678912
A/large-billed crow/Hokkaido/B169/2024|2024-12-13|H5N1|PA|EPI ISL 19666665
A/Hooded crane/Kagoshima/KU-24-54/2024|2024-12-03|H5N1|PA|EPI ISL 19707522
A/chicken/Hokkaido/J010/2024|2024-11-11|H5N1|PA|EPI ISL 19574219
A/large-billed crow/Aichi/TU17-47/2025|2025-01-02|H5N1|PA|EPI ISL 19741665
A/large-billed crow/Hokkaido/0111B127/2024|2024-11-10|H5N1|PA|EPI ISL 19574222
A Vega Gull Korea 24WC097 2024 H5N1|2024 12 27| PA 25th|
A Whooper Swan Korea 24WC092 2024 H5N1|2024 12 26| PA 24th|
A Egret Korea 24WC062 2024 H5N1|2024 12 06| PA 15th|
A Egret Korea 24WC063 2024 H5N1|2024 12 06| PA 15th|
A Common Pochard Korea 24WC078 2024 H5N1|2024 12 14| PA 20th|
A Eurasian Wigeon Korea 24WF407-1P 2024 H5N1|2024 12 09| PA 16th|
A Eurasian Wigeon Korea 24WC067 2024 H5N1|2024 12 12| PA 17th|
A Eurasian Wigeon Korea 24WC076 2024 H5N1|2024 12 17| PA 19th|
A/duck/Bangladesh/31227/2016|2016-11-25|H6N2|PA|EPI ISL 284769
A/teal/Chany/324/2017|2017-09-03|H12N5|PA|EPI ISL 331295
A/Shoveler/Novosibirsk region/842/2018|2018-09-07|H2N1|PA|EPI ISL 18794369
A/Mallard/Netherlands/12/2021|2021-12-23|H5N2|PA|EPI ISL 9012576
A/mallard/Poland/P077w1/2018|2018-09-12|H5N2|PA|EPI ISL 17978925
A/Mallard Duck/Netherlands/75/2018|2018-09-20|H1N1|PA|EPI ISL 19613946
A/Wild bird/Mongolia/MN19-401/2019|2019-09-30|H3N8|PA|EPI ISL 18900503
A/duck/Ilan/20WB0032-13/2020|2020-02-02|H8N5|PA|EPI ISL 19226362
A/duck/Kaohsiung/20WB0201-76-80/2020|2020-11-14|H3N2|PA|EPI ISL 19226347
A/wild bird/Hunan/118 YYDTHBD241/2019|2019-01-18|H9N2|PA|EPI ISL 398186
A/duck/Bangladesh/38827/2019|2019-02-09|H11N3|PA|EPI ISL 503525
A/Shoveler/Omsk Region/71/2019|2019-08-31|H3N8|PA|EPI ISL 400289
A/Duck/Mongolia/MN18-141/2018|2018-09-15|H2N8|PA|EPI ISL 4071306
A/wild bird/Mongolia/ER30/2021|2021-09-20|H3N8|PA|EPI ISL 18968158
A/Gadwall/Buryatia/2221/2019|2019-10-12|H6N3|PA|EPI ISL 400278
A/duck/Mongolia/398/2018|2018-09-02|H3N8|PA|EPI ISL 697693
A/spot-billed duck/South Korea/JB32-105/2019|2019-11-20|H4N2|PA|EPI ISL 4072140
A/duck/Kaohsiung/20WB0201-52/2020|2020-11-14|H12N5|PA|OR949715
A/duck/Taitung/20WB0267-16-20/2020|2020-12-17|H7N7|PA|OR950539
A/duck/Tainan/20WB0245-141-145/2020|2020-12-05|H4N6|PA|OR950211
A/duck/Kaohsiung/20WB0201-55/2020|2020-11-14|H6N2|PA|OR949723
A/duck/Bangladesh/55239/2022|2022-08-07|H6N2|PA|OQ067767
A/Spot-billed duck Anas poecilohyncha/South Korea/KNU2021-51/2021|2021-08-03|H8N4|PA|ON505916
A/Mallard/South Korea/KNU2021-52/2021|2021-09-06|H8N4|PA|EPI ISL 14835575
A/Bean Goose Anser fabalis /Korea/KNU10/2022|2022-01-25|H10N7|PA|OR674054
A/Mallard Anas platyrhynchos /Korea/KNU25/2023|2023-03-20|H1N1|PA|OR675325
A/mallard duck/Bangladesh/59338/2023|2023-11-17|H10N7|PA|PP883005
A Mandarin Duck Korea 24WS069 2025 H5N1|2025 02 13| PA 37th|
A Egret Korea 24WC168 2025 H5N1|2025 01 23| PA 32th|
A/chicken/Viet Nam/LBFecal200LC/2021|2021-12-16|H4N6|PA|PV410222
A/Bean Goose Anser fabalis /Korea/KNU16/2022|2022-03-22|H1N1|PA|OR674070
A/environment/Kagoshima/KU-B2/2022|2022-11-03|H5N1|PA|EPI ISL 18472733
A/wild duck/Korea/H536-4/2022|2022-11-03|H5N1|PA|EPI ISL 19453427
A/chicken/Korea/C004/2023|2023-01-02|H5N1|PA|EPI ISL 19453498
A/chicken/Yamagata/22A5T/2022|2022-12-08|H5N1|PA|EPI ISL 18286376
A/chicken/Saga/22A18T/2022|2022-12-06|H5N1|PA|EPI ISL 18286363
A/common buzzard/Korea/22WC468/2023|2023-01-30|H5N1|PA|EPI ISL 18245371
A/green-winged teal/Korea/22WC390/2023|2023-01-12|H5N1|PA|EPI ISL 18245356
A/Eurasian eagle owl/Korea/22WC500/2023|2023-02-07|H5N1|PA|EPI ISL 18245376
A/white-fronted goose/Korea/22WC413/2023|2023-01-16|H5N1|PA|EPI ISL 18245361
A/gray heron/Korea/22WC492/2023|2023-02-05|H5N1|PA|EPI ISL 18245374
A/feline/Korea/M302-7/2023|2023-07-24|H5N1|PA|EPI ISL 18819810
A/feline/South Korea/SNU-02/2023|2023-07-04|H5N1|PA|EPI ISL 18102701
A/duck/Korea/D114/2023|2023-03-08|H5N1|PA|EPI ISL 19453509
A/feline/Korea/M305-3/2023|2023-07-29|H5N1|PA|EPI ISL 18819805
A/pintail/Novosibirsk region/506k/2018|2018-10-01|H3N8|PA|EPI ISL 337566
A/green-winged teal/Kaohsiung/18WB0118-37/2018|2018-11-17|H7N3|PA|EPI ISL 19226
A/Mallard/South Korea/KNU2019-14/2019|2019-02-27|H7N7|PA|EPI ISL 4071215
A/northern shoveler/Tainan/18WB0134-6/2018|2018-12-01|H7N7|PA|EPI ISL 19226438
A/duck/Ilan/19WB0041-41-45/2019|2019-01-27|H7N7|PA|EPI ISL 19226357
A/mallard/Ningxia/SZS104/2017|2017-07-07|H10N3|PA|EPI ISL 13984071
A/gadwall/Ningxia/SZS151/2017|2017-07-07|H10N3|PA|EPI ISL 13984072
A/teal/Novosibirsk region/819/2018|2018-09-07|H3N8|PA|EPI ISL 18794364
A/shoveler/Omsk region/22/2020|2020-08-29|H4N2|PA|EPI ISL 1041179
A/mallard/Chany Lake/18/2018|2018-09-06|H1N3|PA|EPI ISL 339038
A/duck/Ilan/18WB0125-2-E1/2018|2018-11-26|H3N8|PA|EPI ISL 19226325
A/green-winged teal/Ilan/18WB0125-3/2018|2018-11-26|H6N2|PA|EPI ISL 19226249
A/duck/Bangladesh/38305/2019|2019-02-09|H11N3|PA|EPI ISL 503527
A/Wild bird/South Korea/Q19-1707/2019|2019-12-03|H10N1|PA|EPI ISL 18900488
A/wild bird/Toyama/161201/2021|2021-12-20|H11N9|PA|EPI ISL 18720788
A/Common Teal/South Korea/KNU2021-22/2021|2021-02-17|H8N4|PA|EPI ISL 14835567
A/environment/Japan/KU-H16/2021|2021-01-25|H1N1|PA|EPI ISL 17805835
A/duck/Bangladesh/44493/2020|2020-02-22|H9N9|PA|EPI ISL 4073200
A/common teal/Novosibirsk region/3515k/2020|2020-08-29|H3N8|PA|EPI ISL 1240993
A/duck/Bangladesh/18D1577/2021|2021-01-04|H3N2|PA|EPI ISL 9951121
A/duck/Bangladesh/37473/2019|2019-01-16|H8N4|PA|EPI ISL 503495
A/common teal/Chany Lake/213/2020|2020-08-29|H5N2|PA|EPI ISL 1005680
A/duck/Mongolia/447/2018|2018-09-02|H4N6|PA|EPI ISL 697695
A/swan/Shandong/W4322/2020|2020-11-23|H10N4|PA|EPI ISL 14770171
A/egret/Anhui/A1-267/2020|2020-10-01|H3N8|PA|EPI ISL 17638577
A/garganey/Bangladesh/38920/2019|2019-02-07|H7N4|PA|EPI ISL 503509
A/environment/Kagoshima/KU-C6/2021|2021-12-13|H4N2|PA|EPI ISL 17655673
A/duck/Tochigi/090114/2022|2022-01-18|H10N7|PA|EPI ISL 18720801
A/mallard/Korea/KNU-24/2023|2023-03-15|H11N9|PA|EPI ISL 18698935
A/mallard/Yakutia/47/2020|2020-08-13|H7N7|PA|EPI ISL 1081358
A/Wild bird/South Korea/Q23-324-4/2023|2023-10-18|H3N8|PA|EPI ISL 18900623
A/environment/Kagoshima/KU-C8/2023|2023-12-19|H3N6|PA|EPI ISL 18770643
A/duck/Bangladesh/17D1968/2022|2022-08-18|H3N6|PA|EPI ISL 18983955
A/environment/Kagoshima/KU-E2/2023|2023-11-13|H3N2|PA|EPI ISL 18634699
A/spot billed/Korea/KNU-11/2022|2022-02-09|H7N7|PA|EPI ISL 18698868
A/Brown-headed gull/Qinghai/06-HDT-2/2022|2022-06-20|H10N7|PA|EPI ISL 19004956
A/whooper swan/Shanxi/SX126/2020|2020-11-10|H9N2|PA|EPI ISL 1760456
A/chicken/China/Cangzhou03/2023|2023-12-28|H5N1|PA|EPI ISL 19592785
A/Common Teal/South Korea/W821/2022|2022-11-14|H5N1|PA|EPI ISL 19220046
A/environment/Bangladesh/52224/2022|2022-01-30|H10N4|PA|EPI ISL 15620317
A/duck/Tottori/311215/2020|2020-12-15|H5N2|PA|EPI ISL 6780573
A/duck/Bangladesh/19-D-2266/2023|2023-11-22|H4N6|PA|EPI ISL 19187909
A/duck/Bangladesh/WF-504/2024|2024-01-22|H7N4|PA|EPI ISL 19180879
A/Zhejiang/CNIC-ZJU01/2023|2023-12-15|H10N5|PA|EPI ISL 18846022
A/wild bird/Hubei/YYH40/2024|2024-02-19|H10N5|PA|EPI ISL 19504791
A/environment/Kagoshima/KU-24-J2/2024 |2024-11-11|H3N8|PA|EPI ISL 19645357
A/Wildbird/Bangladesh/WB-015-T/2023|2023-01-26|H5N1|PA|EPI ISL 18983997
A/Ruddy Shelduck/Qinghai/JXG-721-19/2023|2023-07-21|H10N5|PA|EPI ISL 19214962
A/duck/Bangladesh/17-D-2338/2024|2024-02-22|H5N1|PA|EPI ISL 19183994
A Mandarin Duck Korea 24WF266-5P 2024 H5N1|2024 11 13| PA 9th|
A Wild Goose Korea 24WF894-1P 2025 H5N1|2025 03 10| PA 40th|
A Egret Korea 24WC176 2025 H5N1|2025 01 31| PA 34th|
A Mandarin Duck Korea 24WF743-8P 2025 H5N1|2025 02 10| PA 35th|
A Wild Duck Korea 24WF159-10P 2024 H5N1|2024 10 28| PA 7th|
A Mandarin Duck Korea 24WS005-2 H5N1|2024 10 14| PA 2nd|
A Common Buzzard Korea 24WC267 2025 H5N1|2025 03 14| PA 41th|
A Wild Duck 24WF318-1P 2024 H5N1|2024 11 25| PA 11th|
A Leopard Cat Korea 24WM130 2025 H5N1|2025 03 16 PA WM-1st|
A Bean Goose Korea 24WC196 2025 H5N1|2025 02 11| PA 36th|
A Vulture Korea 24WC103 2024 H5N1|2024 12 30| PA 26th|
A Wild Duck 24WF364-8P 2024 H5N1|2024 12 3| PA 12th|
A Wild Duck Korea 24WF653-16P 2025 H5N1|2025 01 14| PA 30th|
A/Em/Korea/24WF382-7P/2024|2024-12-04|H5N1|PA|EPI ISL
A Eurasian Wigeon 24WF382-4P 2024 H5N1|2024 12 04| PA 14th|
A Spot billed Duck Korea 24WS018-1 2024 H5N1|2024 11 12| PA 8th| A
Peregrine Falcon Korea 24WC084 2024 H5N1|2024 12 23| PA 23th|
A/duck/Tottori/NK37-40/2024|2024-10-29|H5N1|PA|EPI ISL 19741594
A Egret Korea 24WC158 2025 H5N1|2025 01 20| PA 31th|
A Egret Korea 24WC159 2025 H5N1|2025 01 20| PA 31th|
A Egret Korea 24WC160 2025 H5N1|2025 01 20| PA 31th|
A Bean Goose Korea 24WC172 2025 H5N1|2025 01 27| PA 33th|
A Egret Korea 24WC069 2024 H5N1|2024 12 16| PA 18th|
A Egret Korea 24WC079 2024 H5N1|2024 12 19| PA 21th|
A Egret Korea 24WC068 2024 H5N1|2024 12 16| PA 18th|
A Egret Korea 24WC070 2024 H5N1|2024 12 16| PA 18th|
A Egret Korea 24WC083 2024 H5N1|2024 12 20| PA 22th|
A Egret Korea 24WC081 2024 H5N1|2024 12 19| PA 21th|
A Egret Korea 24WC071 2024 H5N1|2024 12 16| PA 18th|
A Egret Korea 24WC082 2024 H5N1|2024 12 19| PA 21th|
A Herring Gull Korea 24WC224 2025 H5N1|2025 02 19| PA 38th|
A Egret Korea 24WC104 2025 H5N1|2025 01 02| PA 27th|
A Egret Korea 24WC140 2025 H5N1|2025 01 14| PA 29th|
A Egret Korea 24WC141 2025 H5N1|2025 01 14| PA 29th|
A Gray Heron Korea 24WC269 2025 H5N1|2025 03 14| PA 42th|
A Egret Korea 24WC128 2025 H5N1|2025 01 10| PA 28th|
A Egret Korea 24WC127 2025 H5N1|2025 01 10| PA 28th|
A Egret Korea 24WC142 2025 H5N1|2025 01 14| PA 29th|
A Great Cormorant Korea 24WC275 2025 H5N1|2025 03 24| PA 43th|
Colored ranges
Highly pathogenic avian influenza
Tree scale: 0.01

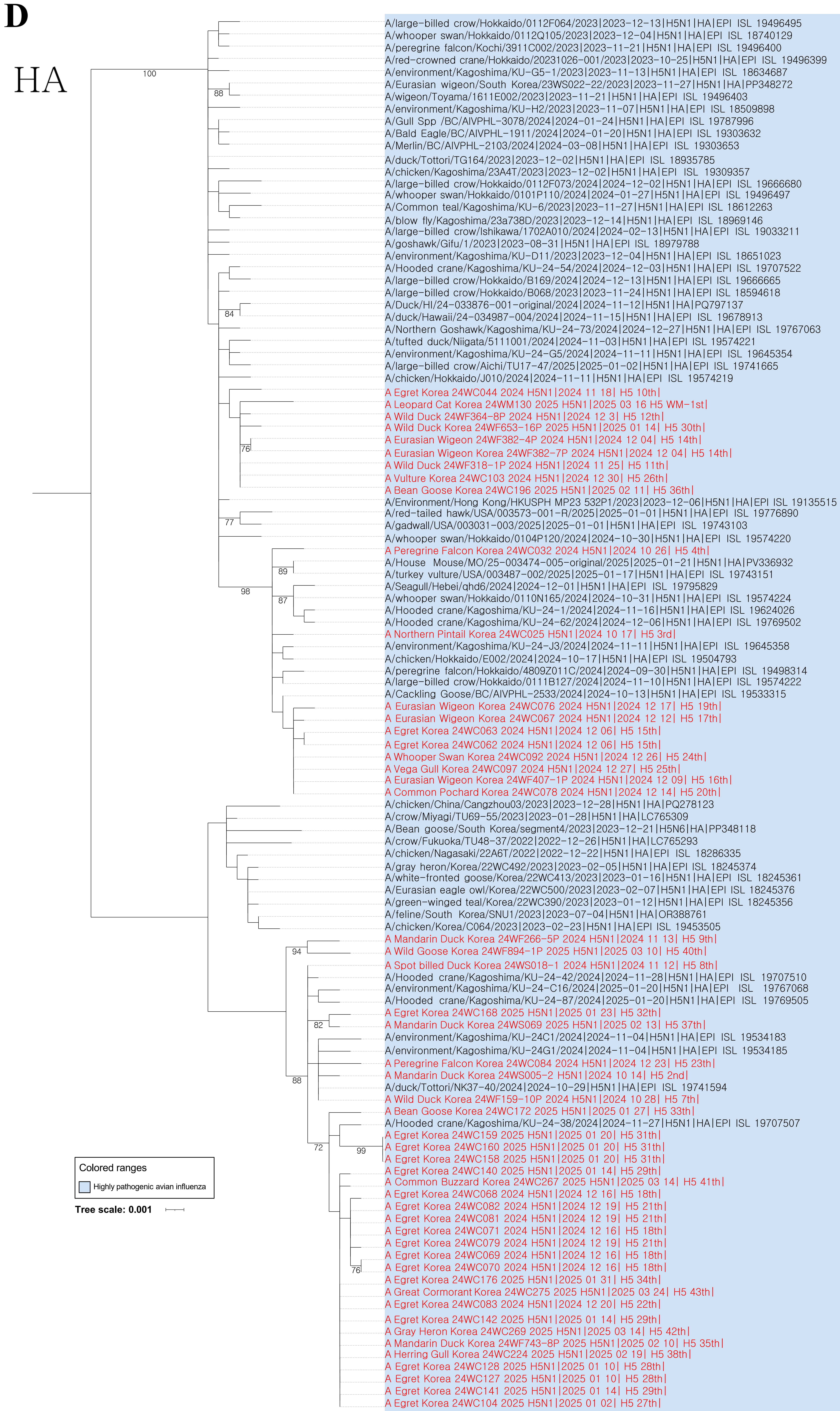
D
HA
100
88
84
76
77
98
89
87
94
82
88
72
99
76
A/large-billed crow/Hokkaido/0112F064/2023|2023-12-13|H5N1|HA|EPI ISL 19496495
A/whooper swan/Hokkaido/0112Q105/2023|2023-12-04|H5N1|HA|EPI ISL 18740129
A/peregrine falcon/Kochi/3911C002/2023|2023-11-21|H5N1|HA|EPI ISL 19496400
A/red-crowned crane/Hokkaido/20231026-001/2023|2023-10-25|H5N1|HA|EPI ISL 19496399
A/environment/Kagoshima/KU-G5-1/2023|2023-11-13|H5N1|HA|EPI ISL 18634687
A/Eurasian wigeon/South Korea/23WS022-22/2023|2023-11-27|H5N1|HA|PP348272
A/wigeon/Toyama/1611E002/2023|2023-11-21|H5N1|HA|EPI ISL 19496403
A/environment/Kagoshima/KU-H2/2023|2023-11-07|H5N1|HA|EPI ISL 18509898
A/Gull Spp /BC/AIVPHL-3078/2024|2024-01-24|H5N1|HA|EPI ISL 19787996
A/Bald Eagle/BC/AIVPHL-1911/2024|2024-01-20|H5N1|HA|EPI ISL 19303632
A/Merlin/BC/AIVPHL-2103/2024|2024-03-08|H5N1|HA|EPI ISL 19303653
A/duck/Tottori/TG164/2023|2023-12-02|H5N1|HA|EPI ISL 18935785
A/chicken/Kagoshima/23A4T/2023|2023-12-02|H5N1|HA|EPI ISL 19309357
A/large-billed crow/Hokkaido/0112F073/2024|2024-12-02|H5N1|HA|EPI ISL 19666680
A/whooper swan/Hokkaido/0101P110/2024|2024-01-27|H5N1|HA|EPI ISL 19496497
A/Common teal/Kagoshima/KU-6/2023|2023-11-27|H5N1|HA|EPI ISL 18612263
A/blow fly/Kagoshima/23a738D/2023|2023-12-14|H5N1|HA|EPI ISL 18969146
A/large-billed crow/Ishikawa/1702A010/2024|2024-02-13|H5N1|HA|EPI ISL 19033211
A/goshawk/Gifu/1/2023|2023-08-31|H5N1|HA|EPI ISL 18979788
A/environment/Kagoshima/KU-D11/2023|2023-12-04|H5N1|HA|EPI ISL 18651023
A/Hooded crane/Kagoshima/KU-24-54/2024|2024-12-03|H5N1|HA|EPI ISL 19707522
A/large-billed crow/Hokkaido/B169/2024|2024-12-13|H5N1|HA|EPI ISL 19666665
A/large-billed crow/Hokkaido/B068/2023|2023-11-24|H5N1|HA|EPI ISL 18594618
A/Duck/HI/24-033876-001-original/2024|2024-11-12|H5N1|HA|PQ797137
A/duck/Hawaii/24-034987-004/2024|2024-11-15|H5N1|HA|EPI ISL 19678913
A/Northern Goshawk/Kagoshima/KU-24-73/2024|2024-12-27|H5N1|HA|EPI ISL 19767063
A/tufted duck/Niigata/5111001/2024|2024-11-03|H5N1|HA|EPI ISL 19574221
A/environment/Kagoshima/KU-24-G5/2024|2024-11-11|H5N1|HA|EPI ISL 19645354
A/large-billed crow/Aichi/TU17-47/2025|2025-01-02|H5N1|HA|EPI ISL 19741665
A/chicken/Hokkaido/J010/2024|2024-11-11|H5N1|HA|EPI ISL 19574219
A Egret Korea 24WC044 2024 H5N1|2024 11 18| H5 10th|
A Leopard Cat Korea 24WM130 2025 H5N1|2025 03 16 H5 WM-1st|
A Wild Duck 24WF364-8P 2024 H5N1|2024 12 3| H5 12th|
A Wild Duck Korea 24WF653-16P 2025 H5N1|2025 01 14| H5 30th|
A Eurasian Wigeon 24WF382-4P 2024 H5N1|2024 12 04| H5 14th|
A Eurasian Wigeon Korea 24WF382-7P 2024 H5N1|2024 12 04| H5 14th|
A Wild Duck 24WF318-1P 2024 H5N1|2024 11 25| H5 11th|
A Vulture Korea 24WC103 2024 H5N1|2024 12 30| H5 26th|
A Bean Goose Korea 24WC196 2025 H5N1|2025 02 11| H5 36th|
A/Environment/Hong Kong/HKUSPH MP23 532P1/2023|2023-12-06|H5N1|HA|EPI ISL 19135515
A/red-tailed hawk/USA/003573-001-R/2025|2025-01-01|H5N1|HA|EPI ISL 19776890
A/gadwall/USA/003031-003/2025|2025-01-01|H5N1|HA|EPI ISL 19743103
A/whooper swan/Hokkaido/0104P120/2024|2024-10-30|H5N1|HA|EPI ISL 19574220
A Peregrine Falcon Korea 24WC032 2024 H5N1|2024 10 26| H5 4th|
A/House Mouse/MO/25-003474-005-original/2025|2025-01-21|H5N1|HA|PV336932
A/turkey vulture/USA/003487-002/2025|2025-01-17|H5N1|HA|EPI ISL 19743151
A/Seagull/Hebei/qhd6/2024|2024-12-01|H5N1|HA|EPI ISL 19795829
A/whooper swan/Hokkaido/0110N165/2024|2024-10-31|H5N1|HA|EPI ISL 19574224
A/Hooded crane/Kagoshima/KU-24-1/2024|2024-11-16|H5N1|HA|EPI ISL 19624026
A/Hooded crane/Kagoshima/KU-24-62/2024|2024-12-06|H5N1|HA|EPI ISL 19769502
A Northern Pintail Korea 24WC025 H5N1|2024 10 17| H5 3rd|
A/environment/Kagoshima/KU-24-J3/2024|2024-11-11|H5N1|HA|EPI ISL 19645358
A/chicken/Hokkaido/E002/2024|2024-10-17|H5N1|HA|EPI ISL 19504793
A/peregrine falcon/Hokkaido/4809Z011C/2024|2024-09-30|H5N1|HA|EPI ISL 19498314
A/large-billed crow/Hokkaido/0111B127/2024|2024-11-10|H5N1|HA|EPI ISL 19574222
A/Cackling Goose/BC/AIVPHL-2533/2024|2024-10-13|H5N1|HA|EPI ISL 19533315
A Eurasian Wigeon Korea 24WC076 2024 H5N1|2024 12 17| H5 19th|
A Eurasian Wigeon Korea 24WC067 2024 H5N1|2024 12 12| H5 17th|
A Egret Korea 24WC063 2024 H5N1|2024 12 06| H5 15th|
A Egret Korea 24WC062 2024 H5N1|2024 12 06| H5 15th|
A Whooper Swan Korea 24WC092 2024 H5N1|2024 12 26| H5 24th|
A Vega Gull Korea 24WC097 2024 H5N1|2024 12 27| H5 25th|
A Eurasian Wigeon Korea 24WF407-1P 2024 H5N1|2024 12 09| H5 16th|
A Common Pochard Korea 24WC078 2024 H5N1|2024 12 14| H5 20th|
A/chicken/China/Cangzhou03/2023|2023-12-28|H5N1|HA|PQ278123
A/crow/Miyagi/TU69-55/2023|2023-01-28|H5N1|HA|LC765309
A/Bean goose/South Korea/segment4/2023|2023-12-21|H5N6|HA|PP348118
A/crow/Fukuoka/TU48-37/2022|2022-12-26|H5N1|HA|LC765293
A/chicken/Nagasaki/22A6T/2022|2022-12-22|H5N1|HA|EPI ISL 18286335
A/gray heron/Korea/22WC492/2023|2023-02-05|H5N1|HA|EPI ISL 18245374
A/white-fronted goose/Korea/22WC413/2023|2023-01-16|H5N1|HA|EPI ISL 18245361
A/Eurasian eagle owl/Korea/22WC500/2023|2023-02-07|H5N1|HA|EPI ISL 18245376
A/green-winged teal/Korea/22WC390/2023|2023-01-12|H5N1|HA|EPI ISL 18245356
A/feline/South Korea/SNU1/2023|2023-07-04|H5N1|HA|OR388761
A/chicken/Korea/C064/2023|2023-02-23|H5N1|HA|EPI ISL 19453505
A Mandarin Duck Korea 24WF266-5P 2024 H5N1|2024 11 13| H5 9th|
A Wild Goose Korea 24WF894-1P 2025 H5N1|2025 03 10| H5 40th|
A Spot billed Duck Korea 24WS018-1 2024 H5N1|2024 11 12| H5 8th|
A/Hooded crane/Kagoshima/KU-24-42/2024|2024-11-28|H5N1|HA|EPI ISL 19707510
A/environment/Kagoshima/KU-24-C16/2024|2025-01-20|H5N1|HA|EPI ISL 19767068
A/Hooded crane/Kagoshima/KU-24-87/2024|2025-01-20|H5N1|HA|EPI ISL 19769505
A Egret Korea 24WC168 2025 H5N1|2025 01 23| H5 32th|
A Mandarin Duck Korea 24WS069 2025 H5N1|2025 02 13| H5 37th|
A/environment/Kagoshima/KU-24C1/2024|2024-11-04|H5N1|HA|EPI ISL 19534183
A/environment/Kagoshima/KU-24G1/2024|2024-11-04|H5N1|HA|EPI ISL 19534185
A Peregrine Falcon Korea 24WC084 2024 H5N1|2024 12 23| H5 23th|
A Mandarin Duck Korea 24WS005-2 H5N1|2024 10 14| H5 2nd|
A/duck/Tottori/NK37-40/2024|2024-10-29|H5N1|HA|EPI ISL 19741594
A Wild Duck Korea 24WF159-10P 2024 H5N1|2024 10 28| H5 7th|
A Bean Goose Korea 24WC172 2025 H5N1|2025 01 27| H5 33th|
A/Hooded crane/Kagoshima/KU-24-38/2024|2024-11-27|H5N1|HA|EPI ISL 19707507
A Egret Korea 24WC159 2025 H5N1|2025 01 20| H5 31th|
A Egret Korea 24WC160 2025 H5N1|2025 01 20| H5 31th|
A Egret Korea 24WC158 2025 H5N1|2025 01 20| H5 31th|
A Egret Korea 24WC140 2025 H5N1|2025 01 14| H5 29th|
A Common Buzzard Korea 24WC267 2025 H5N1|2025 03 14| H5 41th|
A Egret Korea 24WC068 2024 H5N1|2024 12 16| H5 18th|
A Egret Korea 24WC082 2024 H5N1|2024 12 19| H5 21th|
A Egret Korea 24WC081 2024 H5N1|2024 12 19| H5 21th|
A Egret Korea 24WC071 2024 H5N1|2024 12 16| H5 18th|
A Egret Korea 24WC079 2024 H5N1|2024 12 19| H5 21th|
A Egret Korea 24WC069 2024 H5N1|2024 12 16| H5 18th|
A Egret Korea 24WC070 2024 H5N1|2024 12 16| H5 18th|
A Egret Korea 24WC176 2025 H5N1|2025 01 31| H5 34th|
A Great Cormorant Korea 24WC275 2025 H5N1|2025 03 24| H5 43th|
A Egret Korea 24WC083 2024 H5N1|2024 12 20| H5 22th|
A Egret Korea 24WC142 2025 H5N1|2025 01 14| H5 29th|
A Gray Heron Korea 24WC269 2025 H5N1|2025 03 14| H5 42th|
A Mandarin Duck Korea 24WF743-8P 2025 H5N1|2025 02 10| H5 35th|
A Herring Gull Korea 24WC224 2025 H5N1|2025 02 19| H5 38th|
A Egret Korea 24WC128 2025 H5N1|2025 01 10| H5 28th|
A Egret Korea 24WC127 2025 H5N1|2025 01 10| H5 28th|
A Egret Korea 24WC141 2025 H5N1|2025 01 14| H5 29th|
A Egret Korea 24WC104 2025 H5N1|2025 01 02| H5 27th|
Colored ranges
Highly pathogenic avian influenza
Tree scale: 0.001

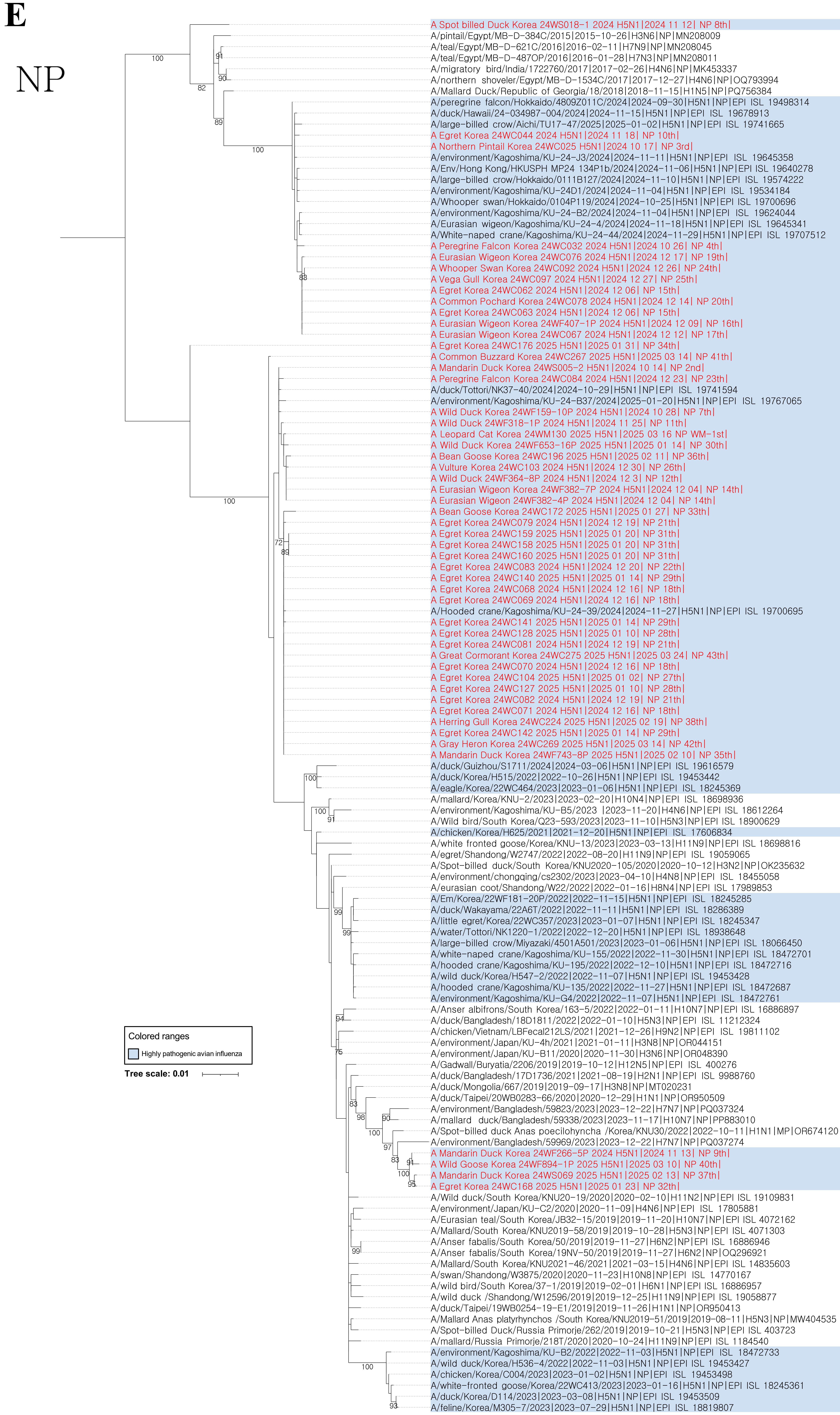
E
NP
A Spot billed Duck Korea 24WS018-1 2024 H5N1|2024 11 12| NP 8th|
A/pintail/Egypt/MB-D-384C/2015|2015-10-26|H3N6|NP|MN208009
A/teal/Egypt/MB-D-621C/2016|2016-02-11|H7N9|NP|MN208045
A/teal/Egypt/MB-D-487OP/2016|2016-01-28|H7N3|NP|MN208011
A/migratory bird/India/1722760/2017|2017-02-26|H4N6|NP|MK453337
A/northern shoveler/Egypt/MB-D-1534C/2017|2017-12-27|H4N6|NP|OQ793994
A/Mallard Duck/Republic of Georgia/18/2018|2018-11-15|H1N5|NP|PQ756384
A/peregrine falcon/Hokkaido/4809Z011C/2024|2024-09-30|H5N1|NP|EPI ISL 19498314
A/duck/Hawaii/24-034987-004/2024|2024-11-15|H5N1|NP|EPI ISL 19678913
A/large-billed crow/Aichi/TU17-47/2025|2025-01-02|H5N1|NP|EPI ISL 19741665
A Egret Korea 24WC044 2024 H5N1|2024 11 18| NP 10th|
A Northern Pintail Korea 24WC025 H5N1|2024 10 17| NP 3rd|
A/environment/Kagoshima/KU-24-J3/2024|2024-11-11|H5N1|NP|EPI ISL 19645358
A/Env/Hong Kong/HKUSPH MP24 134P1b/2024|2024-11-06|H5N1|NP|EPI ISL 19640278
A/large-billed crow/Hokkaido/0111B127/2024|2024-11-10|H5N1|NP|EPI ISL 19574222
A/environment/Kagoshima/KU-24D1/2024|2024-11-04|H5N1|NP|EPI ISL 19534184
A/Whooper swan/Hokkaido/0104P119/2024|2024-10-25|H5N1|NP|EPI ISL 19700696
A/environment/Kagoshima/KU-24-B2/2024|2024-11-04|H5N1|NP|EPI ISL 19624044
A/Eurasian wigeon/Kagoshima/KU-24-4/2024|2024-11-18|H5N1|NP|EPI ISL 19645341
A/White-naped crane/Kagoshima/KU-24-44/2024|2024-11-29|H5N1|NP|EPI ISL 19707512
A Peregrine Falcon Korea 24WC032 2024 H5N1|2024 10 26| NP 4th|
A Eurasian Wigeon Korea 24WC076 2024 H5N1|2024 12 17| NP 19th|
A Whooper Swan Korea 24WC092 2024 H5N1|2024 12 26| NP 24th|
A Vega Gull Korea 24WC097 2024 H5N1|2024 12 27| NP 25th|
A Egret Korea 24WC062 2024 H5N1|2024 12 06| NP 15th|
A Common Pochard Korea 24WC078 2024 H5N1|2024 12 14| NP 20th|
A Egret Korea 24WC063 2024 H5N1|2024 12 06| NP 15th|
A Eurasian Wigeon Korea 24WF407-1P 2024 H5N1|2024 12 09| NP 16th|
A Eurasian Wigeon Korea 24WC067 2024 H5N1|2024 12 12| NP 17th|
A Egret Korea 24WC176 2025 H5N1|2025 01 31| NP 34th|
A Common Buzzard Korea 24WC267 2025 H5N1|2025 03 14| NP 41th|
A Mandarin Duck Korea 24WS005-2 H5N1|2024 10 14| NP 2nd|
A Peregrine Falcon Korea 24WC084 2024 H5N1|2024 12 23| NP 23th|
A/duck/Tottori/NK37-40/2024|2024-10-29|H5N1|NP|EPI ISL 19741594
A/environment/Kagoshima/KU-24-B37/2024|2025-01-20|H5N1|NP|EPI ISL 19767065
A Wild Duck Korea 24WF159-10P 2024 H5N1|2024 10 28| NP 7th|
A Wild Duck 24WF318-1P 2024 H5N1|2024 11 25| NP 11th|
A Leopard Cat Korea 24WM130 2025 H5N1|2025 03 16 NP WM-1st|
A Wild Duck Korea 24WF653-16P 2025 H5N1|2025 01 14| NP 30th|
A Bean Goose Korea 24WC196 2025 H5N1|2025 02 11| NP 36th|
A Vulture Korea 24WC103 2024 H5N1|2024 12 30| NP 26th|
A Wild Duck 24WF364-8P 2024 H5N1|2024 12 3| NP 12th|
A Eurasian Wigeon Korea 24WF382-7P 2024 H5N1|2024 12 04| NP 14th|
A Eurasian Wigeon 24WF382-4P 2024 H5N1|2024 12 04| NP 14th|
A Bean Goose Korea 24WC172 2025 H5N1|2025 01 27| NP 33th|
A Egret Korea 24WC079 2024 H5N1|2024 12 19| NP 21th|
A Egret Korea 24WC159 2025 H5N1|2025 01 20| NP 31th|
A Egret Korea 24WC158 2025 H5N1|2025 01 20| NP 31th|
A Egret Korea 24WC160 2025 H5N1|2025 01 20| NP 31th|
A Egret Korea 24WC083 2024 H5N1|2024 12 20| NP 22th|
A Egret Korea 24WC140 2025 H5N1|2025 01 14| NP 29th|
A Egret Korea 24WC068 2024 H5N1|2024 12 16| NP 18th|
A Egret Korea 24WC069 2024 H5N1|2024 12 16| NP 18th|
A/Hooded crane/Kagoshima/KU-24-39/2024|2024-11-27|H5N1|NP|EPI ISL 19700695
A Egret Korea 24WC141 2025 H5N1|2025 01 14| NP 29th|
A Egret Korea 24WC128 2025 H5N1|2025 01 10| NP 28th|
A Egret Korea 24WC081 2024 H5N1|2024 12 19| NP 21th|
A Great Cormorant Korea 24WC275 2025 H5N1|2025 03 24| NP 43th|
A Egret Korea 24WC070 2024 H5N1|2024 12 16| NP 18th|
A Egret Korea 24WC104 2025 H5N1|2025 01 02| NP 27th|
A Egret Korea 24WC127 2025 H5N1|2025 01 10| NP 28th|
A Egret Korea 24WC082 2024 H5N1|2024 12 19| NP 21th|
A Egret Korea 24WC071 2024 H5N1|2024 12 16| NP 18th|
A Herring Gull Korea 24WC224 2025 H5N1|2025 02 19| NP 38th|
A Egret Korea 24WC142 2025 H5N1|2025 01 14| NP 29th|
A Gray Heron Korea 24WC269 2025 H5N1|2025 03 14| NP 42th|
A Mandarin Duck Korea 24WF743-8P 2025 H5N1|2025 02 10| NP 35th|
A/duck/Guizhou/S1711/2024|2024-03-06|H5N1|NP|EPI ISL 19616579
A/duck/Korea/H515/2022|2022-10-26|H5N1|NP|EPI ISL 19453442
A/eagle/Korea/22WC464/2023|2023-01-06|H5N1|NP|EPI ISL 18245369
A/mallard/Korea/KNU-2/2023|2023-02-20|H10N4|NP|EPI ISL 18698936
A/environment/Kagoshima/KU-B5/2023 |2023-11-20|H4N6|NP|EPI ISL 18612264
A/Wild bird/South Korea/Q23-593/2023|2023-11-10|H5N3|NP|EPI ISL 18900629
A/chicken/Korea/H625/2021|2021-12-20|H5N1|NP|EPI ISL 17606834
A/white fronted goose/Korea/KNU-13/2023|2023-03-13|H11N9|NP|EPI ISL 18698816
A/egret/Shandong/W2747/2022|2022-08-20|H11N9|NP|EPI ISL 19059065
A/Spot-billed duck/South Korea/KNU2020-105/2020|2020-10-12|H3N2|NP|OK235632
A/environment/chongqing/cs2302/2023|2023-04-10|H4N8|NP|EPI ISL 18455058
A/eurasian coot/Shandong/W22/2022|2022-01-16|H8N4|NP|EPI ISL 17989853
A/Em/Korea/22WF181-20P/2022|2022-11-15|H5N1|NP|EPI ISL 18245285
A/duck/Wakayama/22A6T/2022|2022-11-11|H5N1|NP|EPI ISL 18286389
A/little egret/Korea/22WC357/2023|2023-01-07|H5N1|NP|EPI ISL 18245347
A/water/Tottori/NK1220-1/2022|2022-12-20|H5N1|NP|EPI ISL 18938648
A/large-billed crow/Miyazaki/4501A501/2023|2023-01-06|H5N1|NP|EPI ISL 18066450
A/white-naped crane/Kagoshima/KU-155/2022|2022-11-30|H5N1|NP|EPI ISL 18472701
A/hooded crane/Kagoshima/KU-195/2022|2022-12-10|H5N1|NP|EPI ISL 18472716
A/wild duck/Korea/H547-2/2022|2022-11-07|H5N1|NP|EPI ISL 19453428
A/hooded crane/Kagoshima/KU-135/2022|2022-11-27|H5N1|NP|EPI ISL 18472687
A/environment/Kagoshima/KU-G4/2022|2022-11-07|H5N1|NP|EPI ISL 18472761
A/Anser albifrons/South Korea/163-5/2022|2022-01-11|H10N7|NP|EPI ISL 16886897
A/duck/Bangladesh/18D1811/2022|2022-01-10|H5N3|NP|EPI ISL 11212324
A/chicken/Vietnam/LBFecal212LS/2021|2021-12-26|H9N2|NP|EPI ISL 19811102
A/environment/Japan/KU-4h/2021|2021-01-11|H3N8|NP|OR044151
A/environment/Japan/KU-B11/2020|2020-11-30|H3N6|NP|OR048390
A/Gadwall/Buryatia/2206/2019|2019-10-12|H12N5|NP|EPI ISL 400276
A/duck/Bangladesh/17D1736/2021|2021-08-19|H2N1|NP|EPI ISL 9988760
A/duck/Mongolia/667/2019|2019-09-17|H3N8|NP|MT020231
A/duck/Taipei/20WB0283-66/2020|2020-12-29|H1N1|NP|OR950509
A/environment/Bangladesh/59823/2023|2023-12-22|H7N7|NP|PQ037324
A/mallard duck/Bangladesh/59338/2023|2023-11-17|H10N7|NP|PP883010
A/Spot-billed duck Anas poecilohyncha /Korea/KNU30/2022|2022-10-11|H1N1|MP|OR674120
A/environment/Bangladesh/59969/2023|2023-12-22|H7N7|NP|PQ037274
A Mandarin Duck Korea 24WF266-5P 2024 H5N1|2024 11 13| NP 9th|
A Wild Goose Korea 24WF894-1P 2025 H5N1|2025 03 10| NP 40th|
A Mandarin Duck Korea 24WS069 2025 H5N1|2025 02 13| NP 37th|
A Egret Korea 24WC168 2025 H5N1|2025 01 23| NP 32th|
A/Wild duck/South Korea/KNU20-19/2020|2020-02-10|H11N2|NP|EPI ISL 19109831
A/environment/Japan/KU-C2/2020|2020-11-09|H4N6|NP|EPI ISL 17805881
A/Eurasian teal/South Korea/JB32-15/2019|2019-11-20|H10N7|NP|EPI ISL 4072162
A/Mallard/South Korea/KNU2019-58/2019|2019-10-28|H5N3|NP|EPI ISL 4071303
A/Anser fabalis/South Korea/50/2019|2019-11-27|H6N2|NP|EPI ISL 16886946
A/Anser fabalis/South Korea/19NV-50/2019|2019-11-27|H6N2|NP|OQ296921
A/Mallard/South Korea/KNU2021-46/2021|2021-03-15|H4N6|NP|EPI ISL 14835603
A/swan/Shandong/W3875/2020|2020-11-23|H10N8|NP|EPI ISL 14770167
A/wild bird/South Korea/37-1/2019|2019-02-01|H6N1|NP|EPI ISL 16886957
A/wild duck /Shandong/W12596/2019|2019-12-25|H11N9|NP|EPI ISL 19058877
A/duck/Taipei/19WB0254-19-E1/2019|2019-11-26|H1N1|NP|OR950413
A/Mallard Anas platyrhynchos /South Korea/KNU2019-51/2019|2019-08-11|H5N3|NP|MW404535
A/Spot-billed Duck/Russia Primorje/262/2019|2019-10-21|H5N3|NP|EPI ISL 403723
A/mallard/Russia Primorje/218T/2020|2020-10-24|H11N9|NP|EPI ISL 1184540
A/environment/Kagoshima/KU-B2/2022|2022-11-03|H5N1|NP|EPI ISL 18472733
A/wild duck/Korea/H536-4/2022|2022-11-03|H5N1|NP|EPI ISL 19453427
A/chicken/Korea/C004/2023|2023-01-02|H5N1|NP|EPI ISL 19453498
A/white-fronted goose/Korea/22WC413/2023|2023-01-16|H5N1|NP|EPI ISL 18245361
A/duck/Korea/D114/2023|2023-03-08|H5N1|NP|EPI ISL 19453509
A/feline/Korea/M305-7/2023|2023-07-29|H5N1|NP|EPI ISL 18819807
Colored ranges
Highly pathogenic avian influenza
Tree scale: 0.01

F

NA

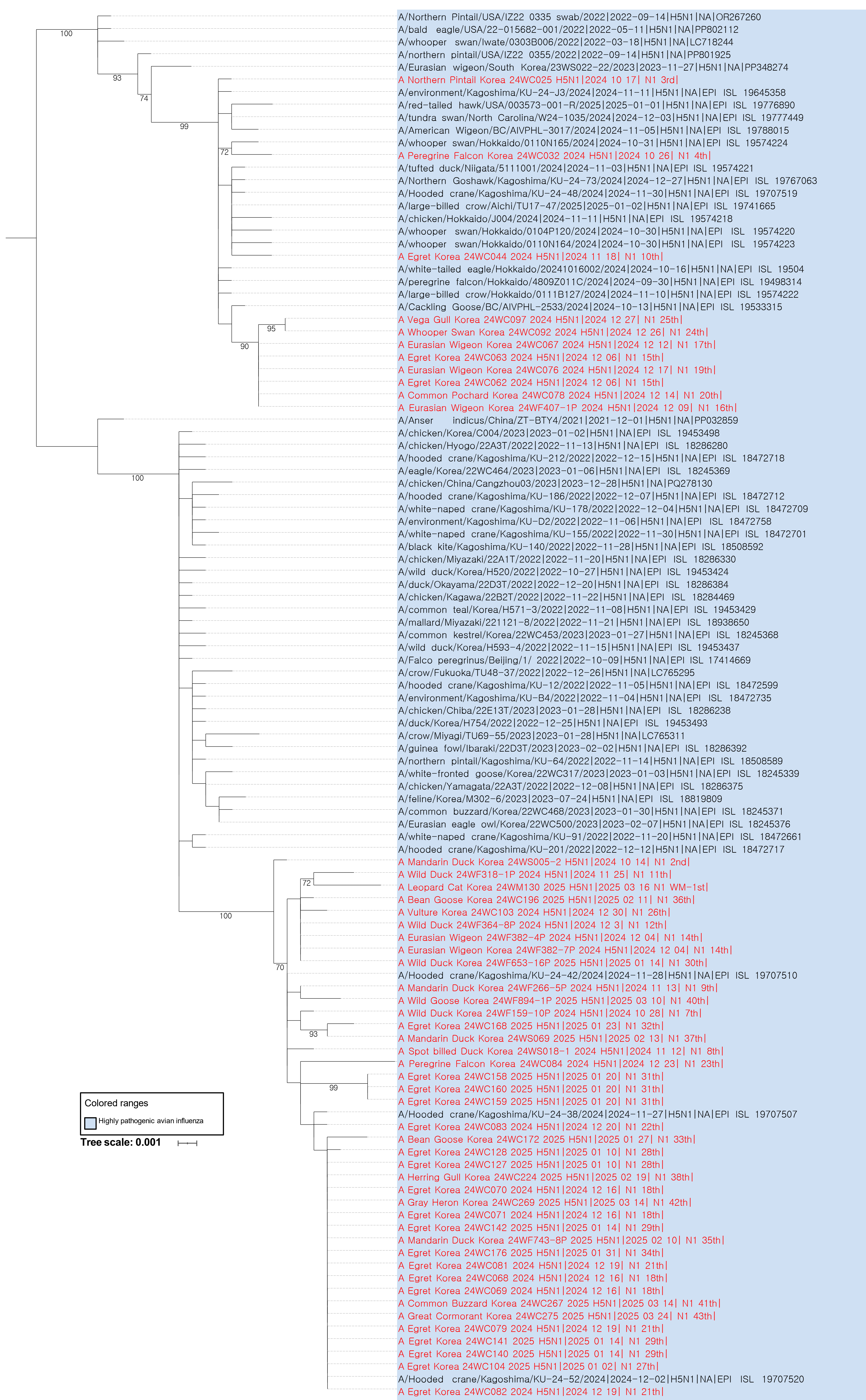

A/Northern Pintail/USA/IZ22 0335 swab/2022|2022-09-14|H5N1|NA|OR267260
A/bald eagle/USA/22-015682-001/2022|2022-05-11|H5N1|NA|PP802112
A/whooper swan/Iwate/0303B006/2022|2022-03-18|H5N1|NA|LC718244
A/northern pintail/USA/IZ22 0355/2022|2022-09-14|H5N1|NA|PP801925
A/Eurasian wigeon/South Korea/23WS022-22/2023|2023-11-27|H5N1|NA|PP348274
A Northern Pintail Korea 24WC025 H5N1|2024 10 17| N1 3rd|
A/environment/Kagoshima/KU-24-J3/2024|2024-11-11|H5N1|NA|EPI ISL 19645358
A/red-tailed hawk/USA/003573-001-R/2025|2025-01-01|H5N1|NA|EPI ISL 19776890
A/tundra swan/North Carolina/W24-1035/2024|2024-12-03|H5N1|NA|EPI ISL 19777449
A/American Wigeon/BC/AIVPHL-3017/2024|2024-11-05|H5N1|NA|EPI ISL 19788015
A/whooper swan/Hokkaido/0110N165/2024|2024-10-31|H5N1|NA|EPI ISL 19574224
A Peregrine Falcon Korea 24WC032 2024 H5N1|2024 10 26| N1 4th|
A/tufted duck/Niigata/5111001/2024|2024-11-03|H5N1|NA|EPI ISL 19574221
A/Northern Goshawk/Kagoshima/KU-24-73/2024|2024-12-27|H5N1|NA|EPI ISL 19767063
A/Hooded crane/Kagoshima/KU-24-48/2024|2024-11-30|H5N1|NA|EPI ISL 19707519
A/large-billed crow/Aichi/TU17-47/2025|2025-01-02|H5N1|NA|EPI ISL 19741665
A/chicken/Hokkaido/J004/2024|2024-11-11|H5N1|NA|EPI ISL 19574218
A/whooper swan/Hokkaido/0104P120/2024|2024-10-30|H5N1|NA|EPI ISL 19574220
A/whooper swan/Hokkaido/0110N164/2024|2024-10-30|H5N1|NA|EPI ISL 19574223
A Egret Korea 24WC044 2024 H5N1|2024 11 18| N1 10th|
A/white-tailed eagle/Hokkaido/20241016002/2024|2024-10-16|H5N1|NA|EPI ISL 19504
A/peregrine falcon/Hokkaido/4809Z011C/2024|2024-09-30|H5N1|NA|EPI ISL 19498314
A/large-billed crow/Hokkaido/0111B127/2024|2024-11-10|H5N1|NA|EPI ISL 19574222
A/Cackling Goose/BC/AIVPHL-2533/2024|2024-10-13|H5N1|NA|EPI ISL 19533315
A Vega Gull Korea 24WC097 2024 H5N1|2024 12 27| N1 25th|
A Whooper Swan Korea 24WC092 2024 H5N1|2024 12 26| N1 24th|
A Eurasian Wigeon Korea 24WC067 2024 H5N1|2024 12 12| N1 17th|
A Egret Korea 24WC063 2024 H5N1|2024 12 06| N1 15th|
A Eurasian Wigeon Korea 24WC076 2024 H5N1|2024 12 17| N1 19th|
A Egret Korea 24WC062 2024 H5N1|2024 12 06| N1 15th|
A Common Pochard Korea 24WC078 2024 H5N1|2024 12 14| N1 20th|
A Eurasian Wigeon Korea 24WF407-1P 2024 H5N1|2024 12 09| N1 16th|
A/Anser indicus/China/ZT-BTY4/2021|2021-12-01|H5N1|NA|PP032859
A/chicken/Korea/C004/2023|2023-01-02|H5N1|NA|EPI ISL 19453498
A/chicken/Hyogo/22A3T/2022|2022-11-13|H5N1|NA|EPI ISL 18286280
A/hooded crane/Kagoshima/KU-212/2022|2022-12-15|H5N1|NA|EPI ISL 18472718
A/eagle/Korea/22WC464/2023|2023-01-06|H5N1|NA|EPI ISL 18245369
A/chicken/China/Cangzhou03/2023|2023-12-28|H5N1|NA|PQ278130
A/hooded crane/Kagoshima/KU-186/2022|2022-12-07|H5N1|NA|EPI ISL 18472712
A/white-naped crane/Kagoshima/KU-178/2022|2022-12-04|H5N1|NA|EPI ISL 18472709
A/environment/Kagoshima/KU-D2/2022|2022-11-06|H5N1|NA|EPI ISL 18472758
A/white-naped crane/Kagoshima/KU-155/2022|2022-11-30|H5N1|NA|EPI ISL 18472701
A/black kite/Kagoshima/KU-140/2022|2022-11-28|H5N1|NA|EPI ISL 18508592
A/chicken/Miyazaki/22A1T/2022|2022-11-20|H5N1|NA|EPI ISL 18286330
A/wild duck/Korea/H520/2022|2022-10-27|H5N1|NA|EPI ISL 19453424
A/duck/Okayama/22D3T/2022|2022-12-20|H5N1|NA|EPI ISL 18286384
A/chicken/Kagawa/22B2T/2022|2022-11-22|H5N1|NA|EPI ISL 18284469
A/common teal/Korea/H571-3/2022|2022-11-08|H5N1|NA|EPI ISL 19453429
A/mallard/Miyazaki/221121-8/2022|2022-11-21|H5N1|NA|EPI ISL 18938650
A/common kestrel/Korea/22WC453/2023|2023-01-27|H5N1|NA|EPI ISL 18245368
A/wild duck/Korea/H593-4/2022|2022-11-15|H5N1|NA|EPI ISL 19453437
A/Falco peregrinus/Beijing/1/ 2022|2022-10-09|H5N1|NA|EPI ISL 17414669
A/crow/Fukuoka/TU48-37/2022|2022-12-26|H5N1|NA|LC765295
A/hooded crane/Kagoshima/KU-12/2022|2022-11-05|H5N1|NA|EPI ISL 18472599
A/environment/Kagoshima/KU-B4/2022|2022-11-04|H5N1|NA|EPI ISL 18472735
A/chicken/Chiba/22E13T/2023|2023-01-28|H5N1|NA|EPI ISL 18286238
A/duck/Korea/H754/2022|2022-12-25|H5N1|NA|EPI ISL 19453493
A/crow/Miyagi/TU69-55/2023|2023-01-28|H5N1|NA|LC765311
A/guinea fowl/Ibaraki/22D3T/2023|2023-02-02|H5N1|NA|EPI ISL 18286392
A/northern pintail/Kagoshima/KU-64/2022|2022-11-14|H5N1|NA|EPI ISL 18508589
A/white-fronted goose/Korea/22WC317/2023|2023-01-03|H5N1|NA|EPI ISL 18245339
A/chicken/Yamagata/22A3T/2022|2022-12-08|H5N1|NA|EPI ISL 18286375
A/feline/Korea/M302-6/2023|2023-07-24|H5N1|NA|EPI ISL 18819809
A/common buzzard/Korea/22WC468/2023|2023-01-30|H5N1|NA|EPI ISL 18245371
A/Eurasian eagle owl/Korea/22WC500/2023|2023-02-07|H5N1|NA|EPI ISL 18245376
A/white-naped crane/Kagoshima/KU-91/2022|2022-11-20|H5N1|NA|EPI ISL 18472661
A/hooded crane/Kagoshima/KU-201/2022|2022-12-12|H5N1|NA|EPI ISL 18472717
A Mandarin Duck Korea 24WS005-2 H5N1|2024 10 14| N1 2nd|
A Wild Duck 24WF318-1P 2024 H5N1|2024 11 25| N1 11th|
A Leopard Cat Korea 24WM130 2025 H5N1|2025 03 16 N1 WM-1st|
A Bean Goose Korea 24WC196 2025 H5N1|2025 02 11| N1 36th|
A Vulture Korea 24WC103 2024 H5N1|2024 12 30| N1 26th|
A Wild Duck 24WF364-8P 2024 H5N1|2024 12 3| N1 12th|
A Eurasian Wigeon 24WF382-4P 2024 H5N1|2024 12 04| N1 14th|
A Eurasian Wigeon Korea 24WF382-7P 2024 H5N1|2024 12 04| N1 14th|
A Wild Duck Korea 24WF653-16P 2025 H5N1|2025 01 14| N1 30th|
A/Hooded crane/Kagoshima/KU-24-42/2024|2024-11-28|H5N1|NA|EPI ISL 19707510
A Mandarin Duck Korea 24WF266-5P 2024 H5N1|2024 11 13| N1 9th|
A Wild Goose Korea 24WF894-1P 2025 H5N1|2025 03 10| N1 40th|
A Wild Duck Korea 24WF159-10P 2024 H5N1|2024 10 28| N1 7th|
A Egret Korea 24WC168 2025 H5N1|2025 01 23| N1 32th|
A Mandarin Duck Korea 24WS069 2025 H5N1|2025 02 13| N1 37th|
A Spot billed Duck Korea 24WS018-1 2024 H5N1|2024 11 12| N1 8th|
A Peregrine Falcon Korea 24WC084 2024 H5N1|2024 12 23| N1 23th|
A Egret Korea 24WC158 2025 H5N1|2025 01 20| N1 31th|
A Egret Korea 24WC160 2025 H5N1|2025 01 20| N1 31th|
A Egret Korea 24WC159 2025 H5N1|2025 01 20| N1 31th|
A/Hooded crane/Kagoshima/KU-24-38/2024|2024-11-27|H5N1|NA|EPI ISL 19707507
A Egret Korea 24WC083 2024 H5N1|2024 12 20| N1 22th|
A Bean Goose Korea 24WC172 2025 H5N1|2025 01 27| N1 33th|
A Egret Korea 24WC128 2025 H5N1|2025 01 10| N1 28th|
A Egret Korea 24WC127 2025 H5N1|2025 01 10| N1 28th|
A Herring Gull Korea 24WC224 2025 H5N1|2025 02 19| N1 38th|
A Egret Korea 24WC070 2024 H5N1|2024 12 16| N1 18th|
A Gray Heron Korea 24WC269 2025 H5N1|2025 03 14| N1 42th|
A Egret Korea 24WC071 2024 H5N1|2024 12 16| N1 18th|
A Egret Korea 24WC142 2025 H5N1|2025 01 14| N1 29th|
A Mandarin Duck Korea 24WF743-8P 2025 H5N1|2025 02 10| N1 35th|
A Egret Korea 24WC176 2025 H5N1|2025 01 31| N1 34th|
A Egret Korea 24WC081 2024 H5N1|2024 12 19| N1 21th|
A Egret Korea 24WC068 2024 H5N1|2024 12 16| N1 18th|
A Egret Korea 24WC069 2024 H5N1|2024 12 16| N1 18th|
A Common Buzzard Korea 24WC267 2025 H5N1|2025 03 14| N1 41th|
A Great Cormorant Korea 24WC275 2025 H5N1|2025 03 24| N1 43th|
A Egret Korea 24WC079 2024 H5N1|2024 12 19| N1 21th|
A Egret Korea 24WC141 2025 H5N1|2025 01 14| N1 29th|
A Egret Korea 24WC140 2025 H5N1|2025 01 14| N1 29th|
A Egret Korea 24WC104 2025 H5N1|2025 01 02| N1 27th|
A/Hooded crane/Kagoshima/KU-24-52/2024|2024-12-02|H5N1|NA|EPI ISL 19707520
A Egret Korea 24WC082 2024 H5N1|2024 12 19| N1 21th|
100
93
74
99
72
95
90
100
100
72
70
93
99
Colored ranges
Highly pathogenic avian influenza
Tree scale: 0.001

G

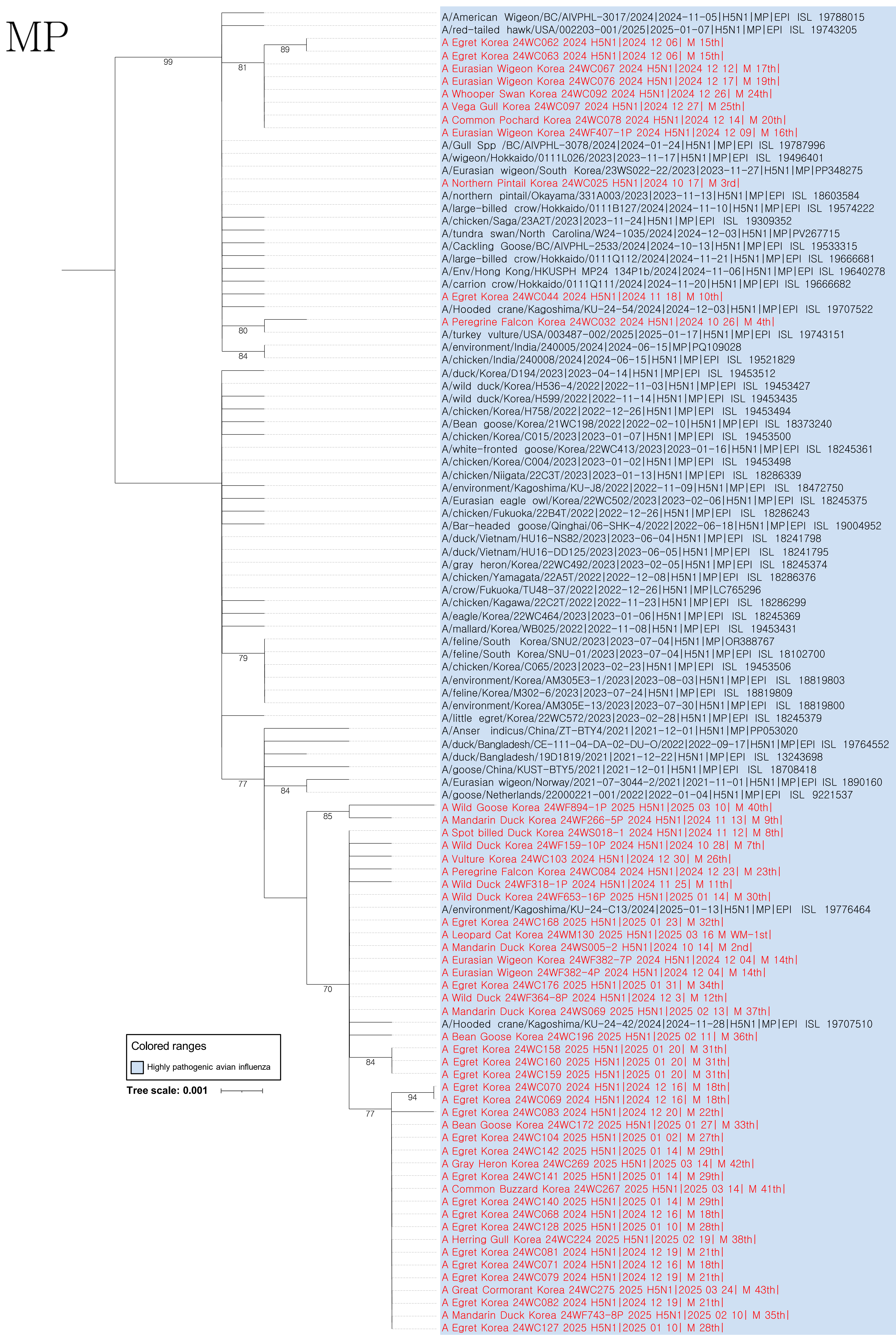

MP
A/American Wigeon/BC/AIVPHL-3017/2024|2024-11-05|H5N1|MP|EPI ISL 19788015
A/red-tailed hawk/USA/002203-001/2025|2025-01-07|H5N1|MP|EPI ISL 19743205
A Egret Korea 24WC062 2024 H5N1|2024 12 06| M 15th|
A Egret Korea 24WC063 2024 H5N1|2024 12 06| M 15th|
A Eurasian Wigeon Korea 24WC067 2024 H5N1|2024 12 12| M 17th|
A Eurasian Wigeon Korea 24WC076 2024 H5N1|2024 12 17| M 19th|
A Whooper Swan Korea 24WC092 2024 H5N1|2024 12 26| M 24th|
A Vega Gull Korea 24WC097 2024 H5N1|2024 12 27| M 25th|
A Common Pochard Korea 24WC078 2024 H5N1|2024 12 14| M 20th|
A Eurasian Wigeon Korea 24WF407-1P 2024 H5N1|2024 12 09| M 16th|
A/Gull Spp /BC/AIVPHL-3078/2024|2024-01-24|H5N1|MP|EPI ISL 19787996
A/wigeon/Hokkaido/0111L026/2023|2023-11-17|H5N1|MP|EPI ISL 19496401
A/Eurasian wigeon/South Korea/23WS022-22/2023|2023-11-27|H5N1|MP|PP348275
A Northern Pintail Korea 24WC025 H5N1|2024 10 17| M 3rd|
A/northern pintail/Okayama/331A003/2023|2023-11-13|H5N1|MP|EPI ISL 18603584
A/large-billed crow/Hokkaido/0111B127/2024|2024-11-10|H5N1|MP|EPI ISL 19574222
A/chicken/Saga/23A2T/2023|2023-11-24|H5N1|MP|EPI ISL 19309352
A/tundra swan/North Carolina/W24-1035/2024|2024-12-03|H5N1|MP|PV267715
A/Cackling Goose/BC/AIVPHL-2533/2024|2024-10-13|H5N1|MP|EPI ISL 19533315
A/large-billed crow/Hokkaido/0111Q112/2024|2024-11-21|H5N1|MP|EPI ISL 19666681
A/Env/Hong Kong/HKUSPH MP24 134P1b/2024|2024-11-06|H5N1|MP|EPI ISL 19640278
A/carrion crow/Hokkaido/0111Q111/2024|2024-11-20|H5N1|MP|EPI ISL 19666682
A Egret Korea 24WC044 2024 H5N1|2024 11 18| M 10th|
A/Hooded crane/Kagoshima/KU-24-54/2024|2024-12-03|H5N1|MP|EPI ISL 19707522
A Peregrine Falcon Korea 24WC032 2024 H5N1|2024 10 26| M 4th|
A/turkey vulture/USA/003487-002/2025|2025-01-17|H5N1|MP|EPI ISL 19743151
A/environment/India/240005/2024|2024-06-15|MP|PQ109028
A/chicken/India/240008/2024|2024-06-15|H5N1|MP|EPI ISL 19521829
A/duck/Korea/D194/2023|2023-04-14|H5N1|MP|EPI ISL 19453512
A/wild duck/Korea/H536-4/2022|2022-11-03|H5N1|MP|EPI ISL 19453427
A/wild duck/Korea/H599/2022|2022-11-14|H5N1|MP|EPI ISL 19453435
A/chicken/Korea/H758/2022|2022-12-26|H5N1|MP|EPI ISL 19453494
A/Bean goose/Korea/21WC198/2022|2022-02-10|H5N1|MP|EPI ISL 18373240
A/chicken/Korea/C015/2023|2023-01-07|H5N1|MP|EPI ISL 19453500
A/white-fronted goose/Korea/22WC413/2023|2023-01-16|H5N1|MP|EPI ISL 18245361
A/chicken/Korea/C004/2023|2023-01-02|H5N1|MP|EPI ISL 19453498
A/chicken/Niigata/22C3T/2023|2023-01-13|H5N1|MP|EPI ISL 18286339
A/environment/Kagoshima/KU-J8/2022|2022-11-09|H5N1|MP|EPI ISL 18472750
A/Eurasian eagle owl/Korea/22WC502/2023|2023-02-06|H5N1|MP|EPI ISL 18245375
A/chicken/Fukuoka/22B4T/2022|2022-12-26|H5N1|MP|EPI ISL 18286243
A/Bar-headed goose/Qinghai/06-SHK-4/2022|2022-06-18|H5N1|MP|EPI ISL 19004952
A/duck/Vietnam/HU16-NS82/2023|2023-06-04|H5N1|MP|EPI ISL 18241798
A/duck/Vietnam/HU16-DD125/2023|2023-06-05|H5N1|MP|EPI ISL 18241795
A/gray heron/Korea/22WC492/2023|2023-02-05|H5N1|MP|EPI ISL 18245374
A/chicken/Yamagata/22A5T/2022|2022-12-08|H5N1|MP|EPI ISL 18286376
A/crow/Fukuoka/TU48-37/2022|2022-12-26|H5N1|MP|LC765296
A/chicken/Kagawa/22C2T/2022|2022-11-23|H5N1|MP|EPI ISL 18286299
A/eagle/Korea/22WC464/2023|2023-01-06|H5N1|MP|EPI ISL 18245369
A/mallard/Korea/WB025/2022|2022-11-08|H5N1|MP|EPI ISL 19453431
A/feline/South Korea/SNU2/2023|2023-07-04|H5N1|MP|OR388767
A/feline/South Korea/SNU-01/2023|2023-07-04|H5N1|MP|EPI ISL 18102700
A/chicken/Korea/C065/2023|2023-02-23|H5N1|MP|EPI ISL 19453506
A/environment/Korea/AM305E3-1/2023|2023-08-03|H5N1|MP|EPI ISL 18819803
A/feline/Korea/M302-6/2023|2023-07-24|H5N1|MP|EPI ISL 18819809
A/environment/Korea/AM305E-13/2023|2023-07-30|H5N1|MP|EPI ISL 18819800
A/little egret/Korea/22WC572/2023|2023-02-28|H5N1|MP|EPI ISL 18245379
A/Anser indicus/China/ZT-BTY4/2021|2021-12-01|H5N1|MP|PP053020
A/duck/Bangladesh/CE-111-04-DA-02-DU-O/2022|2022-09-17|H5N1|MP|EPI ISL 19764552
A/duck/Bangladesh/19D1819/2021|2021-12-22|H5N1|MP|EPI ISL 13243698
A/goose/China/KUST-BTY5/2021|2021-12-01|H5N1|MP|EPI ISL 18708418
A/Eurasian wigeon/Norway/2021-07-3044-2/2021|2021-11-01|H5N1|MP|EPI ISL 1890160
A/goose/Netherlands/22000221-001/2022|2022-01-04|H5N1|MP|EPI ISL 9221537
A Wild Goose Korea 24WF894-1P 2025 H5N1|2025 03 10| M 40th|
A Mandarin Duck Korea 24WF266-5P 2024 H5N1|2024 11 13| M 9th|
A Spot billed Duck Korea 24WS018-1 2024 H5N1|2024 11 12| M 8th|
A Wild Duck Korea 24WF159-10P 2024 H5N1|2024 10 28| M 7th|
A Vulture Korea 24WC103 2024 H5N1|2024 12 30| M 26th|
A Peregrine Falcon Korea 24WC084 2024 H5N1|2024 12 23| M 23th|
A Wild Duck 24WF318-1P 2024 H5N1|2024 11 25| M 11th|
A Wild Duck Korea 24WF653-16P 2025 H5N1|2025 01 14| M 30th|
A/environment/Kagoshima/KU-24-C13/2024|2025-01-13|H5N1|MP|EPI ISL 19776464
A Egret Korea 24WC168 2025 H5N1|2025 01 23| M 32th|
A Leopard Cat Korea 24WM130 2025 H5N1|2025 03 16 M WM-1st|
A Mandarin Duck Korea 24WS005-2 H5N1|2024 10 14| M 2nd|
A Eurasian Wigeon Korea 24WF382-7P 2024 H5N1|2024 12 04| M 14th|
A Eurasian Wigeon 24WF382-4P 2024 H5N1|2024 12 04| M 14th|
A Egret Korea 24WC176 2025 H5N1|2025 01 31| M 34th|
A Wild Duck 24WF364-8P 2024 H5N1|2024 12 3| M 12th|
A Mandarin Duck Korea 24WS069 2025 H5N1|2025 02 13| M 37th|
A/Hooded crane/Kagoshima/KU-24-42/2024|2024-11-28|H5N1|MP|EPI ISL 19707510
A Bean Goose Korea 24WC196 2025 H5N1|2025 02 11| M 36th|
A Egret Korea 24WC158 2025 H5N1|2025 01 20| M 31th|
A Egret Korea 24WC160 2025 H5N1|2025 01 20| M 31th|
A Egret Korea 24WC159 2025 H5N1|2025 01 20| M 31th|
A Egret Korea 24WC070 2024 H5N1|2024 12 16| M 18th|
A Egret Korea 24WC069 2024 H5N1|2024 12 16| M 18th|
A Egret Korea 24WC083 2024 H5N1|2024 12 20| M 22th|
A Bean Goose Korea 24WC172 2025 H5N1|2025 01 27| M 33th|
A Egret Korea 24WC104 2025 H5N1|2025 01 02| M 27th|
A Egret Korea 24WC142 2025 H5N1|2025 01 14| M 29th|
A Gray Heron Korea 24WC269 2025 H5N1|2025 03 14| M 42th|
A Egret Korea 24WC141 2025 H5N1|2025 01 14| M 29th|
A Common Buzzard Korea 24WC267 2025 H5N1|2025 03 14| M 41th|
A Egret Korea 24WC140 2025 H5N1|2025 01 14| M 29th|
A Egret Korea 24WC068 2024 H5N1|2024 12 16| M 18th|
A Egret Korea 24WC128 2025 H5N1|2025 01 10| M 28th|
A Herring Gull Korea 24WC224 2025 H5N1|2025 02 19| M 38th|
A Egret Korea 24WC081 2024 H5N1|2024 12 19| M 21th|
A Egret Korea 24WC071 2024 H5N1|2024 12 16| M 18th|
A Egret Korea 24WC079 2024 H5N1|2024 12 19| M 21th|
A Great Cormorant Korea 24WC275 2025 H5N1|2025 03 24| M 43th|
A Egret Korea 24WC082 2024 H5N1|2024 12 19| M 21th|
A Mandarin Duck Korea 24WF743-8P 2025 H5N1|2025 02 10| M 35th|
A Egret Korea 24WC127 2025 H5N1|2025 01 10| M 28th|
99
81
89
80
84
79
77
84
85
70
84
94
77
Colored ranges
Highly pathogenic avian influenza
Tree scale: 0.001

H

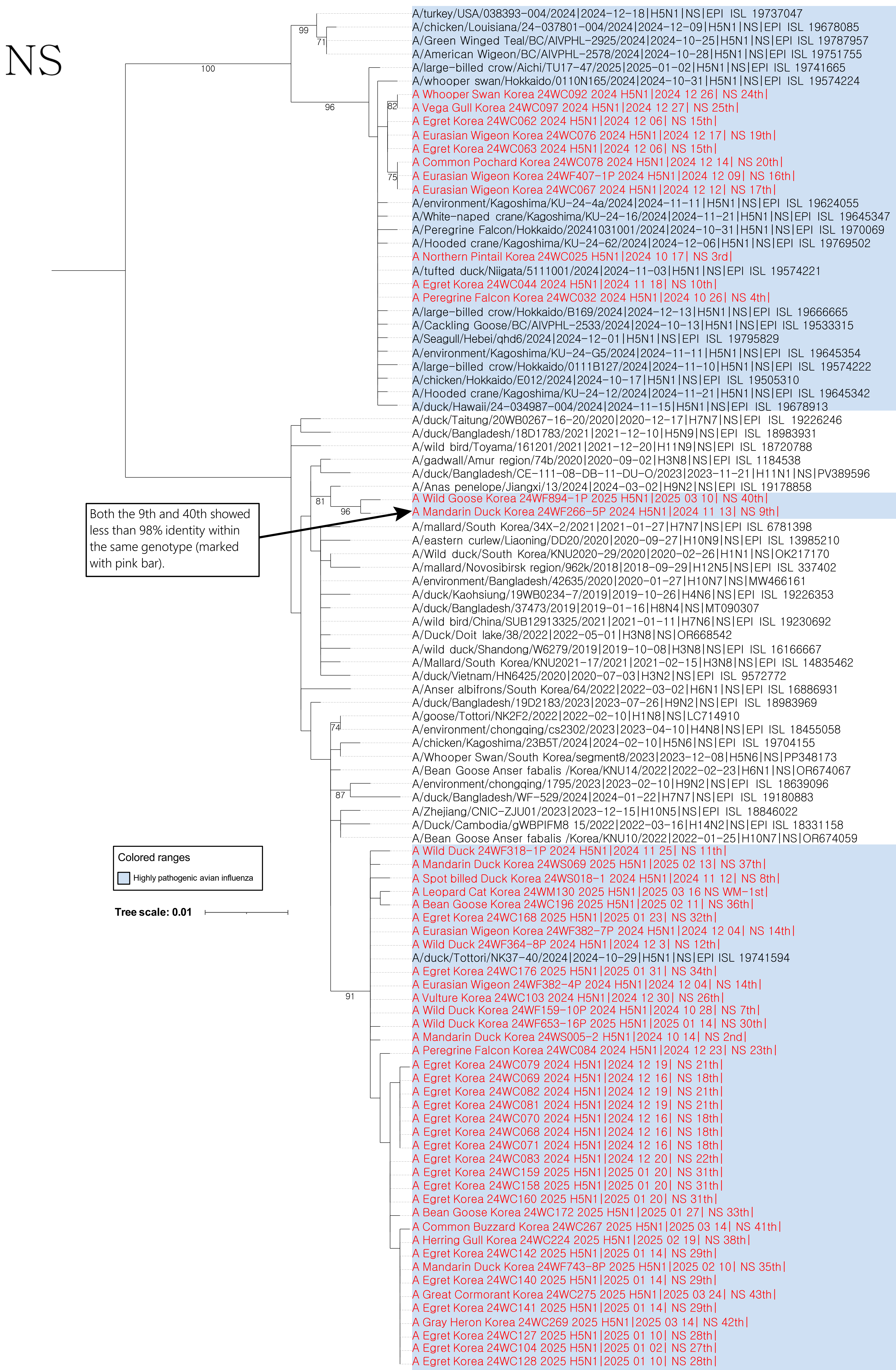

NS
100
99
71
96
82
75
81
96
74
87
91
A/turkey/USA/038393-004/2024|2024-12-18|H5N1|NS|EPI ISL 19737047
A/chicken/Louisiana/24-037801-004/2024|2024-12-09|H5N1|NS|EPI ISL 19678085
A/Green Winged Teal/BC/AIVPHL-2925/2024|2024-10-25|H5N1|NS|EPI ISL 19787957
A/American Wigeon/BC/AIVPHL-2578/2024|2024-10-28|H5N1|NS|EPI ISL 19751755
A/large-billed crow/Aichi/TU17-47/2025|2025-01-02|H5N1|NS|EPI ISL 19741665
A/whooper swan/Hokkaido/0110N165/2024|2024-10-31|H5N1|NS|EPI ISL 19574224
A Whooper Swan Korea 24WC092 2024 H5N1|2024 12 26| NS 24th|
A Vega Gull Korea 24WC097 2024 H5N1|2024 12 27| NS 25th|
A Egret Korea 24WC062 2024 H5N1|2024 12 06| NS 15th|
A Eurasian Wigeon Korea 24WC076 2024 H5N1|2024 12 17| NS 19th|
A Egret Korea 24WC063 2024 H5N1|2024 12 06| NS 15th|
A Common Pochard Korea 24WC078 2024 H5N1|2024 12 14| NS 20th|
A Eurasian Wigeon Korea 24WF407-1P 2024 H5N1|2024 12 09| NS 16th|
A Eurasian Wigeon Korea 24WC067 2024 H5N1|2024 12 12| NS 17th|
A/environment/Kagoshima/KU-24-4a/2024|2024-11-11|H5N1|NS|EPI ISL 19624055
A/White-naped crane/Kagoshima/KU-24-16/2024|2024-11-21|H5N1|NS|EPI ISL 19645347
A/Peregrine Falcon/Hokkaido/20241031001/2024|2024-10-31|H5N1|NS|EPI ISL 1970069
A/Hooded crane/Kagoshima/KU-24-62/2024|2024-12-06|H5N1|NS|EPI ISL 19769502
A Northern Pintail Korea 24WC025 H5N1|2024 10 17| NS 3rd|
A/tufted duck/Niigata/5111001/2024|2024-11-03|H5N1|NS|EPI ISL 19574221
A Egret Korea 24WC044 2024 H5N1|2024 11 18| NS 10th|
A Peregrine Falcon Korea 24WC032 2024 H5N1|2024 10 26| NS 4th|
A/large-billed crow/Hokkaido/B169/2024|2024-12-13|H5N1|NS|EPI ISL 19666665
A/Cackling Goose/BC/AIVPHL-2533/2024|2024-10-13|H5N1|NS|EPI ISL 19533315
A/Seagull/Hebei/qhd6/2024|2024-12-01|H5N1|NS|EPI ISL 19795829
A/environment/Kagoshima/KU-24-G5/2024|2024-11-11|H5N1|NS|EPI ISL 19645354
A/large-billed crow/Hokkaido/0111B127/2024|2024-11-10|H5N1|NS|EPI ISL 19574222
A/chicken/Hokkaido/E012/2024|2024-10-17|H5N1|NS|EPI ISL 19505310
A/Hooded crane/Kagoshima/KU-24-12/2024|2024-11-21|H5N1|NS|EPI ISL 19645342
A/duck/Hawaii/24-034987-004/2024|2024-11-15|H5N1|NS|EPI ISL 19678913
A/duck/Taitung/20WB0267-16-20/2020|2020-12-17|H7N7|NS|EPI ISL 19226246
A/duck/Bangladesh/18D1783/2021|2021-12-10|H5N9|NS|EPI ISL 18983931
A/wild bird/Toyama/161201/2021|2021-12-20|H11N9|NS|EPI ISL 18720788
A/gadwall/Amur region/74b/2020|2020-09-02|H3N8|NS|EPI ISL 1184538
A/duck/Bangladesh/CE-111-08-DB-11-DU-O/2023|2023-11-21|H11N1|NS|PV389596
A/Anas penelope/Jiangxi/13/2024|2024-03-02|H9N2|NS|EPI ISL 19178858
A Wild Goose Korea 24WF894-1P 2025 H5N1|2025 03 10| NS 40th|
A Mandarin Duck Korea 24WF266-5P 2024 H5N1|2024 11 13| NS 9th|
Both the 9th and 40th showed less than 98% identity within the same genotype (marked with pink bar).
A/mallard/South Korea/34X-2/2021|2021-01-27|H7N7|NS|EPI ISL 6781398
A/eastern curlew/Liaoning/DD20/2020|2020-09-27|H10N9|NS|EPI ISL 13985210
A/Wild duck/South Korea/KNU2020-29/2020|2020-02-26|H1N1|NS|OK217170
A/mallard/Novosibirsk region/962k/2018|2018-09-29|H12N5|NS|EPI ISL 337402
A/environment/Bangladesh/42635/2020|2020-01-27|H10N7|NS|MW466161
A/duck/Kaohsiung/19WB0234-7/2019|2019-10-26|H4N6|NS|EPI ISL 19226353
A/duck/Bangladesh/37473/2019|2019-01-16|H8N4|NS|MT090307
A/wild bird/China/SUB12913325/2021|2021-01-11|H7N6|NS|EPI ISL 19230692
A/Duck/Doit lake/38/2022|2022-05-01|H3N8|NS|OR668542
A/wild duck/Shandong/W6279/2019|2019-10-08|H3N8|NS|EPI ISL 16166667
A/Mallard/South Korea/KNU2021-17/2021|2021-02-15|H3N8|NS|EPI ISL 14835462
A/duck/Vietnam/HN6425/2020|2020-07-03|H3N2|NS|EPI ISL 9572772
A/Anser albifrons/South Korea/64/2022|2022-03-02|H6N1|NS|EPI ISL 16886931
A/duck/Bangladesh/19D2183/2023|2023-07-26|H9N2|NS|EPI ISL 18983969
A/goose/Tottori/NK2F2/2022|2022-02-10|H1N8|NS|LC714910
A/environment/chongqing/cs2302/2023|2023-04-10|H4N8|NS|EPI ISL 18455058
A/chicken/Kagoshima/23B5T/2024|2024-02-10|H5N6|NS|EPI ISL 19704155
A/Whooper Swan/South Korea/segment8/2023|2023-12-08|H5N6|NS|PP348173
A/Bean Goose Anser fabalis /Korea/KNU14/2022|2022-02-23|H6N1|NS|OR674067
A/environment/chongqing/1795/2023|2023-02-10|H9N2|NS|EPI ISL 18639096
A/duck/Bangladesh/WF-529/2024|2024-01-22|H7N7|NS|EPI ISL 19180883
A/Zhejiang/CNIC-ZJU01/2023|2023-12-15|H10N5|NS|EPI ISL 18846022
A/Duck/Cambodia/gWBPIFM8 15/2022|2022-03-16|H14N2|NS|EPI ISL 18331158
A/Bean Goose Anser fabalis /Korea/KNU10/2022|2022-01-25|H10N7|NS|OR674059
A Wild Duck 24WF318-1P 2024 H5N1|2024 11 25| NS 11th|
A Mandarin Duck Korea 24WS069 2025 H5N1|2025 02 13| NS 37th|
A Spot billed Duck Korea 24WS018-1 2024 H5N1|2024 11 12| NS 8th|
A Leopard Cat Korea 24WM130 2025 H5N1|2025 03 16 NS WM-1st|
A Bean Goose Korea 24WC196 2025 H5N1|2025 02 11| NS 36th|
A Egret Korea 24WC168 2025 H5N1|2025 01 23| NS 32th|
A Eurasian Wigeon Korea 24WF382-7P 2024 H5N1|2024 12 04| NS 14th|
A Wild Duck 24WF364-8P 2024 H5N1|2024 12 3| NS 12th|
A/duck/Tottori/NK37-40/2024|2024-10-29|H5N1|NS|EPI ISL 19741594
A Egret Korea 24WC176 2025 H5N1|2025 01 31| NS 34th|
A Eurasian Wigeon 24WF382-4P 2024 H5N1|2024 12 04| NS 14th|
A Vulture Korea 24WC103 2024 H5N1|2024 12 30| NS 26th|
A Wild Duck Korea 24WF159-10P 2024 H5N1|2024 10 28| NS 7th|
A Wild Duck Korea 24WF653-16P 2025 H5N1|2025 01 14| NS 30th|
A Mandarin Duck Korea 24WS005-2 H5N1|2024 10 14| NS 2nd|
A Peregrine Falcon Korea 24WC084 2024 H5N1|2024 12 23| NS 23th|
A Egret Korea 24WC079 2024 H5N1|2024 12 19| NS 21th|
A Egret Korea 24WC069 2024 H5N1|2024 12 16| NS 18th|
A Egret Korea 24WC082 2024 H5N1|2024 12 19| NS 21th|
A Egret Korea 24WC081 2024 H5N1|2024 12 19| NS 21th|
A Egret Korea 24WC070 2024 H5N1|2024 12 16| NS 18th|
A Egret Korea 24WC068 2024 H5N1|2024 12 16| NS 18th|
A Egret Korea 24WC071 2024 H5N1|2024 12 16| NS 18th|
A Egret Korea 24WC083 2024 H5N1|2024 12 20| NS 22th|
A Egret Korea 24WC159 2025 H5N1|2025 01 20| NS 31th|
A Egret Korea 24WC158 2025 H5N1|2025 01 20| NS 31th|
A Egret Korea 24WC160 2025 H5N1|2025 01 20| NS 31th|
A Bean Goose Korea 24WC172 2025 H5N1|2025 01 27| NS 33th|
A Common Buzzard Korea 24WC267 2025 H5N1|2025 03 14| NS 41th|
A Herring Gull Korea 24WC224 2025 H5N1|2025 02 19| NS 38th|
A Egret Korea 24WC142 2025 H5N1|2025 01 14| NS 29th|
A Mandarin Duck Korea 24WF743-8P 2025 H5N1|2025 02 10| NS 35th|
A Egret Korea 24WC140 2025 H5N1|2025 01 14| NS 29th|
A Great Cormorant Korea 24WC275 2025 H5N1|2025 03 24| NS 43th|
A Egret Korea 24WC141 2025 H5N1|2025 01 14| NS 29th|
A Gray Heron Korea 24WC269 2025 H5N1|2025 03 14| NS 42th|
A Egret Korea 24WC127 2025 H5N1|2025 01 10| NS 28th|
A Egret Korea 24WC104 2025 H5N1|2025 01 02| NS 27th|
A Egret Korea 24WC128 2025 H5N1|2025 01 10| NS 28th|
Colored ranges
Highly pathogenic avian influenza
Tree scale: 0.01
