# Supplementary material for: Multiple introductions and spread of novel reassortant highly pathogenic avian influenza A (H5N1) clade 2.3.4.4b viruses via wild birds, South Korea, 2024–2025
Source: Vet Res. 2026 Oct 7;57:178. doi: 10.1186/s13567-026-01847-2 (PMC13647676; doi:10.1186/s13567-026-01847-2)
Supplement: Supplementary file 2 [file 13567_2026_1847_MOESM4_ESM.pdf]

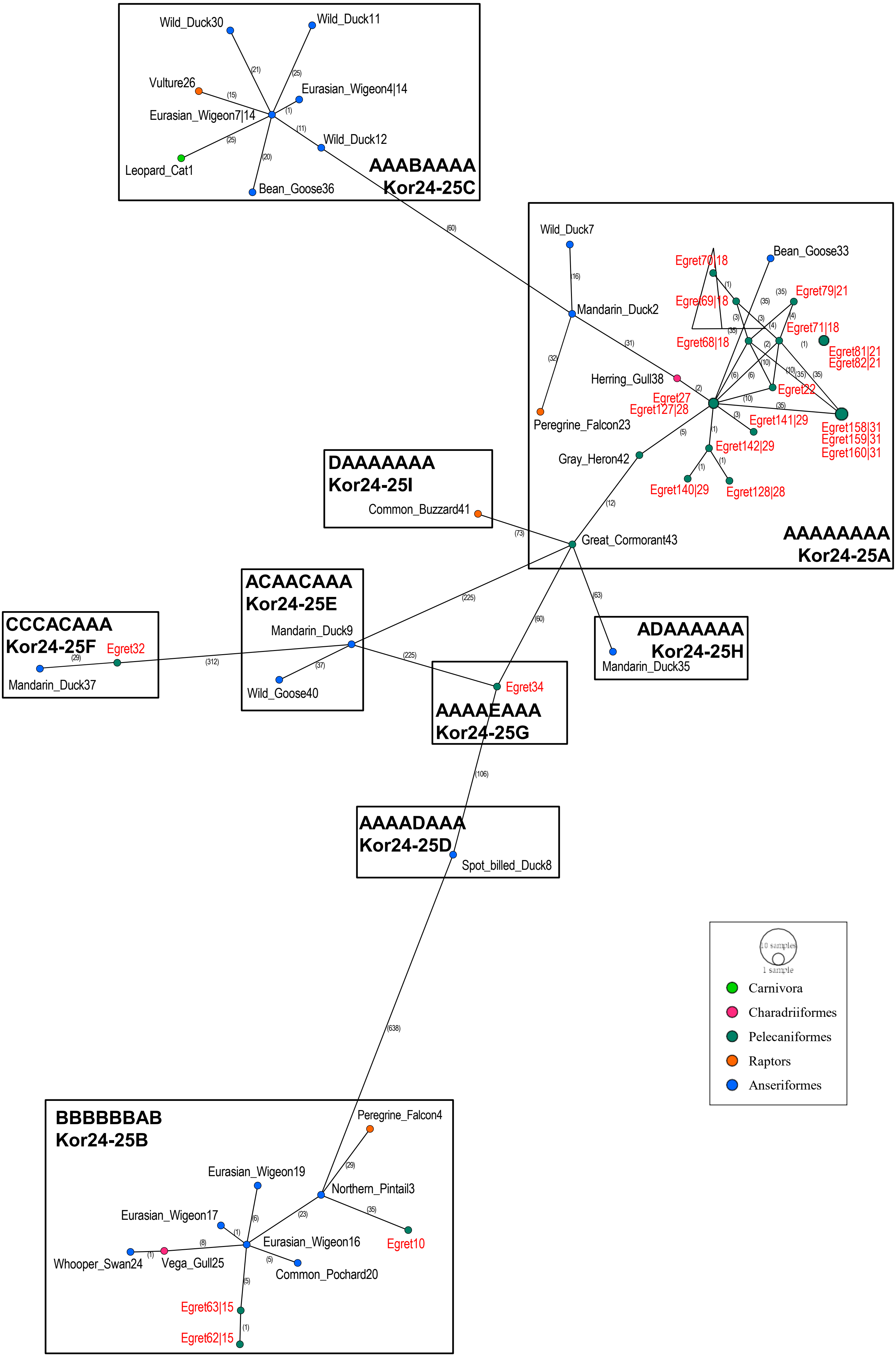

AAABAAAA
Kor24-25C
Wild_Duck30
Wild_Duck11
Vulture26
Eurasian_Wigeon4|14
Eurasian_Wigeon7|14
Wild_Duck12
Leopard_Cat1
Bean_Goose36
AAAAAAAA
Kor24-25A
Wild_Duck7
Bean_Goose33
Egret70|18
Egret69|18
Egret79|21
Mandarin_Duck2
Egret71|18
Egret68|18
Egret81|21
Egret82|21
Herring_Gull38
Egret22
Egret27
Egret127|28
Peregrine_Falcon23
Egret141|29
Egret158|31
Egret159|31
Egret160|31
Gray_Heron42
Egret142|29
Egret140|29
Egret128|28
Great_Cormorant43
DAAAAAAA
Kor24-25I
Common_Buzzard41
ACAACAAA
Kor24-25E
Mandarin_Duck9
Wild_Goose40
CCCACAAA
Kor24-25F
Egret32
Mandarin_Duck37
ADAAAAAA
Kor24-25H
Mandarin_Duck35
Egret34
AAAAEAAA
Kor24-25G
AAAADAAA
Kor24-25D
Spot_billed_Duck8
BBBBBBAB
Kor24-25B
Peregrine_Falcon4
Eurasian_Wigeon19
Northern_Pintail3
Eurasian_Wigeon17
Eurasian_Wigeon16
Egret10
Whooper_Swan24
Vega_Gull25
Common_Pochard20
Egret63|15
Egret62|15
10 samples
1 sample
Carnivora
Charadriiformes
Pelecaniformes
Raptors
Anseriformes
