# Supplementary material for: Multiple introductions and spread of novel reassortant highly pathogenic avian influenza A (H5N1) clade 2.3.4.4b viruses via wild birds, South Korea, 2024–2025
Source: Vet Res. 2026 Oct 7;57:178. doi: 10.1186/s13567-026-01847-2 (PMC13647676; doi:10.1186/s13567-026-01847-2)
Supplement: Supplementary file 3 [file 13567_2026_1847_MOESM6_ESM.pdf]

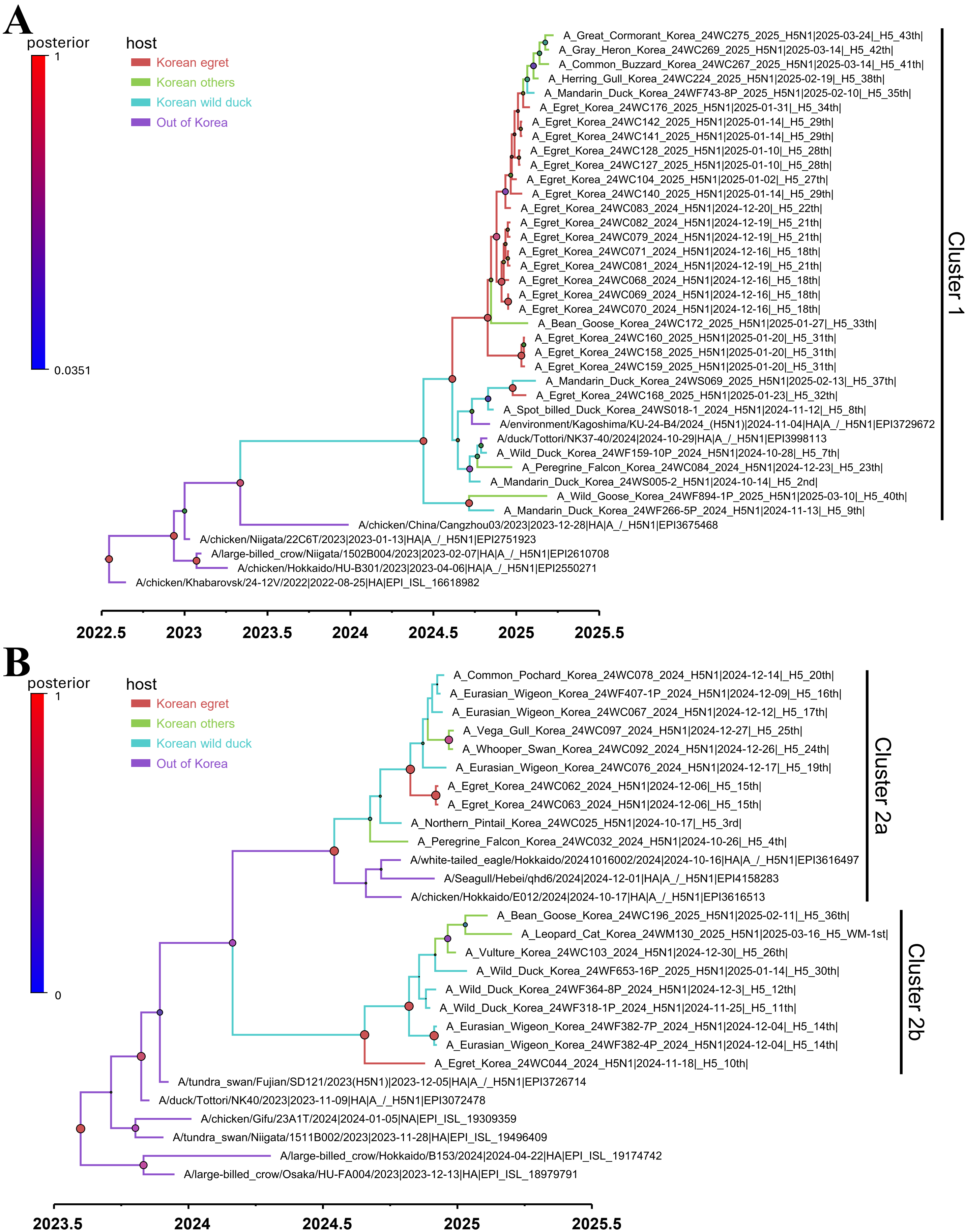

A
posterior
1
0.0351
host
Korean egret
Korean others
Korean wild duck
Out of Korea
A_Great_Cormorant_Korea_24WC275_2025_H5N1|2025-03-24|_H5_43th|
A_Gray_Heron_Korea_24WC269_2025_H5N1|2025-03-14|_H5_42th|
A_Common_Buzzard_Korea_24WC267_2025_H5N1|2025-03-14|_H5_41th|
A_Herring_Gull_Korea_24WC224_2025_H5N1|2025-02-19|_H5_38th|
A_Mandarin_Duck_Korea_24WF743-8P_2025_H5N1|2025-02-10|_H5_35th|
A_Egret_Korea_24WC176_2025_H5N1|2025-01-31|_H5_34th|
A_Egret_Korea_24WC142_2025_H5N1|2025-01-14|_H5_29th|
A_Egret_Korea_24WC141_2025_H5N1|2025-01-14|_H5_29th|
A_Egret_Korea_24WC128_2025_H5N1|2025-01-10|_H5_28th|
A_Egret_Korea_24WC127_2025_H5N1|2025-01-10|_H5_28th|
A_Egret_Korea_24WC104_2025_H5N1|2025-01-02|_H5_27th|
A_Egret_Korea_24WC140_2025_H5N1|2025-01-14|_H5_29th|
A_Egret_Korea_24WC083_2024_H5N1|2024-12-20|_H5_22th|
A_Egret_Korea_24WC082_2024_H5N1|2024-12-19|_H5_21th|
A_Egret_Korea_24WC079_2024_H5N1|2024-12-19|_H5_21th|
A_Egret_Korea_24WC071_2024_H5N1|2024-12-16|_H5_18th|
A_Egret_Korea_24WC081_2024_H5N1|2024-12-19|_H5_21th|
A_Egret_Korea_24WC068_2024_H5N1|2024-12-16|_H5_18th|
A_Egret_Korea_24WC069_2024_H5N1|2024-12-16|_H5_18th|
A_Egret_Korea_24WC070_2024_H5N1|2024-12-16|_H5_18th|
A_Bean_Goose_Korea_24WC172_2025_H5N1|2025-01-27|_H5_33th|
A_Egret_Korea_24WC160_2025_H5N1|2025-01-20|_H5_31th|
A_Egret_Korea_24WC158_2025_H5N1|2025-01-20|_H5_31th|
A_Egret_Korea_24WC159_2025_H5N1|2025-01-20|_H5_31th|
A_Mandarin_Duck_Korea_24WS069_2025_H5N1|2025-02-13|_H5_37th|
A_Egret_Korea_24WC168_2025_H5N1|2025-01-23|_H5_32th|
A_Spot_billed_Duck_Korea_24WS018-1_2024_H5N1|2024-11-12|_H5_8th|
A/environment/Kagoshima/KU-24-B4/2024_(H5N1)|2024-11-04|HA|A_/_H5N1|EPI3729672
A/duck/Tottori/NK37-40/2024|2024-10-29|HA|A_/_H5N1|EPI3998113
A_Wild_Duck_Korea_24WF159-10P_2024_H5N1|2024-10-28|_H5_7th|
A_Peregrine_Falcon_Korea_24WC084_2024_H5N1|2024-12-23|_H5_23th|
A_Mandarin_Duck_Korea_24WS005-2_H5N1|2024-10-14|_H5_2nd|
A_Wild_Goose_Korea_24WF894-1P_2025_H5N1|2025-03-10|_H5_40th|
A_Mandarin_Duck_Korea_24WF266-5P_2024_H5N1|2024-11-13|_H5_9th|
A/chicken/China/Cangzhou03/2023|2023-12-28|HA|A_/_H5N1|EPI3675468
A/chicken/Niigata/22C6T/2023|2023-01-13|HA|A_/_H5N1|EPI2751923
A/large-billed_crow/Niigata/1502B004/2023|2023-02-07|HA|A_/_H5N1|EPI2610708
A/chicken/Hokkaido/HU-B301/2023|2023-04-06|HA|A_/_H5N1|EPI2550271
A/chicken/Khabarovsk/24-12V/2022|2022-08-25|HA|EPI_ISL_16618982
Cluster 1
2022.5
2023
2023.5
2024
2024.5
2025
2025.5
B
posterior
1
0
host
Korean egret
Korean others
Korean wild duck
Out of Korea
A_Common_Pochard_Korea_24WC078_2024_H5N1|2024-12-14|_H5_20th|
A_Eurasian_Wigeon_Korea_24WF407-1P_2024_H5N1|2024-12-09|_H5_16th|
A_Eurasian_Wigeon_Korea_24WC067_2024_H5N1|2024-12-12|_H5_17th|
A_Vega_Gull_Korea_24WC097_2024_H5N1|2024-12-27|_H5_25th|
A_Whooper_Swan_Korea_24WC092_2024_H5N1|2024-12-26|_H5_24th|
A_Eurasian_Wigeon_Korea_24WC076_2024_H5N1|2024-12-17|_H5_19th|
A_Egret_Korea_24WC062_2024_H5N1|2024-12-06|_H5_15th|
A_Egret_Korea_24WC063_2024_H5N1|2024-12-06|_H5_15th|
A_Northern_Pintail_Korea_24WC025_H5N1|2024-10-17|_H5_3rd|
A_Peregrine_Falcon_Korea_24WC032_2024_H5N1|2024-10-26|_H5_4th|
A/white-tailed_eagle/Hokkaido/20241016002/2024|2024-10-16|HA|A_/_H5N1|EPI3616497
A/Seagull/Hebei/qhd6/2024|2024-12-01|HA|A_/_H5N1|EPI4158283
A/chicken/Hokkaido/E012/2024|2024-10-17|HA|A_/_H5N1|EPI3616513
Cluster 2a
A_Bean_Goose_Korea_24WC196_2025_H5N1|2025-02-11|_H5_36th|
A_Leopard_Cat_Korea_24WM130_2025_H5N1|2025-03-16_H5_WM-1st|
A_Vulture_Korea_24WC103_2024_H5N1|2024-12-30|_H5_26th|
A_Wild_Duck_Korea_24WF653-16P_2025_H5N1|2025-01-14|_H5_30th|
A_Wild_Duck_Korea_24WF364-8P_2024_H5N1|2024-12-3|_H5_12th|
A_Wild_Duck_Korea_24WF318-1P_2024_H5N1|2024-11-25|_H5_11th|
A_Eurasian_Wigeon_Korea_24WF382-7P_2024_H5N1|2024-12-04|_H5_14th|
A_Eurasian_Wigeon_Korea_24WF382-4P_2024_H5N1|2024-12-04|_H5_14th|
A_Egret_Korea_24WC044_2024_H5N1|2024-11-18|_H5_10th|
Cluster 2b
A/tundra_swan/Fujian/SD121/2023(H5N1)|2023-12-05|HA|A_/_H5N1|EPI3726714
A/duck/Tottori/NK40/2023|2023-11-09|HA|A_/_H5N1|EPI3072478
A/chicken/Gifu/23A1T/2024|2024-01-05|NA|EPI_ISL_19309359
A/tundra_swan/Niigata/1511B002/2023|2023-11-28|HA|EPI_ISL_19496409
A/large-billed_crow/Hokkaido/B153/2024|2024-04-22|HA|EPI_ISL_19174742
A/large-billed_crow/Osaka/HU-FA004/2023|2023-12-13|HA|EPI_ISL_18979791
2023.5
2024
2024.5
2025
2025.5
