# Supplementary material for: Multiple introductions and spread of novel reassortant highly pathogenic avian influenza A (H5N1) clade 2.3.4.4b viruses via wild birds, South Korea, 2024–2025
Source: Vet Res. 2026 Oct 7;57:178. doi: 10.1186/s13567-026-01847-2 (PMC13647676; doi:10.1186/s13567-026-01847-2)
Supplement: Supplementary file 4 [file 13567_2026_1847_MOESM7_ESM.pdf]

A

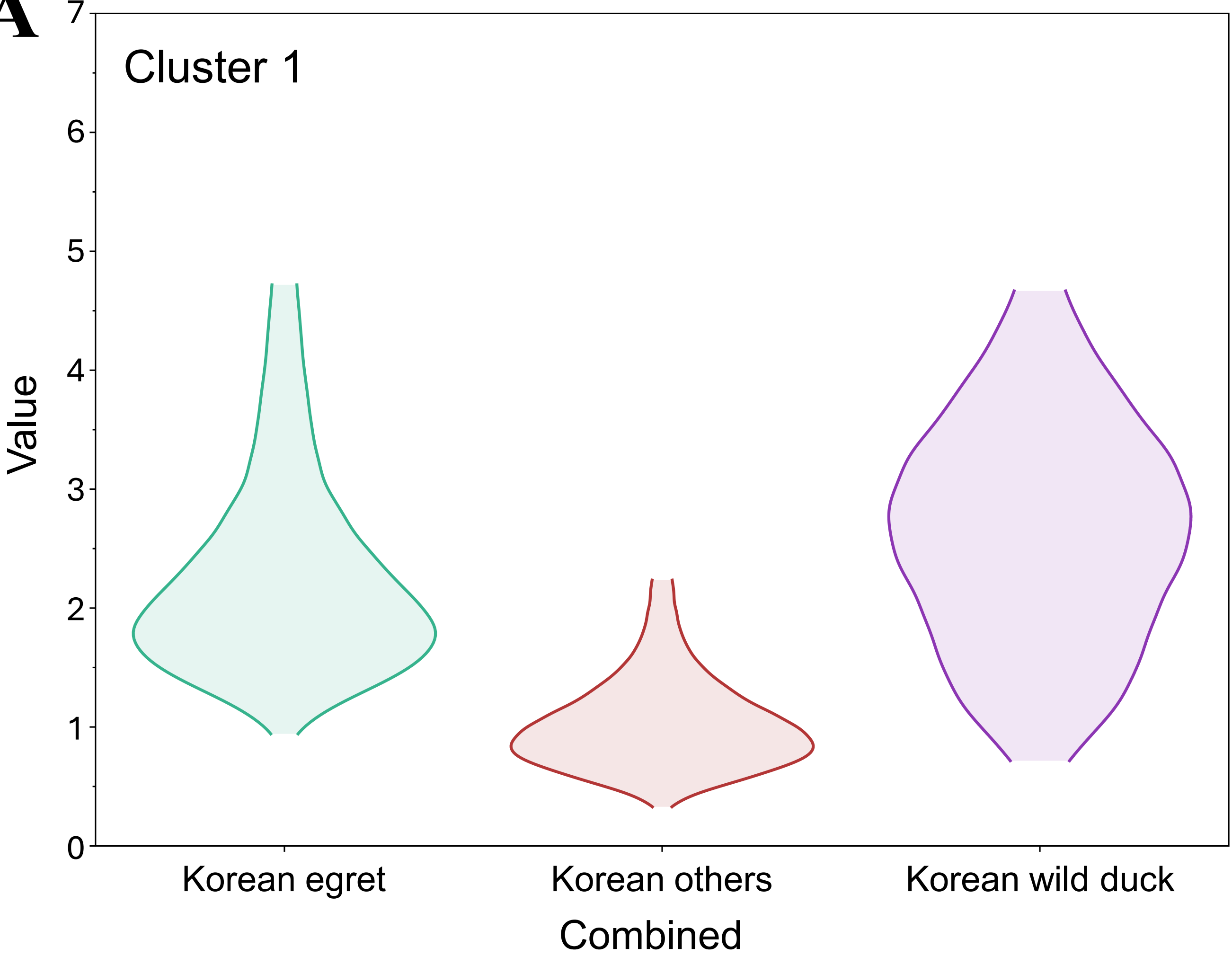
Cluster 1
7
6
5
4
3
2
1
0
Value
Korean egret
Korean others
Korean wild duck
Combined

B

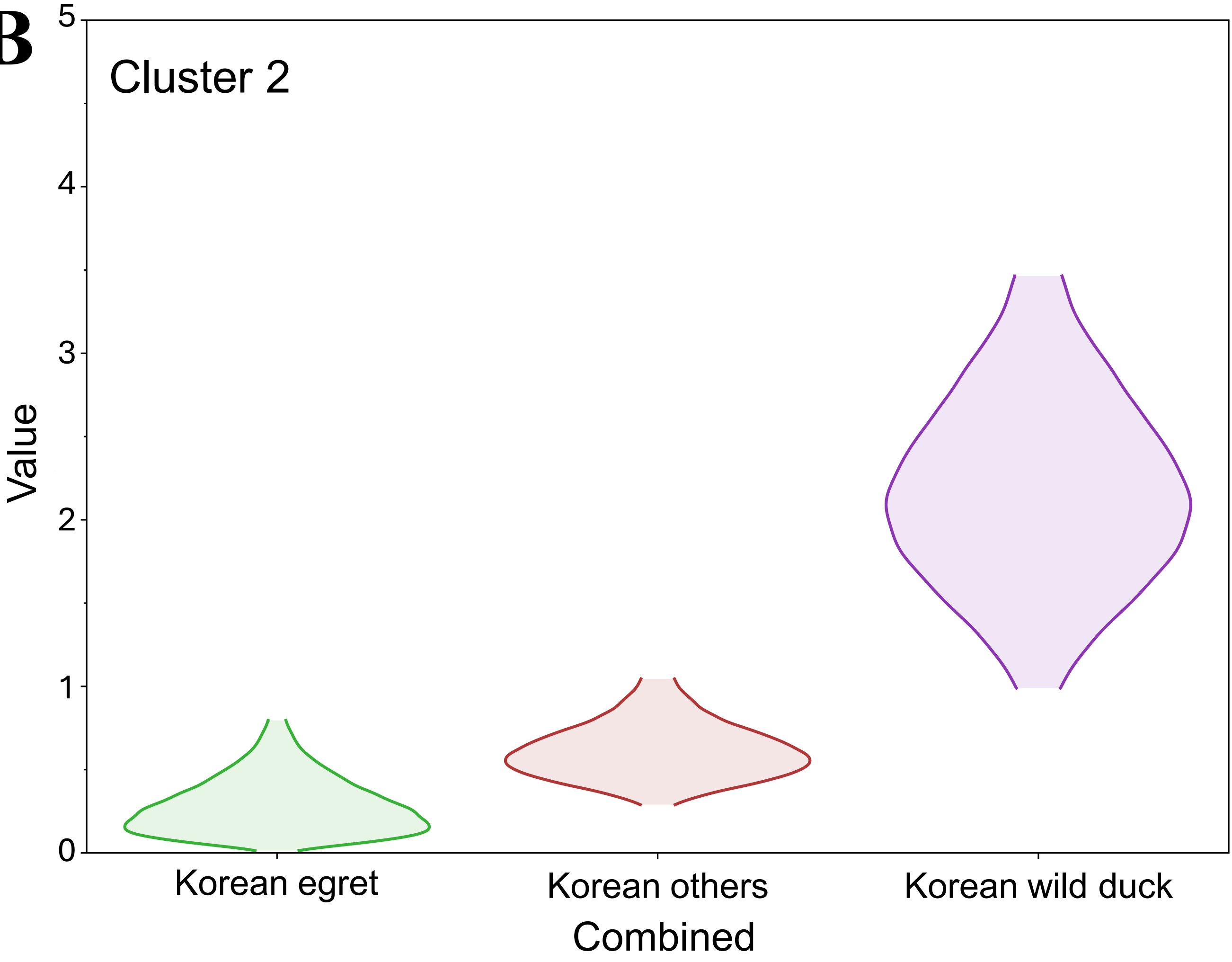
Cluster 2
5
4
3
2
1
0
Value
Korean egret
Korean others
Korean wild duck
Combined
