# Supplementary material for: Multiple introductions and spread of novel reassortant highly pathogenic avian influenza A (H5N1) clade 2.3.4.4b viruses via wild birds, South Korea, 2024–2025
Source: Vet Res. 2026 Oct 7;57:178. doi: 10.1186/s13567-026-01847-2 (PMC13647676; doi:10.1186/s13567-026-01847-2)
Supplement: Supplementary file 5 [file 13567_2026_1847_MOESM1_ESM.docx]

**Additional file 1.** Detailed information on HPAI A(H5N1) virus isolates from wild animals in South Korea, October 2024–March 2025.

| No. | Collection date | Sample ID | Region | Sample type | Host species | Subtype | GISAID Isolate ID |
| --- | --- | --- | --- | --- | --- | --- | --- |
| 2 | 2024-10-14 | 24WS005-2 | Gyeonggi-do | Captured | Mandarin Duck | H5N1 | EPI_ISL_19528860 |
| 3 | 2024-10-17 | 24WC025 | Jeju island | Carcass | Northern Pintail | H5N1 | EPI_ISL_19531393 |
| 4 | 2024-10-26 | 24WC032 | Gyeongsangnam-do | Carcass | Peregrine Falcon | H5N1 | EPI_ISL_20049555 |
| 7 | 2024-10-28 | 24WF159-10P | Gyeonggi-do | Feces | Wild Duck | H5N1 | EPI_ISL_20049556 |
| 8 | 2024-11-12 | 24WS018-1 | Gyeonggi-do | Captured | Spot billed Duck | H5N1 | EPI_ISL_20049557 |
| 9 | 2024-11-13 | 24WF266-5P | Gyeonggi-do | Feces | Mandarin Duck | H5N1 | EPI_ISL_20049558 |
| 10 | 2024-11-18 | 24WC044 | Gangwon-do | Carcass | Egret | H5N1 | EPI_ISL_20049559 |
| 11 | 2024-11-25 | 24WF318-1P | Gyeongsangnam-do | Feces | Wild Duck | H5N1 | EPI_ISL_20049560 |
| 12 | 2024-12-03 | 24WF364-8P | Chungcheongbuk-do | Feces | Wild Duck | H5N1 | EPI_ISL_20051150 |
| 14 | 2024-12-04 | 24WF382-4P 24WF382-7P | Jeju island | Feces | Eurasian Wigeon | H5N1 | EPI_ISL_19832581  EPI_ISL_20049561 |
| 15 | 2024-12-06 | 24WC062 24WC063 | Jeollabuk-do | Carcass | Egret | H5N1 | EPI_ISL_20049562  EPI_ISL_20049563 |
| 16 | 2024-12-09 | 24WF407-1P | Jeju island | Feces | Eurasian Wigeon | H5N1 | EPI_ISL_20049564 |
| 17 | 2024-12-12 | 24WC067 | Jeju island | Carcass | Eurasian Wigeon | H5N1 | EPI_ISL_20049565 |
| 20 | 2024-12-14 | 24WC078 | Jeju island | Carcass | Common Pochard | H5N1 | EPI_ISL_20049571 |
| 18 | 2024-12-16 | 24WC068 24WC069 24WC070 24WC071 | Chungcheongnam-do | Carcass | Egret | H5N1 | EPI_ISL_20049566  EPI_ISL_20049567  EPI_ISL_20049568  EPI_ISL_20049569 |
| 19 | 2024-12-17 | 24WC076 | Jeju island | Carcass | Eurasian Wigeon | H5N1 | EPI_ISL_20049570 |
| 21 | 2024-12-19 | 24WC079 24WC081 24WC082 | Chungcheongnam-do | Carcass | Egret | H5N1 | EPI_ISL_20049573  EPI_ISL_20049574  EPI_ISL_20049572 |
| 22 | 2024-12-20 | 24WC083 | Chungcheongnam-do | Carcass | Egret | H5N1 | EPI_ISL_20049575 |
| 23 | 2024-12-23 | 24WC084 | Jeollabuk-do | Carcass | Peregrine Falcon | H5N1 | EPI_ISL_20049576 |
| 24 | 2024-12-26 | 24WC092 | Gyeongsangbuk-do | Carcass | Whooper Swan | H5N1 | EPI_ISL_20049577 |
| 25 | 2024-12-27 | 24WC097 | Jeju island | Carcass | Vega Gull | H5N1 | EPI_ISL_20049578 |
| 26 | 2024-12-30 | 24WC103 | Chungcheongnam-do | Carcass | Vulture | H5N1 | EPI_ISL_19832582 |
| 27 | 2025-01-02 | 24WC104 | Chungcheongnam-do | Carcass | Egret | H5N1 | EPI_ISL_20049579 |
| 28 | 2025-01-10 | 24WC127 24WC128 | Chungcheongnam-do | Carcass | Egret | H5N1 | EPI_ISL_20049580  EPI_ISL_20049581 |
| 29 | 2025-01-14 | 24WC140 24WC141 24WC142 | Chungcheongnam-do | Carcass | Egret | H5N1 | EPI_ISL_20049582  EPI_ISL_20049583  EPI_ISL_20049584 |
| 30 | 2025-01-14 | 24WF653-16P | Gyeonggi-do | Feces | Wild Duck | H5N1 | EPI_ISL_20049585 |
| 31 | 2025-01-20 | 24WC158 24WC159 24WC160 | Gyeongsangbuk-do | Carcass | Egret | H5N1 | EPI_ISL_20049586  EPI_ISL_20049587  EPI_ISL_20049588 |
| 32 | 2025-01-23 | 24WC168 | Gyeonggi-do | Carcass | Egret | H5N1 | EPI_ISL_20049589 |
| 33 | 2025-01-27 | 24WC172 | Gyeongsangnam-do | Carcass | Bean Goose | H5N1 | EPI_ISL_20049590 |
| 34 | 2025-01-31 | 24WC176 | Gyeonggi-do | Carcass | Egret | H5N1 | EPI_ISL_20049591 |
| 35 | 2025-02-10 | 24WF743-8P | Chungcheongnam-do | Feces | Mandarin Duck | H5N1 | EPI_ISL_20049592 |
| 36 | 2025-02-11 | 24WC196 | Chungcheongnam-do | Carcass | Bean Goose | H5N1 | EPI_ISL_19832583 |
| 37 | 2025-02-13 | 24WS069 | Gyeonggi-do | Captured | Mandarin Duck | H5N1 | EPI_ISL_20049593 |
| 38 | 2025-02-19 | 24WC224 | Chungcheongnam-do | Carcass | Herring Gull | H5N1 | EPI_ISL_20049594 |
| 40 | 2025-03-10 | 24WF894-1P | Gyeongsangnam-do | Feces | Wild Goose | H5N1 | EPI_ISL_20049595 |
| 41 | 2025-03-14 | 24WC267 | Incheon | Carcass | Common Buzzard | H5N1 | EPI_ISL_20049596 |
| 42 | 2025-03-14 | 24WC269 | Jeollanam-do | Carcass | Gray heron | H5N1 | EPI_ISL_20049597 |
|  | 2025-03-16 | 24WM130 | Jeollanam-do | Carcass | Leopard Cat | H5N1 | EPI_ISL_20051149 |
| 43 | 2025-03-24 | 24WC275 | Gyeonggi-do | Carcass | Great Cormorant | H5N1 | EPI_ISL_20049598 |
