# Supplementary material for: Multiple introductions and spread of novel reassortant highly pathogenic avian influenza A (H5N1) clade 2.3.4.4b viruses via wild birds, South Korea, 2024–2025
Source: Vet Res. 2026 Oct 7;57:178. doi: 10.1186/s13567-026-01847-2 (PMC13647676; doi:10.1186/s13567-026-01847-2)
Supplement: Supplementary file 6 [file 13567_2026_1847_MOESM2_ESM.docx]

**Additional file 2**. Composition of maximum clade credibility (MCC) tree datasets used for phylogenetic and discrete trait analyses of HPAI H5N1 viruses.

| **Dataset** | **Trait** | **Strain** | **GISAID isolate ID** |
| --- | --- | --- | --- |
| H5N1 cluster 1  (n=39) | Korean  wild duck  (n=6) | A/Mandarin Duck/Korea/24WS069/2025_H5N1 | EPI_ISL_20049593 |
|  |  | A/Spot billed Duck/Korea/24WS018-1/2024_H5N1 | EPI_ISL_20049557 |
|  |  | A/Mandarin Duck/Korea/24WF266-5P/2024_H5N1 | EPI_ISL_20049558 |
|  |  | A/Mandarin Duck/Korea/24WF743-8P/2025_H5N1 | EPI_ISL_20049592 |
|  |  | A/Wild Duck/Korea/24WF159-10P/2024_H5N1 | EPI_ISL_20049556 |
|  |  | A/Mandarin Duck/Korea/24WS005-2_H5N1 | EPI_ISL_19528860 |
|  | Korean egret  (n=19) | A/Egret/Korea/24WC069/2024_H5N1 | EPI_ISL_20049567 |
|  |  | A/Egret/Korea/24WC168/2025_H5N1 | EPI_ISL_20049589 |
|  |  | A/Egret/Korea/24WC141/2025_H5N1 | EPI_ISL_20049583 |
|  |  | A/Egret/Korea/24WC128/2025_H5N1 | EPI_ISL_20049581 |
|  |  | A/Egret/Korea/24WC142/2025_H5N1 | EPI_ISL_20049584 |
|  |  | A/Egret/Korea/24WC127/2025_H5N1 | EPI_ISL_20049580 |
|  |  | A/Egret/Korea/24WC104/2025_H5N1 | EPI_ISL_20049579 |
|  |  | A/Egret/Korea/24WC176/2025_H5N1 | EPI_ISL_20049591 |
|  |  | A/Egret/Korea/24WC083/2024_H5N1 | EPI_ISL_20049575 |
|  |  | A/Egret/Korea/24WC140/2025_H5N1 | EPI_ISL_20049582 |
|  |  | A/Egret/Korea/24WC071/2024_H5N1 | EPI_ISL_20049569 |
|  |  | A/Egret/Korea/24WC082/2024_H5N1 | EPI_ISL_20049572 |
|  |  | A/Egret/Korea/24WC081/2024_H5N1 | EPI_ISL_20049574 |
|  |  | A/Egret/Korea/24WC068/2024_H5N1 | EPI_ISL_20049566 |
|  |  | A/Egret/Korea/24WC070/2024_H5N1 | EPI_ISL_20049568 |
|  |  | A/Egret/Korea/24WC079/2024_H5N1 | EPI_ISL_20049573 |
|  |  | A/Egret/Korea/24WC160/2025_H5N1 | EPI_ISL_20049588 |
|  |  | A/Egret/Korea/24WC159/2025_H5N1 | EPI_ISL_20049587 |
|  |  | A/Egret/Korea/24WC158/2025_H5N1 | EPI_ISL_20049586 |
|  | Korean others  (n=7) | A/Wild Goose/Korea/24WF894-1P/2025_H5N1 | EPI_ISL_20049595 |
|  |  | A/Bean Goose/Korea/24WC172/2025_H5N1 | EPI_ISL_20049590 |
|  |  | A/Great Cormorant/Korea/24WC275/2025_H5N1 | EPI_ISL_20049598 |
|  |  | A/Herring Gull/Korea/24WC224/2025_H5N1 | EPI_ISL_20049594 |
|  |  | A/Gray Heron/Korea/24WC269/2025_H5N1 | EPI_ISL_20049597 |
|  |  | A/Common Buzzard/Korea/24WC267/2025_H5N1 | EPI_ISL_20049596 |
|  |  | A/Peregrine Falcon/Korea/24WC084/2024_H5N1 | EPI_ISL_20049576 |
|  | Out of Korea  (n=7) | A/chicken/Niigata/22C6T/2023 | EPI_ISL_18286340 |
|  |  | A/large-billed crow/Niigata/1502B004/2023 | EPI_ISL_17949986 |
|  |  | A/chicken/Hokkaido/HU-B301/2023 | EPI_ISL_17638448 |
|  |  | A/chicken/China/Cangzhou03/2023 | EPI_ISL_19592785 |
|  |  | A/duck/Tottori/NK37-40/2024 | EPI_ISL_19741594 |
|  |  | A/environment/Kagoshima/KU-24-B4/2024_(H5N1) | EPI_ISL_19624046 |
|  |  | A/chicken/Khabarovsk/24-12V/2022 | EPI_ISL_16618982 |
| H5N1 cluster 2  (n=28) | Korean  wild duck  (n=10) | A/Eurasian Wigeon/Korea/24WF407-1P/2024_H5N1 | EPI_ISL_20049564 |
|  |  | A/Common Pochard/Korea/24WC078/2024_H5N1 | EPI_ISL_20049571 |
|  |  | A/Eurasian Wigeon/Korea/24WC067/2024_H5N1 | EPI_ISL_20049565 |
|  |  | A/Eurasian Wigeon/Korea/24WC076/2024_H5N1 | EPI_ISL_20049570 |
|  |  | A/Northern Pintail/Korea/24WC025_H5N1 | EPI_ISL_19531393 |
|  |  | A/Wild Duck/Korea/24WF318-1P/2024_H5N1 | EPI_ISL_20049560 |
|  |  | A/Wild Duck/Korea/24WF364-8P/2024_H5N1 | EPI_ISL_20051150 |
|  |  | A/Eurasian Wigeon/Korea/24WF382-7P/2024_H5N1 | EPI_ISL_20049561 |
|  |  | A/Eurasian Wigeon/Korea/24WF382-4P/2024_H5N1 | EPI_ISL_19832581 |
|  |  | A/Wild Duck/Korea/24WF653-16P/2025_H5N1 | EPI_ISL_20049585 |
|  | Korean egret  (n=3) | A/Egret/Korea/24WC044/2024_H5N1 | EPI_ISL_20049559 |
|  |  | A/Egret/Korea/24WC062/2024_H5N1 | EPI_ISL_20049562 |
|  |  | A/Egret/Korea/24WC063/2024_H5N1 | EPI_ISL_20049563 |
|  | Korean others  (n=6) | A/Whooper Swan/Korea/24WC092/2024_H5N1 | EPI_ISL_20049577 |
|  |  | A/Vega Gull/Korea/24WC097/2024_H5N1 | EPI_ISL_20049578 |
|  |  | A/Peregrine Falcon/Korea/24WC032/2024_H5N1 | EPI_ISL_20049555 |
|  |  | A/Bean Goose/Korea/24WC196/2025_H5N1 | EPI_ISL_19832583 |
|  |  | A/Leopard Cat/Korea/24WM130/2025_H5N1 | EPI_ISL_20051149 |
|  |  | A/Vulture/Korea/24WC103/2024_H5N1 | EPI_ISL_19832582 |
|  | Out of Korea  (n=9) | A/white-tailed_eagle/Hokkaido/20241016002/2024 | EPI_ISL_19504792 |
|  |  | A/chicken/Hokkaido/E012/2024 | EPI_ISL_19505310 |
|  |  | A/Seagull/Hebei/qhd6/2024 | EPI_ISL_19795829 |
|  |  | A/tundra_swan/Fujian/SD121/2023(H5N1) | EPI_ISL_19616578 |
|  |  | A/duck/Tottori/NK40/2023 | EPI_ISL_18935784 |
|  |  | A/tundra_swan/Niigata/1511B002/2023 | EPI_ISL_19496409 |
|  |  | A/large-billed_crow/Hokkaido/B153/2024 | EPI_ISL_19174742 |
|  |  | A/large-billed_crow/Osaka/HU-FA004/2023 | EPI_ISL_18979791 |
|  |  | A/chicken/Gifu/23A1T/2024 | EPI_ISL_19309359 |
