# Supplementary material for: Multiple introductions and spread of novel reassortant highly pathogenic avian influenza A (H5N1) clade 2.3.4.4b viruses via wild birds, South Korea, 2024–2025
Source: Vet Res. 2026 Oct 7;57:178. doi: 10.1186/s13567-026-01847-2 (PMC13647676; doi:10.1186/s13567-026-01847-2)
Supplement: Supplementary file 7 [file 13567_2026_1847_MOESM5_ESM.docx]

**Additional file 5**. FluMut-based mutation profiles of viruses analyzed in this study. Viruses were classified into ten mutation-combination types, designated A–J, based on the combination of detected mutations.

| Gene | Mutation | Effect | A | B | C | D | E | F | G | H | I | J |
| --- | --- | --- | --- | --- | --- | --- | --- | --- | --- | --- | --- | --- |
| PB2 | L89V | Increased polymerase activity in mammalian cells | +^*^ | + | + | + | + | + | + | + | + | + |
|  |  | Increased virulence in mice |  |  |  |  |  |  |  |  |  |  |
|  | I292V | Increased polymerase activity in mammalian cells | + | + | + |  | + | + |  | + | + |  |
|  |  | Increased virulence in mice |  |  |  |  |  |  |  |  |  |  |
|  | G309D | Increased polymerase activity in mammalian cells | + | + | + | + | + | + | + | + | + | + |
|  |  | Increased virulence in mice |  |  |  |  |  |  |  |  |  |  |
|  | T339K | Increased polymerase activity in mammalian cells | + | + | + | + | + | + | + | + | + | + |
|  |  | Increased virulence in mice |  |  |  |  |  |  |  |  |  |  |
|  | K389R | Increased polymerase activity in mammalian cells | + | + | + | + | + | + | + | + | + | + |
|  |  | Increased replication in mammalian cells |  |  |  |  |  |  |  |  |  |  |
|  | R477G | Increased polymerase activity in mammalian cells | + | + | + | + | + | + | + | + | + | + |
|  |  | Increased replication in mammalian cells |  |  |  |  |  |  |  |  |  |  |
|  | K482R | Increased polymerase activity in mammalian cells |  |  |  |  |  |  | + |  |  | + |
|  | I495V | Increased polymerase activity in mammalian cells | + | + | + | + | + | + | + | + | + | + |
|  |  | Increased virulence in mice |  |  |  |  |  |  |  |  |  |  |
|  | K627E | Increased polymerase activity in mammalian cells | + | + | + | + | + | + | + | + | + | + |
|  |  | Increased virulence in mice |  |  |  |  |  |  |  |  |  |  |
|  | A676T | Increased polymerase activity in mammalian cells | + | + | + | + | + | + | + | + | + | + |
|  |  | Increased virulence in mice |  |  |  |  |  |  |  |  |  |  |
|  | V598T | Increased polymerase activity in mammalian cells | + | + | + | + | + | + | + | + | + | + |
|  |  | Increased replication in mammalian cells |  |  |  |  |  |  |  |  |  |  |
|  |  | Increased virulence in mice |  |  |  |  |  |  |  |  |  |  |
| PB1 | D3V | Increased polymerase activity in avian cells | + | + | + | + | + | + | + | + | + | + |
|  |  | Increased polymerase activity in mammalian cells |  |  |  |  |  |  |  |  |  |  |
|  |  | Increased replication in avian cells |  |  |  |  |  |  |  |  |  |  |
|  |  | Increased replication in mammalian cells |  |  |  |  |  |  |  |  |  |  |
|  | D622G | Increased polymerase activity in mammalian cells | + | + | + | + | + | + | + | + | + | + |
|  |  | Increased polymerase activity in mice |  |  |  |  |  |  |  |  |  |  |
|  |  | Increased virulence in mice |  |  |  |  |  |  |  |  |  |  |
| PB1-F1 | N66S | Enhanced replication in mice | + | + |  | + | + | + | + | + | + | + |
|  |  | Enhanced virulence in mice |  |  |  |  |  |  |  |  |  |  |
| PA | S37A | Increased polymerase activity in mammalian cells | + | + | + | + | + | + | + | + | + | + |
|  | N383D | Increased polymerase activity in avian cells | + | + | + | + | + | + | + | + | + | + |
|  |  | Increased polymerase activity in mammalian cells |  |  |  |  |  |  |  |  |  |  |
|  | N409S | Increased polymerase activity in mammalian cells |  | + | + | + | + | + | + | + | + | + |
|  |  | Increased replication in mammalian cells |  |  |  |  |  |  |  |  |  |  |
| HA1-5 | K218Q | Increased virus binding to α2-3 | + | + | + | + | + | + | + | + | + | + |
|  |  | Increased virus binding to α2-6 |  |  |  |  |  |  |  |  |  |  |
|  | S223R | Increased virus binding to α2-3 | + | + | + | + | + | + | + | + | + | + |
|  |  | Increased virus binding to α2-6 |  |  |  |  |  |  |  |  |  |  |
|  | S107R | Increased virulence in chickens | + | + | + | + | + | + | + | + | + | + |
|  |  | Increased virulence in mice |  |  |  |  |  |  |  |  |  |  |
|  | T108I | Increased virulence in chickens | + | + | + | + | + | + | + | + | + | + |
|  |  | Increased virulence in mice |  |  |  |  |  |  |  |  |  |  |
|  | T134A | Increased viral replication in mice lungs | + | + | + | + | + | + | + | + | + | + |
|  | S154N | Increased virus binding to α2-6 | + | + | + | + | + | + | + | + | + | + |
|  | N154D | Enhanced replication in mammalian cells | + | + | + | + | + | + | + |  |  |  |
|  | T156A | Increased transmission in guinea pigs | + | + | + | + | + | + | + | + | + | + |
|  |  | Increased virus binding to α2-6 |  |  |  |  |  |  |  |  |  |  |
|  | V182N | Increased virus binding to α2-6 | + | + | + | + | + | + | + | + | + | + |
|  | N189D | Increased virus binding to α2-6 |  |  | + |  | + |  |  |  |  |  |
|  |  | Transmissible among ferrets |  |  |  |  |  |  |  |  |  |  |
| NP | I41V | Increased polymerase activity in mammalian cells |  |  |  |  |  |  |  |  |  | + |
|  | M105V | Increased virulence in chickens |  |  | + | + | + | + | + | + | + | + |
|  | A184K | Increased replication in avian cells | + | + | + | + | + | + | + | + | + | + |
|  |  | Increased virulence in chickens |  |  |  |  |  |  |  |  |  |  |
|  | N319K | Increased polymerase activity in mammalian cells |  |  |  |  |  |  | + |  |  | + |
|  |  | Increased replication in mammalian cells |  |  |  |  |  |  |  |  |  |  |
| M1 | N30D | Increased virulence in mice | + | + | + | + | + | + | + | + | + | + |
|  | I43M | Increased virulence in chickens | + | + | + | + | + | + | + | + | + | + |
|  |  | Increased virulence in ducks |  |  |  |  |  |  |  |  |  |  |
|  |  | Increased virulence in mice |  |  |  |  |  |  |  |  |  |  |
|  | T215A | Increased virulence in mice | + | + | + | + | + | + | + | + | + | + |
| NS-1 | P3S | Enhanced pathogenicity in mice | + | + | + | + | + | + | + | + | + | + |
|  |  | Enhanced replication in mammalian cells |  |  |  |  |  |  |  |  |  |  |
|  | R41K | Enhanced pathogenicity in mice | + | + | + | + | + | + | + | + | + | + |
|  |  | Enhanced replication in mammalian cells |  |  |  |  |  |  |  |  |  |  |
|  | P42S | Decreased antiviral response in mice | + | + | + | + | + | + | + | + | + | + |
|  |  | Increased virulence in mice |  |  |  |  |  |  |  |  |  |  |
|  | K55E | Enhanced replication in mammalian cells | + | + | + | + | + | + | + | + | + | + |
|  | K66E | Enhanced replication in mammalian cells | + | + | + | + | + | + | + | + | + | + |
|  | D74N | Enhanced pathogenicity in mice |  |  |  |  |  |  |  |  | + |  |
|  |  | Enhanced replication in mammalian cells |  |  |  |  |  |  |  |  |  |  |
|  | L103F | Increased virulence in mice | + | + | + | + | + | + | + | + | + | + |
|  | I106M | Increased virulence in mice | + | + | + | + | + | + | + | + | + | + |
|  | C138F | Enhanced replication in mammalian cells | + | + | + | + | + | + | + | + | + | + |
|  |  | Increased viral replication in mammalian cells |  |  |  |  |  |  |  |  |  |  |
|  | V149A | Increased virulence in chickens | + | + | + | + | + | + | + | + | + | + |
|  | N205S | Decreased antiviral response in ferrets | + | + | + | + | + | + | + | + | + | + |
| NS-2 | T48A | Decreased antiviral response in ferrets | + | + | + | + | + | + | + | + | + | + |

^*^+ indicates the presence of the mutation; blank cells indicate the absence of the mutation.
